# Cardiomyocyte cytosolic nuclear self-DNA contributes to the pathogenesis of desmoplakin cardiomyopathy

**DOI:** 10.1172/jci.insight.192283

**Published:** 2025-07-03

**Authors:** Weiyue Wang, Benjamin Cathcart, Quoc D. Nguyen, Loi Q. Lao, Amelia Bryans, Sara E. Coleman, Leila Rouhi, Priyatansh Gurha, Ali J. Marian

**Affiliations:** Center for Cardiovascular Genetics, Institute of Molecular Medicine and Department of Medicine, University of Texas Health Sciences Center at Houston, Houston, Texas, USA.

**Keywords:** Cardiology, Genetics, Heart failure

## Abstract

Hereditary cardiomyopathies are the prototypic forms of heart failure and major causes of sudden cardiac death. The genome in cardiomyopathies is exposed to internal stressors, which damage the DNA and activate the DNA damage response (DDR) pathways. We set out to determine whether the DDR pathways were activated and pathogenic in an established mouse model of desmoplakin (DSP) cardiomyopathy generated upon deletion of the *Dsp* gene in cardiomyocytes (*Myh6-MerCreMer^Tam^ Dsp*^fl/fl^; *Myh6-Mcm*^Tam^ *Dsp*^fl/fl^). The mice exhibited premature death, cardiac dysfunction, myocardial cell death, fibrosis, and increased expression levels of the pro-inflammatory cytokines, consistent with the phenotype of human DSP cardiomyopathy. Cytosolic nuclear self-DNA (nDNA) and mitochondrial DNA (mtDNA) were increased in cardiomyocyte cytosol in the *Myh6-Mcm*^Tam^ *Dsp*^fl/fl^ mice. Likewise, the DDR pathway proteins, including the cyclic GMP-AMP synthase (CGAS)/stimulator of interferon response 1, were upregulated, as were the transcript levels of interferon response factor 3 and the NF-κB target genes. Deletion of the *Mb21d1* gene encoding CGAS in the *Myh6-Mcm*^Tam^ *Dsp*^fl/fl^ mice prolonged survival, improved cardiac function, attenuated fibrosis, and reduced cell death. Thus, cytosolic nDNA and mtDNA are increased and the DDR pathways are activated and pathogenic in a mouse model of DSP cardiomyopathy, whereas genetic blockade of CGAS is salubrious.

## Abstract 2

We report increased self-DNA in cardiac myocyte cytosol, activation of the DNA damage response pathway, and the beneficial effects of blocking the cytosolic component of the DNA damage response pathway in a mouse model of desmoplakin-cardiomyopathy, which pro-inflammatory and pro-fibrotic cardiomyopathy with excess myocardial cell death.

## Graphical abstract

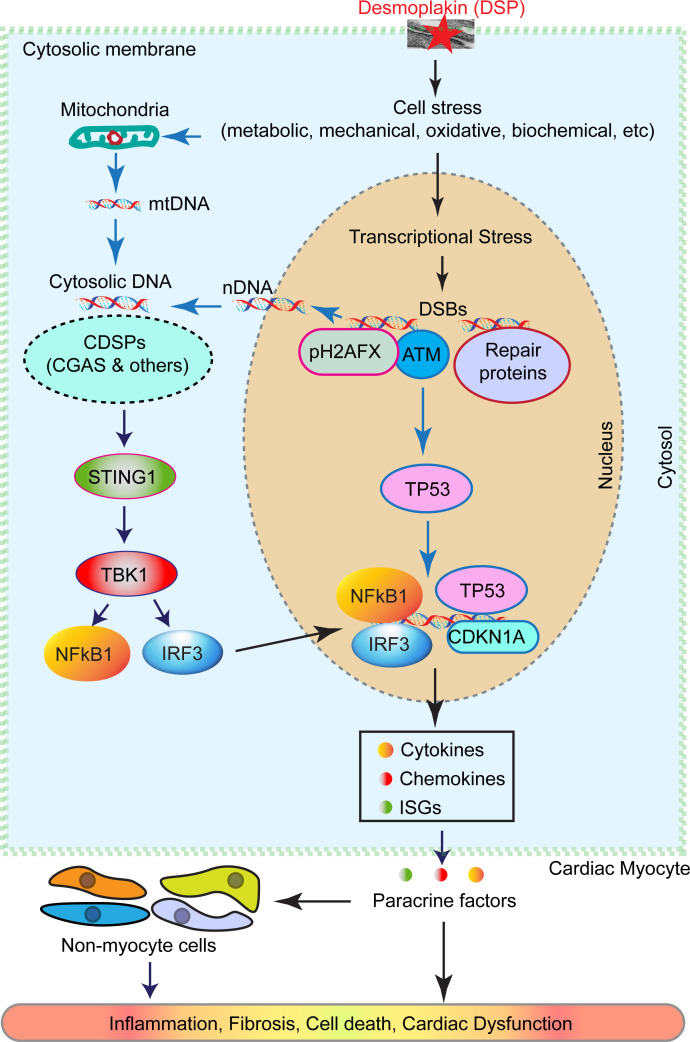

## Introduction

Hereditary cardiomyopathies are the primary diseases of cardiomyocytes and caused by mutations in genes encoding proteins involved in cardiomyocyte structure and function (1, 2). A subset of cardiomyopathies is caused by heterozygous and homozygous mutations in the *DSP* gene, encoding desmoplakin, a constituent of the desmosomes in the intercalated disks (3–5). The phenotype of DSP cardiomyopathy straddles between arrhythmogenic cardiomyopathy (ACM), wherein cardiac arrhythmias are the cardinal features and there is a predilection toward predominant involvement of the right ventricle, and dilated cardiomyopathy (DCM), which mainly manifests with left ventricular (LV) dysfunction and heart failure (3–5). The ambiguous phenotype likely reflects the topographic positioning of DSP in the inner side of the cytoplasmic membrane, where it interacts with transmembrane desmosome proteins, which are responsible for ACM, and with intermediary cytoskeletal proteins, which are known to cause DCM (1, 6, 7). Notwithstanding the ambiguity, the phenotype of DSP cardiomyopathy is characterized by extensive cell death (PANoptosis), severe myocardial fibrosis, and inflammation (3, 4, 8).

The complexity of the clinical phenotype in cardiomyopathies suggests the involvement of multiple pathogenic pathways, a notion that is supported by the evidence of dysregulation of the canonical WNT, Hippo, nuclear factor κB1 (NF-κB1), and ubiquitin/proteasome pathways, as well as the metabolic pathways, among others (9–13). The dysregulated pathways impose considerable oxidative, metabolic, biological, and mechanical stresses on the nuclear and mitochondrial genomes, leading to DNA damage and impaired proper genomic functions (14). The most consequential form of DNA damage is double-stranded breaks (DSBs) and the resultant activation of the DNA damage response (DDR) pathways, which were recently implicated in the pathogenesis of cardiovascular diseases (15–18). Activation of the DDR in the nucleus leads to 2 partially distinct DNA repair and cell cycle arrest pathways, the former through the recruitment of the repair proteins to the sites of the damage and the latter through the activation of ataxia-telangiectasia mutated (ATM)/tumor protein 53 (TP53) pathway (14). The release of a subset of the damaged DNA, whether nuclear DNA (nDNA) or mitochondrial DNA (mtDNA), into the cytosol activates the cytosolic DNA-sensing proteins (CDSPs), such as cyclic GMP-AMP synthase (CGAS), which generates 2′ 3′-cyclic guanosine monophosphate-adenosine monophosphate (cGAMP) (19, 20). The newly generated cGAMP binds to the stimulator of interferon response 1 (STING1) and activates the downstream molecules TANK-binding kinase 1 (TBK1) and subsequently interferon response factor 3 (IRF3) and NF-κB transcription factors (19, 21, 22). Activation of IRF3 and NF-κB leads to type I interferon response and the expression of interferon-stimulated genes (ISGs) and pro-inflammatory cytokines.

We determined the activation and the role of the CDSP pathway, also known as the CGAS/STING1 pathway, in the pathogenesis of DSP cardiomyopathy.

## Results

### Phenotypic data.

Phenotypic characteristics of the mouse models used to detect cytosolic DNA and their effects of activation of the CDSP (CGAS/STING1) pathway on cardiac phenotype have been published (8, 13, 23, 24). The wild-type (WT) and *Myh6-MerCreMer^Tam^* (*Myh6-Mcm*^Tam^) mice were included as controls. There was no discernible functional or histological phenotype in *Myh6-Mcm*^Tam^ mice at 4 weeks of age, which is the time point used in the present studies (8, 13, 23). Nevertheless, because tamoxifen, at moderate and high doses, and the long-term expression of the Cre recombinase, could impart cardiotoxicity, the *Myh6-Mcm*^Tam^ mice were included as controls in the main experiments (25–27).

The CGAS-deficient (*Mb21d1*^–/–^) mice do not show a discernible phenotype and survive normally without evidence of cardiac dysfunction, cardiac arrhythmias, myocardial fibrosis, or cell death (24, 28).

The *Myh6-Mcm*^Tam^ *Dsp*^fl/fl^ mice show severe cardiac dysfunction, cardiac arrhythmias, extensive myocardial fibrosis, and PANoptosis and die prematurely, typically within 4 to 6 weeks of age, as published (8, 13).

### Detection of cytosolic nDNA.

**Figure 1:**
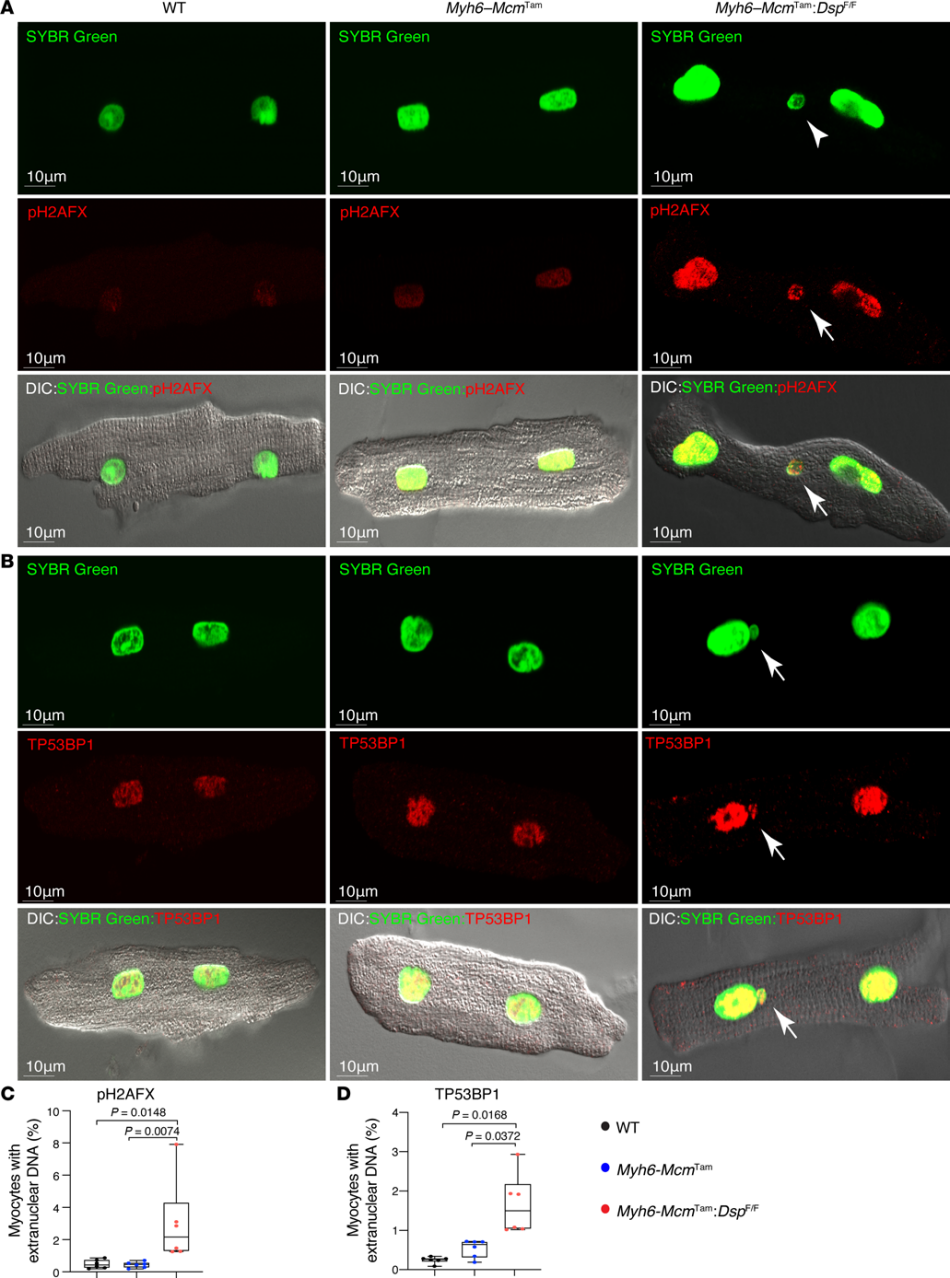
Detection of cytosolic nDNA stained with phosphorylated H2AFX or TP53BP1 in cardiomyocytes. (**A**) Immunofluorescence (IF) staining of isolated cardiomyocytes from wild-type (WT), *Myh6-Mcm*^Tam^, and *Myh6-Mcm*^Tam^ *Dsp*^fl/fl^ mice stained for DNA with SYBR Green and phosphorylated (p-) H2A histone family member X (pH2AFX). Individual IF panels are shown along with the panels superimposed on the differential interference contrast (DIC) panel. The arrow shows cytosolic (extranuclear) nDNA stained positive for pH2AFX. (**B**) IF panels showing cardiomyocytes stained for DNA (SYBR Green) and the DNA damage marker tumor protein 53 binding protein 1 (TP53BP1). (**C**) Quantitative data representing cytosolic nDNA stained with pH2AFX depicted along with the corrected pairwise *P* values obtained by Dunn’s test. Only *P* values < 0.05 are shown. (*N* = 6 mice per genotype.) (**D**) Quantitative data representing cytosolic nDNA stained with TP53BP1. Data were analyzed by Welch’s ANOVA tests followed by Dunnett’s T3 test for multiple comparisons (*N* = 6 mice per genotype).

**Figure 2:**
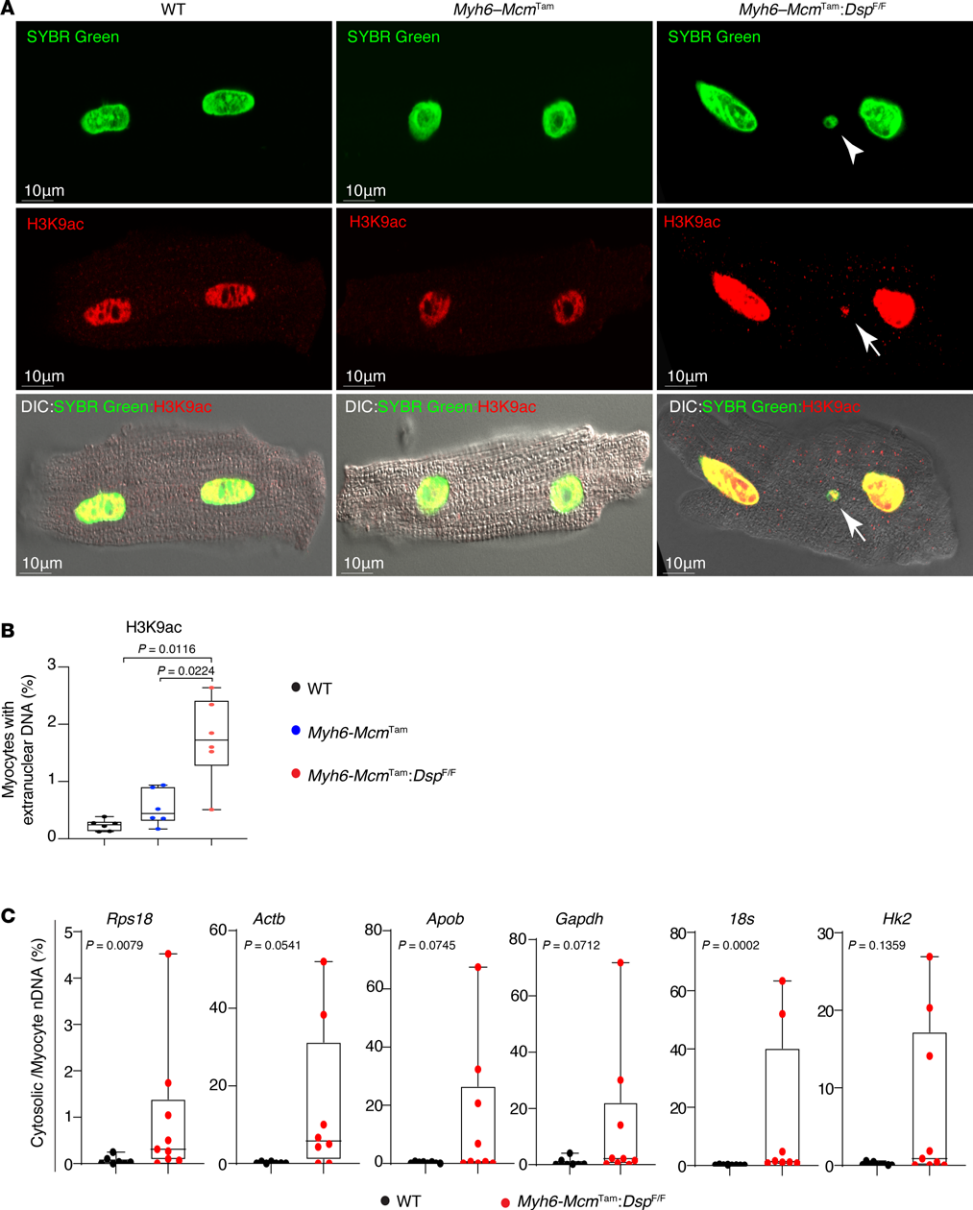
Detection of cytosolic nDNA stained with H3K9ac in cardiomyocytes. (**A**) Myocytes stained for acetylated histone 3 lysine 9 (H3K9ac) and DNA (SYBR Green). Arrows point to cytosolic nDNA. (**B**) Quantitative data representing data for cytosolic nDNA stained with H3K9ac (*N* = 6 mice per genotype). (**C**) Dot blots showing the individual data points, the median value, and the 95% CI of PCR amplification of selected nuclear genes in the cytosolic DNA extracts from cardiomyocytes in the WT and *Myh6-Mcm*^Tam^ *Dsp*^fl/fl^ groups. The cytosolic nDNA is presented as the percentage of the nuclear DNA and compared between the 2 genotypes by the Mann-Whitney test. Genotypes are denoted by different colors, and the same color is used throughout all panels in each figure.

To detect cytosolic nDNA, cardiomyocytes were stained with SYBR Green to detect DNA and 3 protein markers of DSBs, namely phosphorylated (p-) H2AFX, tumor protein 53 binding protein 1 (TP53BP1), and histone 3 lysine 4 acetylation (H3K9ac). The cytosolic nDNA stained for the expression of p-H2AFX was detected in 1.74% of cardiomyocytes in the *Myh6-Mcm*^Tam^ *Dsp*^fl/fl^ mice as opposed to 0.23% and 0.54% of the myocytes in the WT and *Myh6-Mcm*^Tam^ control myocytes, respectively (Figure 1, A and C). Likewise, TP53BP1-stained cytoplasmic nDNA was detected in 1.65% of the cardiomyocytes in the *Myh6-Mcm*^Tam^ *Dsp*^fl/fl^ mice and 0.24% and 0.54% of the myocytes in the WT and *Myh6-Mcm*^Tam^ genotypes (Figure 1, B and D). The findings were similar when the cytosolic nDNA was detected upon staining for H3K9ac, showing a 3- to 7-fold higher prevalence in the *Myh6-Mcm*^Tam^ *Dsp*^fl/fl^ as compared with the WT and *Myh6-Mcm*^Tam^ myocytes (Figure 2, A and B).

The presence of cytosolic nDNA in cardiomyocytes also was investigated by PCR amplification of the cytosolic DNA extracts for selected nuclear genes. The findings indicated increased cytosolic nDNA in cardiomyocytes isolated from the *Myh6-Mcm*^Tam^ *Dsp*^fl/fl^ mice as compared with myocytes isolated from the WT mice (Figure 2C).

### Detection of cytosolic mtDNA.

**Figure 3:**
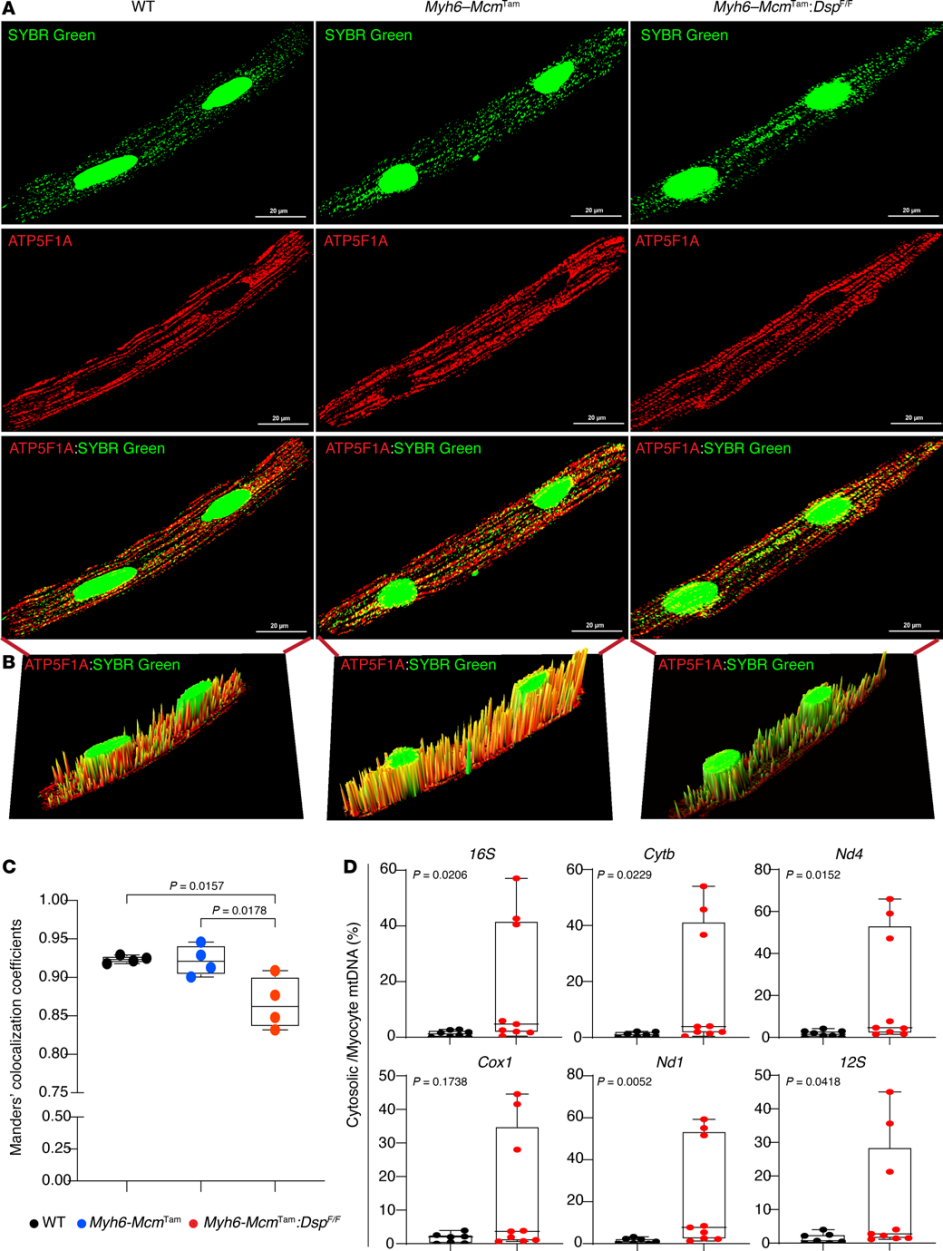
Detection of cytosolic mtDNA in cardiomyocytes. (**A**) Confocal IF panels showing individual cardiomyocytes stained for DNA with SYBR Green and mitochondrial protein ATP5F1A along with the overlay in the WT, *Myh6-Mcm*^Tam^, and *Myh6-Mcm*^Tam^ *Dsp*^fl/fl^ genotypes. (**B**) Spectral display of the chromophore in merged image in the 3 genotypes. The extra-mitochondrial cytoplasmic mtDNA was identified by staining of DNA with SYBR Green but not with ATP5F1A after excluding the nucleus. (**C**) The spectral overlaps between the SYBR Green and ATP5F1A were calculated in at least 6 to 9 myocytes and 4 hearts per genotype and were displayed as mean and SD. The differences were compared by ANOVA followed by pairwise comparison by Tukey’s method. Only the *P* values less than 0.05 are depicted in the graph. (**D**) Dot blots showing the individual data points, the median value, and the 95% CI of PCR amplification of selected mitochondrial genes in the cytosolic DNA extracts from cardiomyocytes in the WT and *Myh6-Mcm*^Tam^ *Dsp*^fl/fl^ mice. The cytosolic mtDNA is presented as the percentage of total cellular DNA and compared between the 2 genotypes by the Mann-Whitney test.

To detect mtDNA, isolated cardiomyocytes were stained with SYBR Green to mark the DNA and mitochondrial protein ATP synthase F1 subunit alpha (ATP5F1A). Four other mitochondrial proteins, namely, translocase of outer mitochondrial membrane 20; mitochondrial transcription termination factor 3; transcription factor A, mitochondria; and voltage-dependent anion-selective channel 1, were also tested. Among the 4 mitochondrial proteins, staining for ATP5F1A had the best signal-to-noise ratio and was the most consistent. Therefore, it was used to detect mitochondria. The approach is based on the premise that the cytosolic DNA, stained for SYBR Green outside of the nucleus but not stained for mitochondrial protein ATP5F1A, represents free cytosolic mtDNA. The fluorescence spectral displays of SYBR Green and ATP5F1A antibody were overlapped, and the areas under the overlapped signals, excluding the nDNA regions, were compared among the groups (Figure 3, A and B). The area of the overlap was notably smaller, as there was a higher SYBR Green signal in the *Myh6-Mcm*^Tam^ *Dsp*^fl/fl^ cardiomyocytes than in the WT and *Myh6-Mcm*^Tam^ myocytes, whereas there were no differences between between the WT and *Myh6-Mcm*^Tam^ (Figure 3C).

To test for corroboration of the findings, extracted cytosolic DNA from cardiomyocytes was analyzed by PCR for the presence of selected mitochondrial genes. As shown in Figure 3D, mtDNA was detected in the cytosolic DNA extracts in myocytes in about half of the *Myh6-Mcm*^Tam^ *Dsp*^fl/fl^ mice, and the mean levels of the mtDNA of several mitochondrial genes were increased in the *Myh6-Mcm*^Tam^ *Dsp*^fl/fl^ cardiomyocytes as compared with the WT group.

### Activation of the DDR pathways in cardiomyocytes.

**Figure 4:**
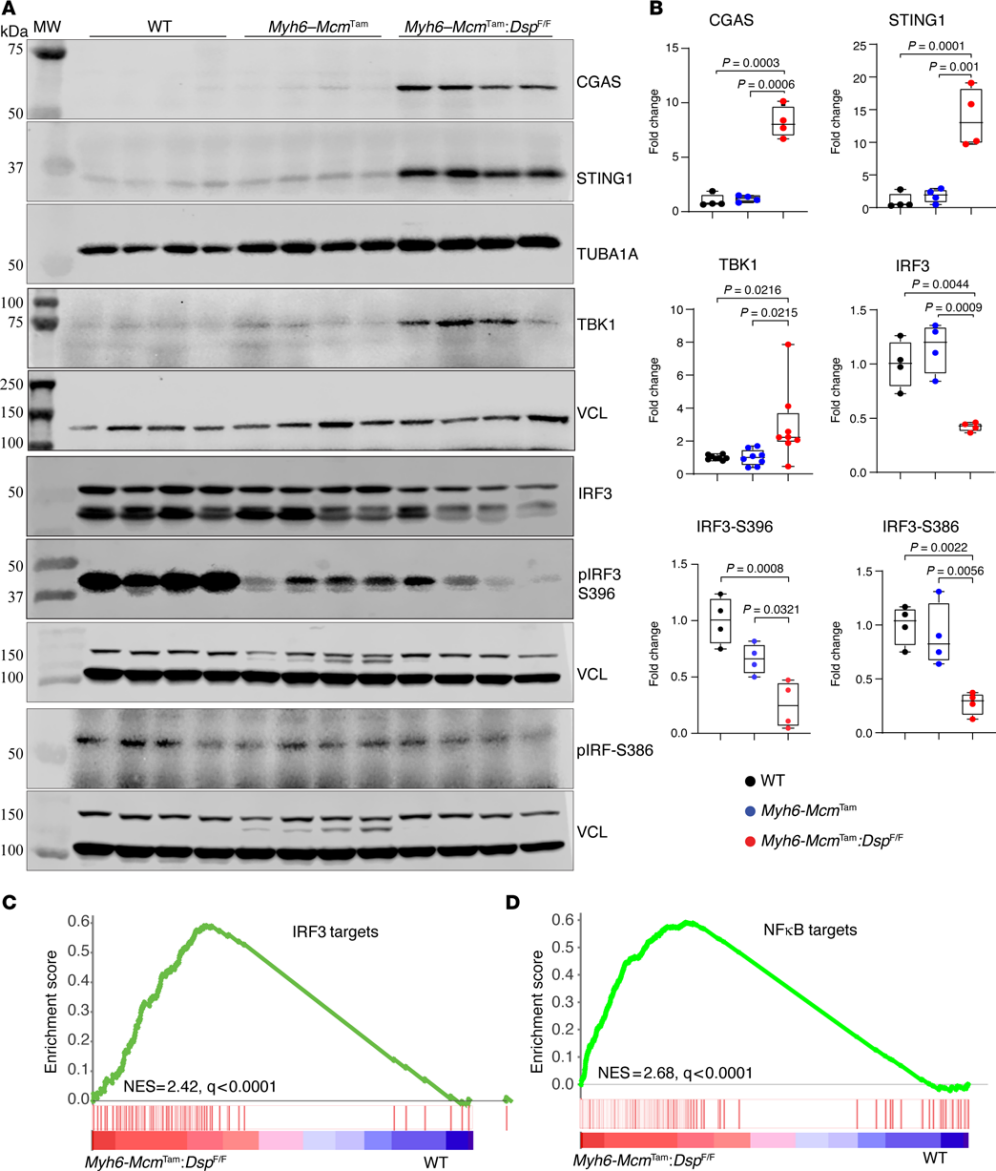
Activation of the CDSP pathway in the *Myh6-Mcm*^Tam^ *Dsp*^fl/fl^ cardiomyocytes. (**A**) Immunoblots showing expression of the selected proteins in the CDSP pathway in the WT, *Myh6-Mcm*^Tam^, and *Myh6-Mcm*^Tam^ *Dsp*^fl/fl^ cardiomyocytes. (**B**) Dot plots showing the individual data points, median, and 95% CI representing the expression levels of the selected proteins shown in **A**. Data were compared among the 3 genotypes using ANOVA followed by pairwise comparisons per Bonferroni method (*N* = 4 to 8 per group). Only *P* values < 0.05 are depicted. (**C**) GSEA plot showing enrichment of interferon regulatory factor 3 (IRF3) target genes in the *Myh6-Mcm*^Tam^ *Dsp*^fl/fl^ myocytes compared with the WT. The normalized enrichment score (NES) and the *q* value are depicted. (**D**) GSEA plot showing enrichment of NF-κB target genes in the *Myh6-Mcm*^Tam^ *Dsp*^fl/fl^ myocytes compared with the WT. NES and the *q* value are depicted.

To determine whether increased cytosolic DNA leads to the activation of the CDSP, also known as the CGAS/STING1, pathway in cardiomyocytes in the *Myh6-Mcm*^Tam^ *Dsp*^fl/fl^ mice, expression levels of several key proteins in the CDSP pathway were analyzed by immunoblotting. The CGAS level was increased in *Myh6-Mcm*^Tam^ *Dsp*^fl/fl^ cardiomyocytes, whereas CGAS was barely detected in the cardiomyocytes isolated from the WT and *Myh6-Mcm*^Tam^ mice (Figure 4, A and B). Likewise, levels of STING1 and TBK1, the downstream partners of CGAS in the CDSP pathway, were upregulated in the *Myh6-Mcm*^Tam^ *Dsp*^fl/fl^ cardiomyocytes (Figure 4, A and B). The level of total IRF3, which is targeted for activation by TBK1 through a 2-step phosphorylation, was reduced in *Myh6-Mcm*^Tam^ *Dsp*^fl/fl^ cardiomyocytes (Figure 4, A and B). Similarly, the expression levels of p-S396 IRF3, which is the autoinhibitory site, and p-S386 IRF3, which is considered the activation site, were also reduced in the *Myh6-Mcm*^Tam^ *Dsp*^fl/fl^ cardiomyocytes (Figure 4, A and B) (29).

The downstream effects of the activation of the CGAS/STING1 pathway are increased expression of the ISGs through IRF3 and pro-inflammatory genes through NF-κB transcriptional regulators (30, 31). Gene set enrichment analysis (GSEA) of the cardiomyocyte transcripts in the RNA-Seq data showed enrichment of the IRF3 and NF-κB target genes in the *Myh6-Mcm*^Tam^ *Dsp*^fl/fl^ cardiomyocytes (Figure 4, C and D). Consistent with the increased transcript levels of the IRF3 and NF-κB target genes, genes involved in inflammation, apoptosis, and epithelial-mesenchymal transition were enriched in the *Myh6-Mcm*^Tam^ *Dsp*^fl/fl^ cardiomyocytes.

**Figure 5:**
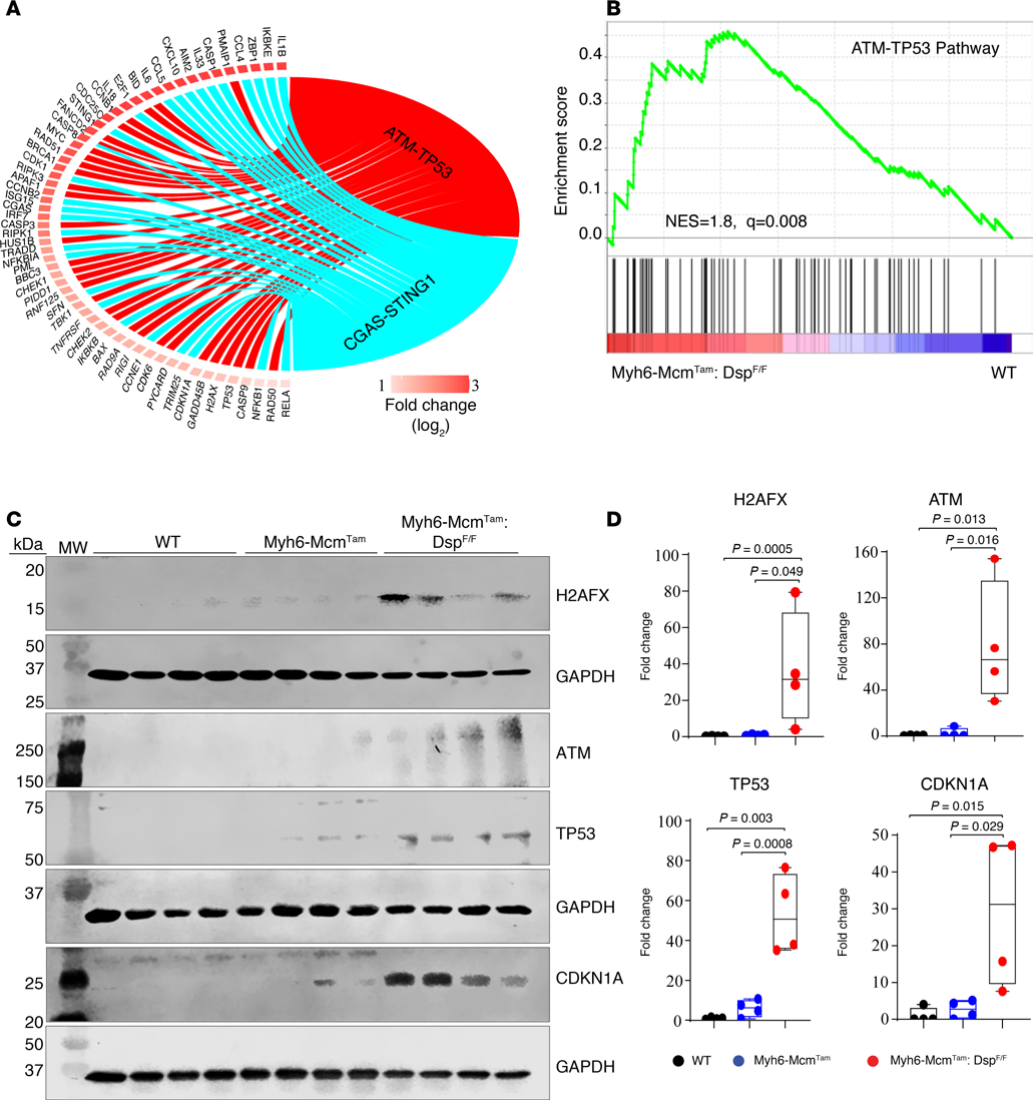
Activation of the ATM/TP53 pathway in the *Myh6-Mcm*^Tam^ *Dsp*^fl/fl^ cardiomyocytes. (**A**) Circos map of the differentially expressed genes showing activation of the DDR ATM/TP53 and the CDSP CGAS/STING1 pathways in the *Myh6-Mcm*^Tam^ *Dsp*^fl/fl^ (compared with the WT) myocytes. Fold change in gene expression is depicted as log_2_ units. (**B**) GSEA plot showing enrichment of TP53 target genes in the *Myh6-Mcm*^Tam^ *Dsp*^fl/fl^ myocytes compared with the WT. NES and the *q* value are depicted. (**C**) Immunoblot of selected proteins in the nuclear cell cycle checkpoint DDR pathway. (**D**) Dot plots showing individual data points, median, and 95% CI of the data on the expression levels of the selected proteins in the nuclear DDR pathway. ANOVA *P* values are shown along with Bonferroni-corrected pairwise *P* values (*N* = 4 per group).

A Circos map of the RNA-Seq data suggested activation of the ATM/TP53 pathway in *Myh6-Mcm*^Tam^ *Dsp*^fl/fl^ cardiomyocytes, a finding that was corroborated by GSEA, which showed enrichment of the genes involved in the ATM/TP53 pathway (Figure 5, A and B). To substantiate these findings, expression levels of selected proteins in the ATM/TP53 pathway were analyzed by Western blotting, which showed increased levels of H2AFX, ATM, TP53, and its downstream effector cyclin-dependent kinase inhibitor 1A (CDKN1A) in the *Myh6-Mcm*^Tam^ *Dsp*^fl/fl^ cardiomyocytes (Figure 5, C and D).

### Genetic deletion of the Mb21d1 gene in the Myh6-Mcm^Tam^ Dsp^fl/fl^ mice.

**Figure 6:**
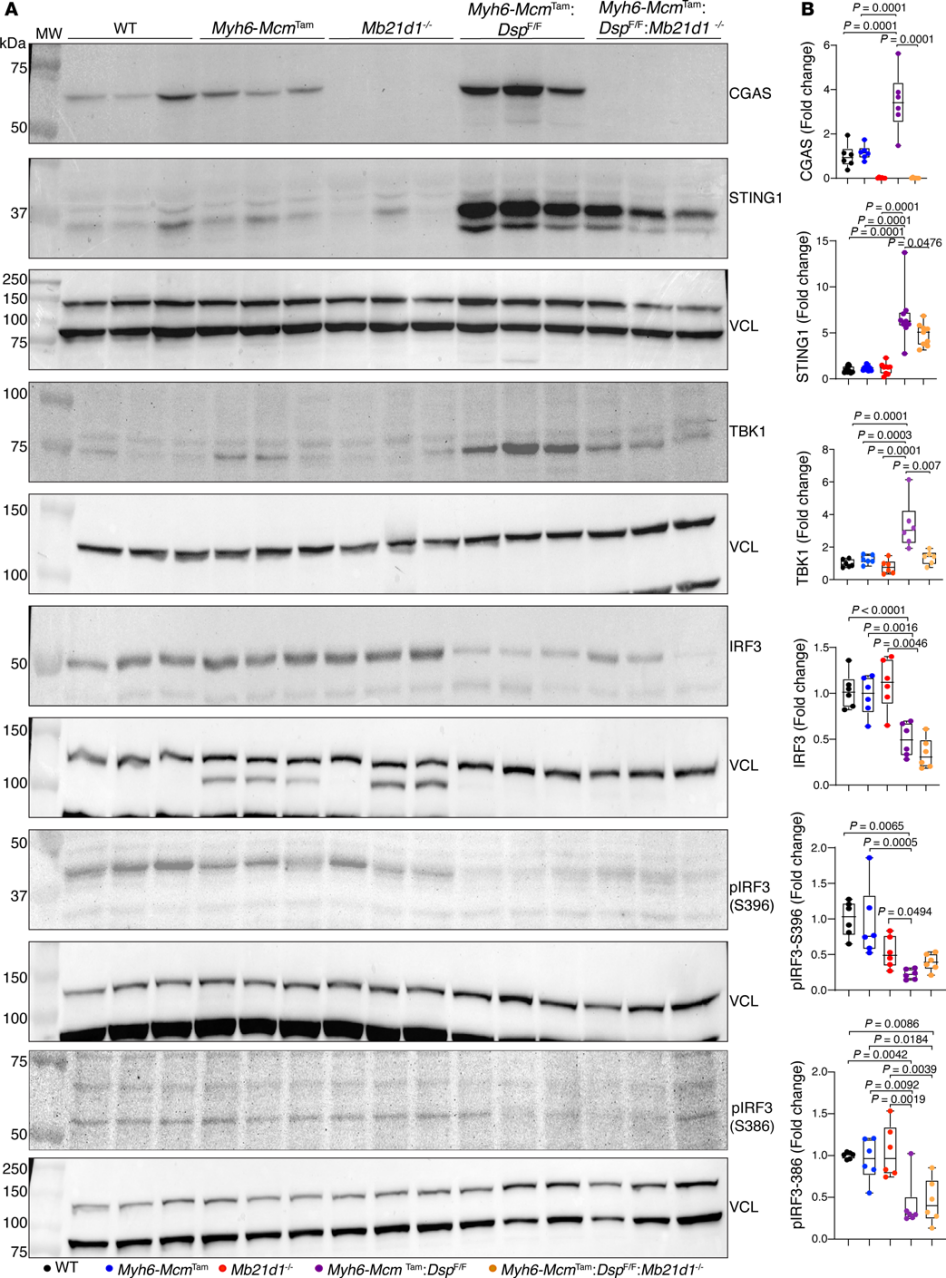
Genetic blockade of the CDSP pathway by deleting the *Mb21d1* (CGAS) gene in the *Myh6-Mcm*^Tam^ *Dsp*^fl/fl^ mice. (**A**) Immunoblots showing the expression levels of selected protein members of the CDSP pathway in the WT, *Myh6-Mcm*^Tam^, *Mb21d1*^–/–^, *Myh6-Mcm*^Tam^ *Dsp*^fl/fl^, and *Myh6-Mcm*^Tam^ *Dsp*^fl/fl^ *Mb21d1*^–/–^ mice. (**B**) Dot plots showing the individual data points, median, and 95% CI of the quantitative data representing the expression levels of the selected proteins shown in **A** (*N* = 6 to 9 per group). Statistical analysis was performed by ANOVA followed by pairwise comparisons per Bonferroni’s method. Genotypes are denoted by color and the same color is used throughout all panels in each figure.

To block the CDSP pathway, the *Mb21d1* gene, encoding CGAS, was deleted in the *Myh6-Mcm*^Tam^ *Dsp*^fl/fl^ mice by crossing the *Myh6-Mcm*^Tam^ *Dsp*^fl/fl^ and *Mb21d1*^–/–^ mice and treating the offspring with tamoxifen. The effective deletion of the CGAS protein was analyzed by immunoblotting, which showed nearly undetectable levels of the CGAS protein in the *Mb21d1*^–/–^ and *Myh6-Mcm*^Tam^ *Dsp*^fl/fl^ *Mb21d1*^–/–^ hearts, as opposed to its increased levels in the *Myh6-Mcm*^Tam^ *Dsp*^fl/fl^ hearts (Figure 6, A and B). Likewise, expression levels of STING1 and TBK1 proteins, downstream of CGAS in the CDSP pathway, were increased in the *Myh6-Mcm*^Tam^ *Dsp*^fl/fl^ whereas the levels of both proteins were attenuated in the *Myh6-Mcm*^Tam^ *Dsp*^fl/fl^ *Mb21d1*^–/–^ hearts. The level of the total IRF3, which was reduced in the *Myh6-Mcm*^Tam^ *Dsp*^fl/fl^ hearts, was not affected by the deletion of the *Mb21d1* gene (Figure 6, A and B) (24, 29). Similarly, the level of p-IRF3-S396, located at the autoinhibitory domain, was also reduced in the *Myh6-Mcm*^Tam^ *Dsp*^fl/fl^ and remained low in *Myh6-Mcm*^Tam^ *Dsp*^fl/fl^ *Mb21d1*^–/–^ hearts (Figure 6, A and B). Likewise, the level of p-IRF3 (S386) was reduced in the *Myh6-Mcm*^Tam^ *Dsp*^fl/fl^ and remained reduced in the *Myh6-Mcm*^Tam^ *Dsp*^fl/fl^ *Mb21d1*^–/–^ hearts (Figure 6, A and B) (29).

### Survival in the Myh6-Mcm^Tam^ Dsp^fl/fl^ mice lacking CGAS (Myh6-Mcm^Tam^ Dsp^fl/fl^ Mb21d1^–/–^ mice).

**Figure 7:**
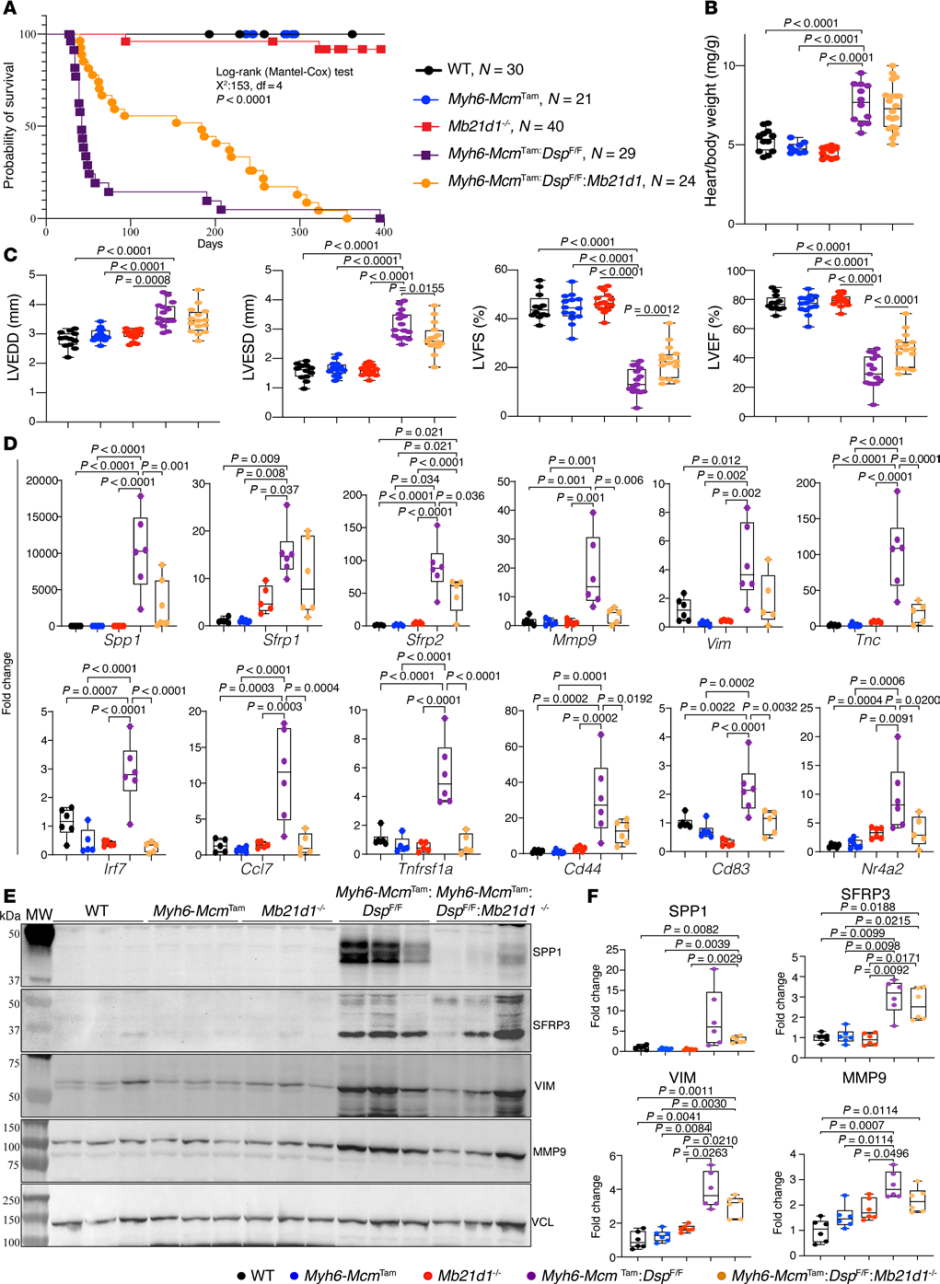
Effects of deletion of *Mb21d1* gene on survival, cardiac function, and expression of heart failure biomarkers. (**A**) Kaplan-Meier survival plots in mice in 5 control and experimental groups. Each data point identifies 1 mouse alive at that time point, and each vertical drop indicates a death (*N* = 21 to 40 per genotype). The survival rates were compared by the log-rank test. (**B**) Dot plots showing heart weight/body weight ratio in the experimental groups (*N* = 17 to 21 mice per group). The *P* values were calculated by ANOVA and pairwise comparisons by the Bonferroni method. Only *P* values < 0.05 are depicted. (**C**) Selected echocardiographic indices of cardiac size and function are shown as dot plots in the 5 groups. Data on left ventricular end-diastolic diameter (LVEDD), LV end-systolic diameter (LVESD), LV fractional shortening (LVFS), and LV ejection fraction (LVEF) are depicted. The differences were compared by ANOVA followed by pairwise comparison by the Bonferroni method. Only *P* values < 0.05 are shown (*N* = 10 to 15 per genotype). (**D**) Dot plots showing cardiomyocyte transcript levels of a dozen selected genes that are the targets of IRF3 or NF-κB1 and are considered biomarkers for heart failure. Data are normalized to *Vcl* levels and shown as a fold change to that in the WT mice. (**E**) Immunoblots showing expression levels of selected proteins in the control and experimental groups (*N* = 6 per group). (**F**) Quantitative dot blot data representing the blot shown in **E**. The data are normalized to the VCL levels and shown as fold change. The differences among the groups are compared by Welch’s ANOVA followed by pairwise comparison by Dunnett’s T3 multiple comparisons. Only significant *P* values are shown. Each dot represents 1 independent sample (*N* = 6 per genotype).

The survival rates, up to 400 days, were compared among mice in the 3 control and 2 experimental groups. As reported previously, the *Mb21d1*^–/–^ and *Myh6-Mcm*^Tam^ mice did not show a discernible cardiac phenotype at the baseline and had survival rates comparable to those of the WT mice (Figure 7A) (8, 13, 23, 28). The *Myh6-Mcm*^Tam^ *Dsp*^fl/fl^ mice had a high mortality rate, as approximately 90% of the mice died within 3 months of age, and the median survival was 42 days (Figure 7A) (8). Deletion of the *Mb21d1* gene (CGAS) prolonged the survival rate of the *Myh6-Mcm*^Tam^ *Dsp*^fl/fl^ (*Myh6-Mcm*^Tam^ *Dsp*^fl/fl^ *Mb21d1*^–/–^), as indicated by a prolonged median survival from 42 to 184 days (ratio 4.38, 95% CI 2.5 to 7.8, Figure 7A). None of the mice with the compound *Myh6-Mcm*^Tam^ *Dsp*^fl/fl^ *Mb21d1*^–/–^ genotype survived beyond 1 year of age (Figure 7A).

### Cardiac size and function.

The heart weight corrected for the body weight was increased in the *Myh6-Mcm*^Tam^ *Dsp*^fl/fl^ mice and remained increased in the *Myh6-Mcm*^Tam^ *Dsp*^fl/fl^ *Mb21d1*^–/–^ mice (Figure 7B). The echocardiographic indices of cardiac size and function were within the normal range in the WT, *Myh6-Cre*, and *Mb21d1*^–/–^ mice, but indices of cardiac size were increased and those of cardiac systolic function were reduced in the *Myh6-Mcm*^Tam^ *Dsp*^fl/fl^ mice (Table 1 and Figure 7C) (8, 13). Deletion of the *Mb21d1*^–/–^ gene reduced LVESD and improved cardiac function, as indicated by an increased LVFS and LVEF in the *Myh6-Mcm*^Tam^ *Dsp*^fl/fl^ *Mb21d1*^–/–^ compared with the corresponding indices in the *Myh6-Mcm*^Tam^ *Dsp*^fl/fl^ mice (Figure 7C). Complete echocardiographic data are presented in Table 1.

Transcript levels of selected markers of heart failure that are also targets of IRF3 and NF-κB transcription factors were quantified by reverse transcription PCR (RT-PCR) in cardiac RNA extracts from the control and experimental mice. Transcript levels of several established biomarkers, including *Spp1*, *Sfrp1*, *Sfrp2*, *Tnc*, *Vim*, *Mmp9*, and *Tnfrsf1a*, and several inflammatory markers that are known to be activated in heart failure, such as *Cd44*, *Cd83*, *Ccl7*, *Irf7*, and *Nr4a2*, were upregulated in the *Myh6-Mcm*^Tam^ *Dsp*^fl/fl^ cardiomyocytes (Figure 7D). Consistent with the attenuation of cardiac dysfunction in the *Myh6-Mcm*^Tam^ *Dsp*^fl/fl^ *Mb21d1*^–/–^ mice, transcript levels of several heart failure biomarkers, composed of those regulated by IRF3 and NF-κB transcription factors, were attenuated upon deletion of *Mb21d1* gene in the *Myh6-Mcm*^Tam^ *Dsp*^fl/fl^ *Mb21d1*^–/–^ mouse model of DSP cardiomyopathy (Figure 7D).

To further corroborate the findings, the expression levels of the selected dysregulated heart failure biomarkers, namely SPP1, SFRP3, VIM, and MMP9, were assessed in cardiac protein extracts by immunoblotting. In agreement with the RT-PCR data, levels of SPP1, SFRP3, VIM, and MMP9 were increased in the *Myh6-Mcm*^Tam^ *Dsp*^fl/fl^ as compared with the WT or *Myh6-Mcm*^Tam^ mice (Figure 7, E and F). The deletion of the *Mb21d1* gene, encoding CGAS, attenuated the elevated levels of SPP1, SFRP3, VIM, and MMP9 in the *Myh6-Mcm*^Tam^ *Dsp*^fl/fl^ *Mb21d1*^–/–^ mice (Figure 7, E and F).

**Figure 8:**
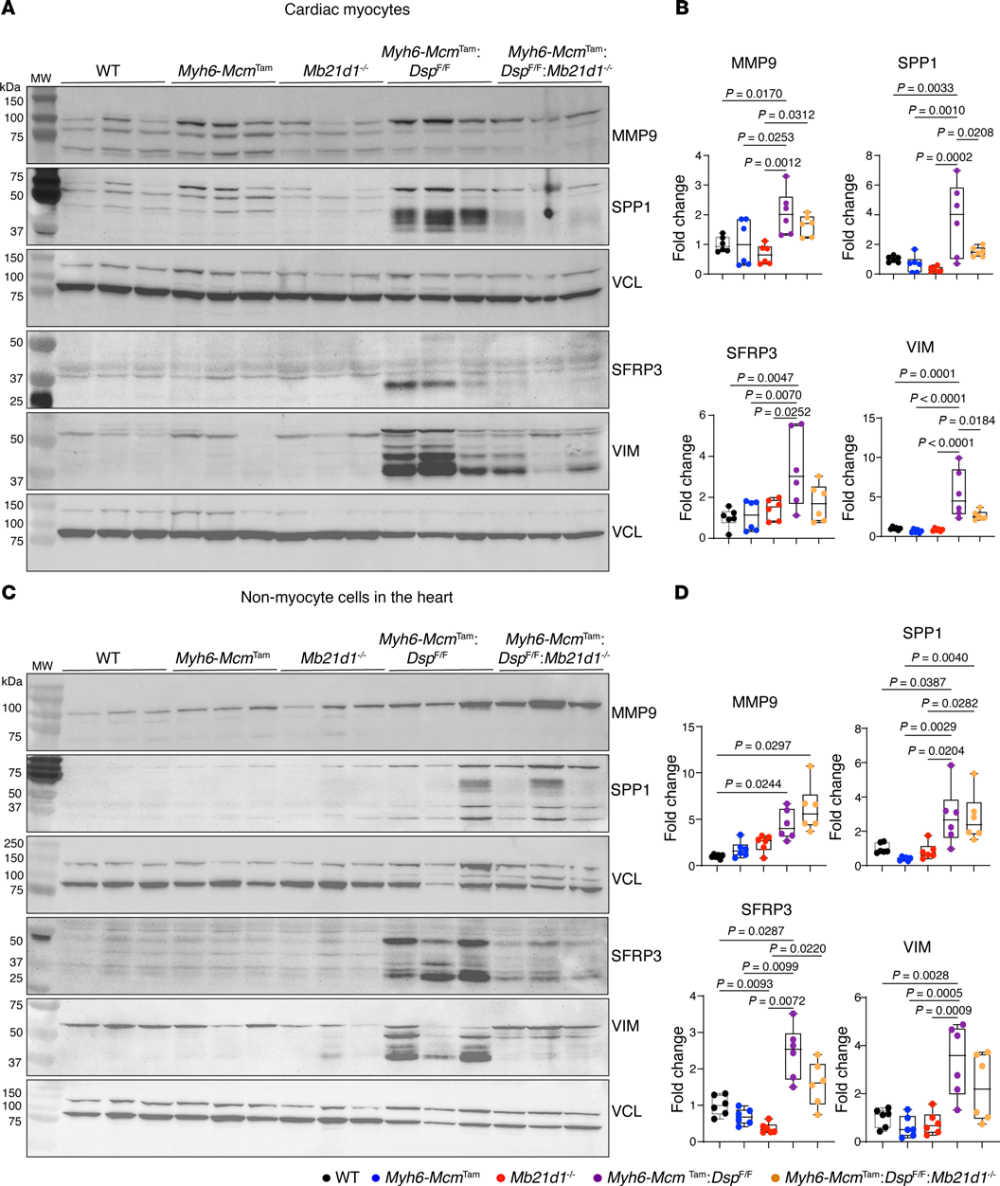
Effects of deletion of *Mb21d1* gene on the expression levels of selected heart failure biomarkers in cardiomyocytes and nonmyocytes. (**A**) Immunoblots showing expression levels of matrix metalloproteinase-9 (MMP9); secreted phosphoprotein 1, also known as osteopontin (SPP1); secreted frizzled-related protein 3 (SFRP3); and vimentin (VIM) in cardiomyocytes in the control and experimental groups. (**B**) Quantitative data representing the blots shown in **A**. Individual data points, the mean, and 95% CIs are shown. The *P* values are calculated by ANOVA followed by pairwise comparisons. Only *P* values < 0.05 are shown. (**C**) Immunoblots showing expression levels of MMP9, SPP1, SFRP3, and VIM in nonmyocyte cells in the heart in the control and experimental groups. (**D**) Quantitative data representing the blots in **C**. Data are shown as described in **B**.

Because we used systemic deletion of the *Mb21d1* gene in these experiments and given that SPP1, SFRP3, VIM, and MMP9 are also expressed in nonmyocyte cells in the heart, including immune cells, we determined the expression levels of these selected markers of heart failure in cardiomyocyte and nonmyocyte cells in the heart. As shown in Figure 8, expression levels of MMP9, SPP1, SFRP3, and VIM were increased in cardiomyocytes as well as in nonmyocyte cells isolated from the *Myh6-Mcm*^Tam^ *Dsp*^fl/fl^ mouse heart. Systemic deletion of the *Mb21d1* gene partially attenuated expression levels of these markers in cardiomyocytes, except for MMP9 levels, which were unchanged (Figure 8, A and B). Deletion of the *Mb21d1* gene did not change the expression levels of MMP9, SPP1, SFRP3, and VIM in nonmyocyte cells in the heart (Figure 8).

### Cardiac arrhythmias and conduction defects.

**Figure 9:**
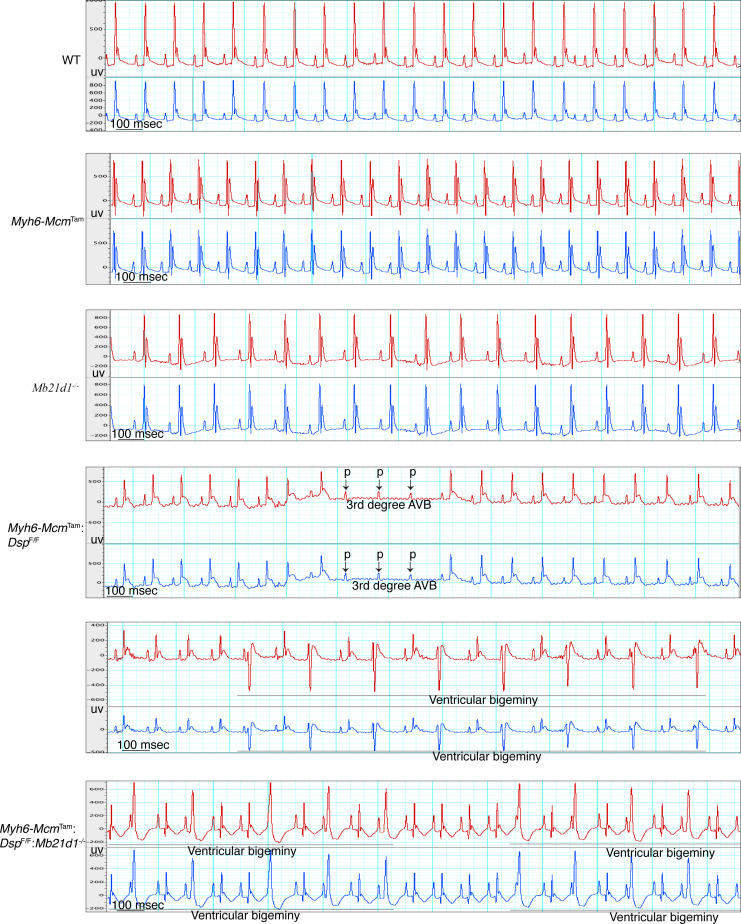
Electrocardiographic phenotype. Two-channel surface rhythm recordings in mice in the experimental groups. Normal sinus rhythm was recorded in the WT, *Myh6-Mcm*^Tam^, and *Mb21d1* mice. The *Myh6-Mcm*^Tam^ *Dsp*^fl/fl^ and *Myh6-Mcm*^Tam^ *Dsp*^fl/fl^ *Mb21d1* mice showed cardiac arrhythmias, including ventricular arrhythmias and atrioventricular blocks. There were no differences in the prevalence of arrhythmias between *Myh6-Mcm*^Tam^ *Dsp*^fl/fl^ and *Myh6-Mcm*^Tam^ *Dsp*^fl/fl^ *Mb21d1* mice. Selected ventricular arrhythmias and atrioventricular blocks (AVB) are depicted on the electrocardiograms. uv, microvolt.

Cardiac rhythm was monitored by recording the 2-channel electrocardiogram for 1 hour in each mouse. The WT, *Myh6-Mcm*^Tam^, and *Mb21d1*^–/–^ mice had normal sinus rhythm and showed no cardiac arrhythmias except for occasional premature atrial contractions (Figure 9 and Supplemental Table 1; supplemental material available online with this article; https://doi.org/10.1172/jci.insight.192283DS1). In contrast, the *Myh6-Mcm*^Tam^ *Dsp*^fl/fl^ and *Myh6-Mcm*^Tam^ *Dsp*^fl/fl^ *Mb21d1*^–/–^ showed frequent premature ventricular contractions, occasional second- and third-degree atrioventricular blocks, and runs of ventricular bigeminy and nonsustained ventricular tachycardia (Figure 9 and Supplemental Table 1). There were no differences in the prevalence of ventricular arrhythmias and atrioventricular conduction defects between the *Myh6-Mcm*^Tam^ *Dsp*^fl/fl^ and *Myh6-Mcm*^Tam^ *Dsp*^fl/fl^ *Mb21d1*^–/–^ mice.

### Myocardial cell death.

**Figure 10:**
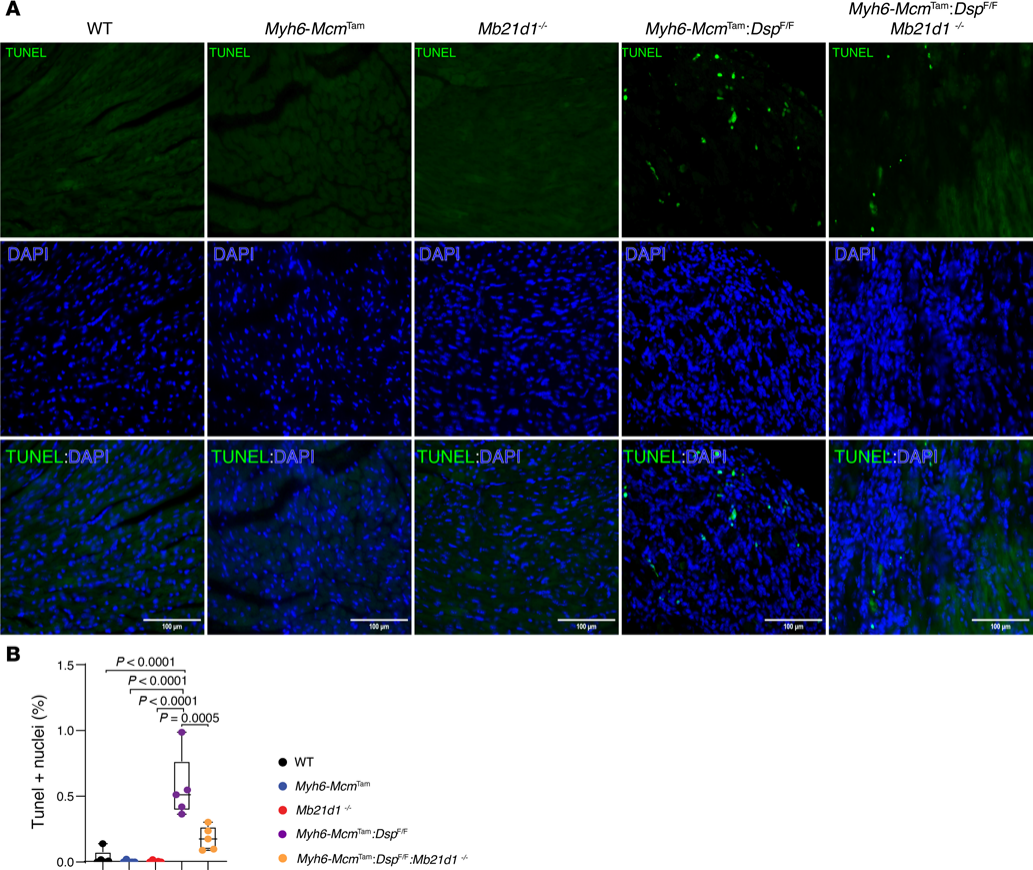
Effects of deletion of *Mb21d1* gene on myocardial apoptosis. (**A**) TUNEL-stained low-magnification myocardial sections along with the DAPI-stained sections and the overlay in the control and experimental groups. The TUNEL-stained nuclei are identified as green. TUNEL and DAPI costained nuclei are considered apoptotic cells. (**B**) Dot blots show the percentage of the apoptotic nuclei in the control and experimental groups. Each dot represents the mean value of the TUNEL-positive cells in each mouse (*N* = 4 to 5 mice per genotype). The differences were compared by ANOVA followed by pairwise comparison by the Bonferroni method. Only *P* values < 0.05 are shown.

**Figure 11:**
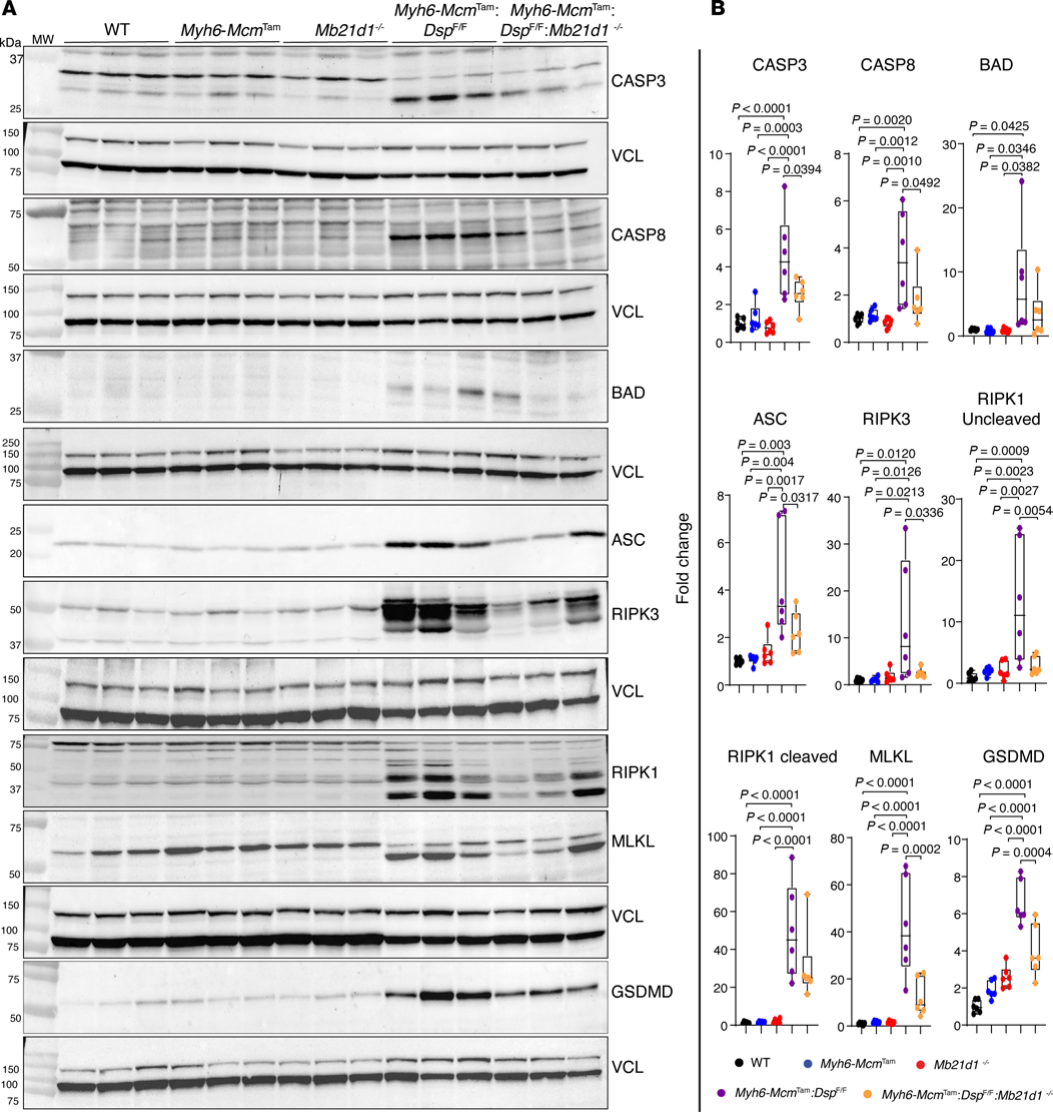
Effects of deletion of *Mb21d1* gene on protein markers of cell death programs. (**A**) Immunoblots showing protein levels of selected cell death markers composed of representatives of the PANoptosis pathways (apoptosis, necroptosis, and pyroptosis). (**B**) Dot blots showing fold change in the expression levels of the selected proteins compared with the corresponding protein in the WT mice. Data are shown as dot plots and 95% CI and compared using ANOVA followed by Bonferroni’s pairwise comparison (*N* = 5 to 6 mice per genotype).

Because PANoptosis is a prominent phenotype in the *Myh6-Mcm*^Tam^ *Dsp*^fl/fl^ mice, the effect of deletion of the *Mb21d1* gene on cell death markers was analyzed by complementary methods (8). The TUNEL assay showed a 17-fold increase in the number of nuclei that stained positive for the TUNEL in the *Myh6-Mcm*^Tam^ *Dsp*^fl/fl^ mouse heart as compared with the WT mouse heart, whereas there were no differences in the number of the nuclei stained for TUNEL among the control groups, namely WT, *Myh6-Mcm*^Tam^, and *Mb21d1*^–/–^ mice (Figure 10, A and B). Deletion of the *Mb21d1* gene reduced the number of TUNEL-stained nuclei by more than 3-fold in *Myh6-Mcm*^Tam^ *Dsp*^fl/fl^ *Mb21d1*^–/–^ as compared with *Myh6-Mcm*^Tam^ *Dsp*^fl/fl^ mouse hearts (Figure 10, A and B). To further analyze the effects of the genetic blockade of CGAS, expression levels of selected protein markers of PANoptosis were analyzed by immunoblotting in the cardiac protein extracts. Expression levels of 8 proteins involved in PANoptosis, namely CASP3, CASP8, BAD, ASC, RIPK3, RIPK1, MLKL, and GSDMD, were increased in *Myh6-Mcm*^Tam^ *Dsp*^fl/fl^ mice as compared with the control groups (Figure 11, A and B). Deletion of the *Mb21d1* gene attenuated expression levels of the cell death proteins in the *Myh6-Mcm*^Tam^ *Mb21d1*^–/–^ hearts (Figure 11, A and B).

**Figure 12:**
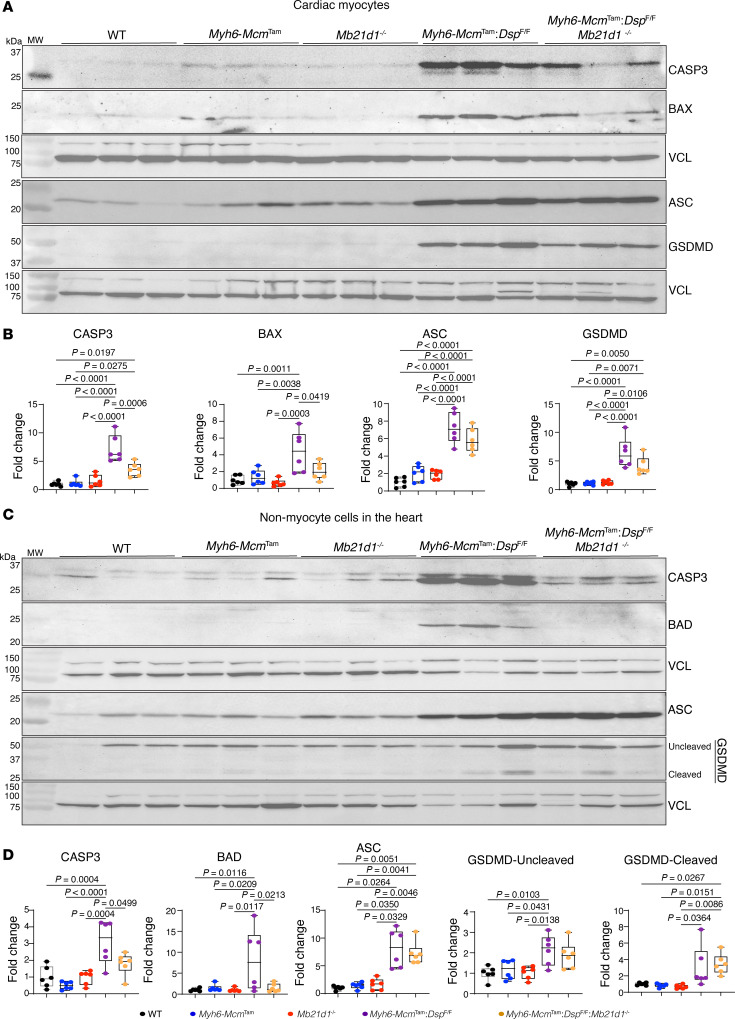
Effects of deletion of *Mb21d1* gene on selected cell death proteins in myocytes and nonmyocyte cells in the heart. (**A**) Immunoblots showing protein levels of selected cell death protein markers in cardiomyocytes isolated from mice in the control and experimental groups. (**B**) Dot blots showing fold change (FC) in the expression levels of the selected proteins in cardiomyocytes representing the blots shown in **A**. The level of the protein of interest in each group is compared with that in the WT mice and presented as FC. The differences among the groups were compared by ANOVA, followed by pairwise comparison (*N* = 6 mice per genotype). (**C**) Immunoblots showing protein levels of selected cell death protein markers in nonmyocyte cells in the heart in the control and experimental groups. (**D**) Corresponding quantitative data of protein levels shown in **C**. Data are shown as dot plots and 95% CI and compared using ANOVA followed by Bonferroni’s pairwise comparison (*N* = 6 per group). Only *P* values < 0.05 are depicted on the graphs.

To determine whether the cell death programs were activated in cardiomyocytes or nonmyocyte cells in the *Myh6-Mcm*^Tam^ *Dsp*^fl/fl^ mouse heart, the expression levels of selected cell death protein markers were analyzed by immunoblotting in isolated myocytes and nonmyocyte cells in the experimental groups. Expression levels of CASP3, ASC, and GSDMD were increased in both cardiomyocytes and nonmyocyte cells in the *Myh6-Mcm*^Tam^ *Dsp*^fl/fl^ mouse heart, whereas BAX was increased in cardiomyocytes and BAD in noncardiomyocytes (Figure 12). Deletion of the *Mb21d1* gene partially attenuated the expression levels of CASP3 and BAX in cardiomyocytes, whereas levels of ASC and GSDMD were unchanged (Figure 12). Likewise, the expression levels of CASP3 and BAD were attenuated in nonmyocyte cells upon systemic deletion of the *Mb21d1* gene, but those of ASC and GSDMD were unchanged (Figure 12).

### Myocardial fibrosis.

**Figure 13:**
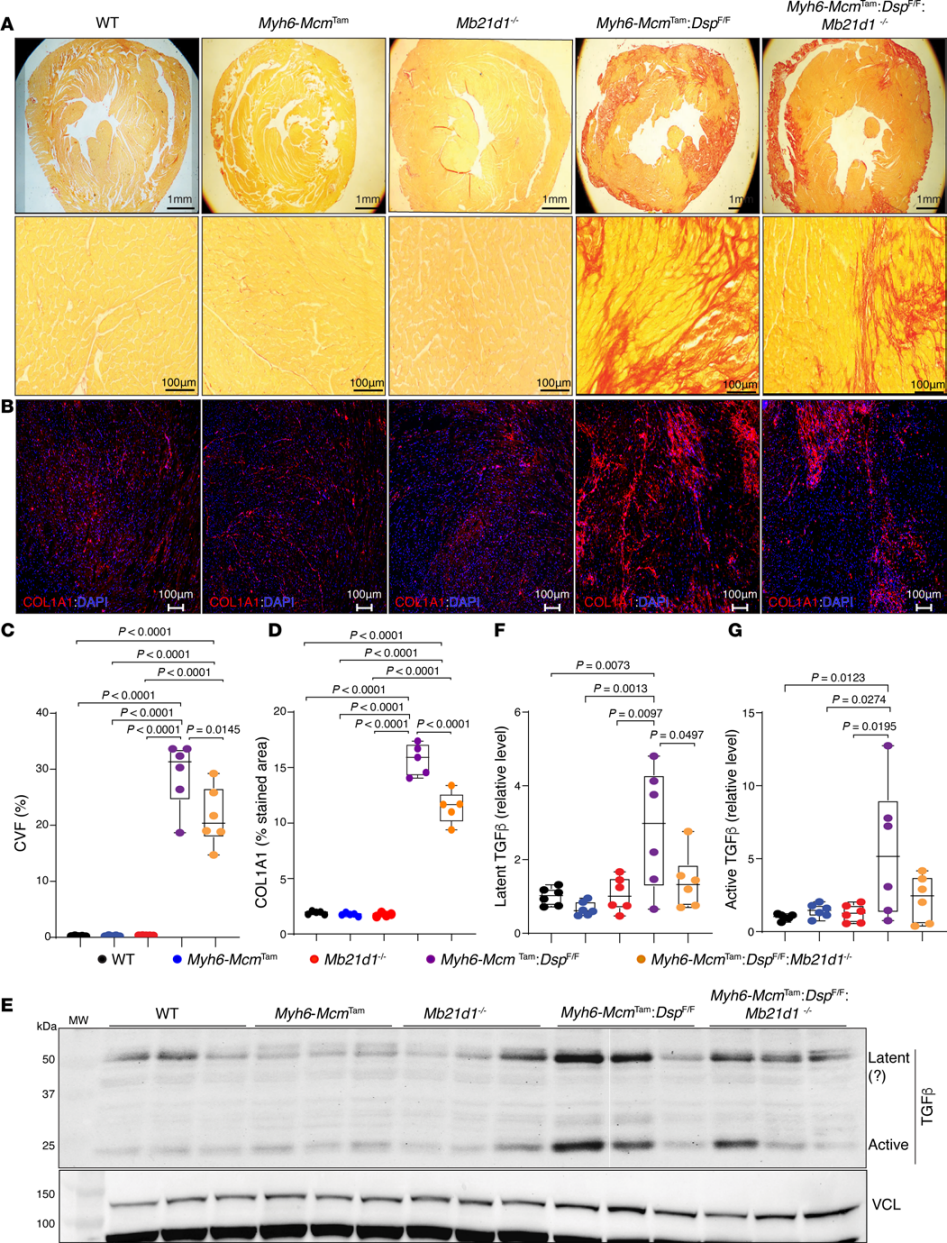
Effects of deletion of *Mb21d1* gene on myocardial fibrosis. (**A**) Picrosirius-stained thin myocardial sections in the experimental groups. Lower (the upper row) and higher (middle row) magnification sections are shown. The red color indicates fibrosis. (**B**) IF thin myocardial sections stained with an anti-COL1A1 antibody. The red areas are COL1A1-stained areas representing myocardial fibrosis. (**C**) Dot blots showing quantitative CVF calculated as the percentage of myocardium stained positive for Picrosirius red. Approximately 10 to 12 fields (20× original magnification) per section and 6 to 10 sections per heart were quantified. Each dot represents the mean value of CVF in 1 mouse. The mean values were compared by ANOVA and the differences between the 2 groups by pairwise Tukey’s test. Only *P* values < 0.05 are shown. (**D**) Dot blots showing quantitative COL1A1-stained areas (red), which were calculated and analyzed as described in **C**. (**E**) Immunoblots represent the expression of latent (likely) and active TGF-β in the control and experimental groups. Expression of VCL protein is used as a control for loading conditions. (**F**) Dot plots represent quantitative levels of what seems to be latent TGF-β protein levels in the cardiac protein extracts in the experimental groups. Data were compared as described in **C**. (**G**) Dot plots represent quantitative levels of the active mature TGF-β protein in the experimental groups. Data were compared as described in **C**.

Given that DSP cardiomyopathy, including the *Myh6-Mcm*^Tam^ *Dsp*^fl/fl^ mice, exhibit severe myocardial fibrosis, likely secondary to extensive cell death, the effects of deletion of the *Mb21d1* gene on myocardial fibrosis were determined by measuring the collagen volume fraction (CVF) and immunostaining for collagen 1 (COL1A1) in the myocardium of the control and experimental mice (4, 8). The CVF comprised about 30.0% of the myocardium in the *Myh6-Mcm*^Tam^ *Dsp*^fl/fl^, whereas it was less than 1% in the WT, *Myh6-Mcm*^Tam^, and *Mb21d1*^–/–^ mice, as also observed before (Figure 13, A and C). Deletion of the *Mb21d1* gene attenuated CVF by approximately 25%, and CVF was reduced to 21.6% ± 5.3% of the myocardium in the *Myh6-Mcm*^Tam^ *Dsp*^fl/fl^ *Mb21d1*^–/–^ mice (Figure 13, A and C). Likewise, the COL1A1-stained area encompassed less than 2% of the myocardium in the WT, *Myh6-Mcm*^Tam^, and *Mb21d1*^–/–^ (control) mice, whereas it was increased to 15.7% ± 1.4% of the myocardium in the *Myh6-Mcm*^Tam^ *Dsp*^fl/fl^ (Figure 13, B and D). Deletion of the *Mb21d1* gene attenuated the COL1A1-stained area by 27% (Figure 13, B and D). Further supporting the findings, the expression level of TGF-β, a major pro-fibrotic trophic and mitotic factor, was analyzed by immunoblotting. Levels of the latent and active TGF-β, whose identities were discerned based on their migration patterns on the PAGEs that correspond to their predicted molecular weights, were increased by about 5- to 9-fold in the *Myh6-Mcm*^Tam^ *Dsp*^fl/fl^ as compared with the WT mouse heart (Figure 13, E–G). Deletion of the *Mb21d1* gene attenuated the levels of the presumably latent and active TGF-β in the DSP-deficient (*Myh6-Mcm*^Tam^ *Dsp*^fl/fl^ *Mb21d1*^–/–^) mouse heart (Figure 13, E–G).

## Discussion

DSP cardiomyopathy is an intriguing form of cardiomyopathy whose phenotype is more consistent with the activation of the pathways involved in inflammation, fibrosis, and cell death (4, 8). The findings of the present study show that cytosolic nDNA and mtDNA are increased and the DDR pathways are activated and pathogenic in a mouse model of DSP cardiomyopathy. The deletion of the *Mb21d1* gene, encoding CGAS, a key component of the CDSP pathway, imparts salubrious phenotypic effects. The findings provide a mechanism that explains part of the enigmatic phenotype of DSP cardiomyopathy. The underpinning mechanism of the activation of the DDR pathways involves the release of the nDNA and mtDNA to the cytosol, likely secondary to mechanical, metabolic, and oxidative stress that increases DNA adducts (14). The latter increases transcriptional stress (and replication stress in replicating cells) by impairing the processivity of the RNA polymerase II (RNAPII) complex at the transcription bubble. Consequently, the exquisite coordination between RNAPII and topoisomerases, whose function is to release the torsional stress generated during nascent RNA synthesis by cutting the DNA strands, i.e., generation of DSBs, is lost. The net effect is an increased prevalence of unrepaired DSBs in the nucleus, which activates the DDR pathways. The DDR pathways broadly comprise the repair, ATM/TP53, and CDSP pathways. The repair pathway utilizes NAD to PARylate and recruits the repair proteins to the DNA damage sites (32, 33). The ATM/TP53 pathway imposes cell cycle arrest and cell death (34). The CDSP pathway, which is activated upon sensing the nDNA or mtDNA by CGAS and other CDSPs, provokes the expression of the pro-inflammatory, pro-fibrotic, and pro-death programs through the IRF3 and the NF-κB transcriptional regulators (22). Consequently, genetic blockade of the CDSP pathway upon deletion of the *Mb21d1* gene, encoding CGAS, imparted considerable salubrious phenotypic effects by prolonging survival, improving cardiac function, reducing cell death, and attenuating myocardial fibrosis, which are the cardinal features of DSP cardiomyopathy. The findings provide a mechanism in the pathogenesis of DSP cardiomyopathy and point to the potential therapeutic utility of targeting the CDSP pathways.

The findings of activation of the DDR pathways, comprising the CGAS/STING1 and ATM/TP53 pathways, as well as the salubrious effects of the genetic blockade of the CDSP pathway, were confirmed by complementary methods and were consistent across the phenotypes. Although both the ATM/TP53 and the CDSP components of the DDR pathways were activated in the cardiomyocytes isolated from the *Myh6-Mcm*^Tam^ *Dsp*^fl/fl^ mice, only the CDSP pathway was targeted by deleting the *Mb21d1* gene, which led to partial blockade of the CGAS/STING1 downstream effectors. Despite genetic blockade of the CGAS only and leaving the activated ATM/TP53 pathway uninterrupted, the effect size of the benefit was remarkable for survival, cardiac function, and cell death. The attenuation of myocardial fibrosis, however, was less remarkable, and fibrosis remained considerably increased despite the effective deletion of the *Mb21d1* gene. The latter may reflect the expression of SASP, which is known to mediate fibrosis, through the activated ATM/TP53 pathway (35). Notwithstanding the salubrious effects, the incomplete phenotypic rescue is expected and is in accord with the multiple mechanisms that are involved in the pathogenesis of DSP cardiomyopathy, including the involvement of the canonical WNT and Hippo pathways among others (9, 36, 37). The findings are also in agreement with our previous data on the activation of the DDR pathways in other forms of cardiomyopathy, including in mouse models and humans with lamin A/C (LMNA) cardiomyopathy (24). Collectively, the findings provide the impetus for pharmacological interventions to target components of the DDR pathways for therapeutic gains in cardiomyopathies (15, 17, 18, 24, 37).

Increased cytosolic self-DNA provides a mechanism for the activation of the CDSP pathway in *Myh6-Mcm*^Tam^ *Dsp*^fl/fl^ cardiomyocytes, as CGAS upon sensing cytosolic DNA generates cGAMP, which binds to and activates STING1 (38, 39). Activated STING1 recruits TBK1, which targets IRF3 for phosphorylation, dimerization, and nuclear localization, where it induces the expression of the ISGs (21, 40, 41). However, despite the evidence of increased expression of the IRF3 target genes in the *Myh6-Mcm*^Tam^ *Dsp*^fl/fl^ cardiomyocytes, the levels of p-IRF3 both at Ser386, which is located at the activation site, and at Ser396, which is located at the autoinhibitory site, were reduced in the *Myh6-Mcm*^Tam^ *Dsp*^fl/fl^ cardiomyocytes (29). While this finding was unanticipated, it is consistent with the previous data in cardiomyocytes isolated from a mouse model of LMNA cardiomyopathy, which also showed reduced levels of p-IRF3 (Ser396), and may reflect cell type–specific regulation and the differences among the species (24, 29). Finally, consistent with the role of TBK1 in the activation of NF-κB1, the NF-κB1 target genes were also enriched in the *Myh6-Mcm*^Tam^ *Dsp*^fl/fl^ cardiomyocytes (30, 31).

The mechanism(s) responsible for the release of the nDNA and mtDNA in cardiomyocytes in DSP cardiomyopathy remains to be determined; suffice it to state that internal DNA stressors, including metabolic and oxidative damages, are increased in cardiomyopathies that increase DNA adducts and cause transcription (and to lesser degree replication stress) in cardiomyocytes (42, 43).Accordingly, levels of 8-hydroxyl-2′-deoxyguanosine, a common marker of oxidative DNA damage, and malondialdehyde (MDA) and 4-hydroxy-2-nonenal, which are markers of lipid peroxidation, are increased in the heart in cardiomyopathies (42, 43). Metabolic by-products, including MDA, are known to form DNA cross-links, which impose considerable replication and transcription stress, which could lead to DNA breaks (44, 45). In agreement with increased DNA metabolic stress, the prevalence of genome-wide DSBs is increased in cardiomyocytes in a mouse model of LMNA cardiomyopathy, and increased DSBs are coupled to transcription (46).

Activation of the ATM/TP53 pathway, including increased expression of H2AFX, indicates the accumulation of DSBs in the nuclear genome (34). Whether the increase in markers of DSBs in DSP cardiomyopathy is a consequence of increased transcriptional stress resulting from the secondary modification of the genomic DNA or is a consequence of defective repaired mechanisms caused by posttranslational modifications of the repair enzymes and/or depletion of NAD^+^ in the pathological states remains to be determined. Likewise, the identification and characterization of the genome-wide DSBs in DSP cardiomyopathy remain unexplored.

Deletion of the *Mb21d1* gene had no effect on the prevalence of cardiac arrhythmias or conduction defects in the cardiomyocyte-specific DSP-null mice. We speculate that prolonged survival in the *Myh6-Mcm*^Tam^ *Dsp*^fl/fl^ *Mb21d1*^–/–^ mice was likely because of the beneficial effect of partial inhibition of the CDSP pathway on cardiac function and unlikely because of a reduced burden of cardiac arrhythmias. Nevertheless, the cause of death in the *Myh6-Mcm*^Tam^ *Dsp*^fl/fl^ mice and the reasons for an improved survival rate in the *Myh6-Mcm*^Tam^ *Dsp*^fl/fl^ *Mb21d1*^–/–^ cannot be ascertained accurately.

A limitation of the study is the systemic deletion of *Mb21d1* as opposed to its myocyte-specific deletion. The approach limits the ability to discern the contribution of activation of the CDSP pathway in cardiomyocytes to the pathogenesis of DSP cardiomyopathy. We note that the expression levels of several paracrine factors, including those that are targets of IRF3 and NF-κB, were increased in cardiomyocytes in the *Myh6-Mcm*^Tam^ *Dsp*^fl/fl^ mice and were attenuated upon deletion of the *Mb21d1* gene along with the attenuation of histological, functional, and clinical phenotypes. The findings imply that the activation of the CDSP pathway in cardiomyocytes contributes to the pathogenesis of DSP cardiomyopathy. It also merits noting the expression levels of the selected paracrine factors were increased in nonmyocyte cells in the heart. Whether the increased expression levels of the paracrine factors in nonmyocyte cells, which include the immune cells, were secondary to the secretion of paracrine factors from DSP-deficient myocytes or due to other forms of cell stress was not determined. Despite the systemic deletion of the *Mb21d1* gene, however, the expression levels of several paracrine factors remained partially elevated in the nonmyocyte cells in the heart. The findings suggest that continued expression of the paracrine factors from nonmyocyte cells in the heart also may contribute to the phenotypic expression of DSP cardiomyopathy and perhaps in part is responsible for the residual phenotype in DSP cardiomyopathy after deletion of the *Mb21d1* gene.

Increased expression levels of selected paracrine factors and cell death proteins in isolated cardiomyocytes are also subject to the impurity of the isolation and the potential compounding effects of the nonmyocyte cells, particularly immune cells, and fibroblasts that might be attached to the isolated myocytes or are simply passenger during isolation. Overall, the findings suggest broad attenuation of inflammation, fibrosis, and cell death in DSP cardiomyopathy.

In conclusion, cytosolic self-DNA (nDNA and mtDNA) is increased in cardiomyocytes in DSP cardiomyopathy and activates the CDSP (CGAS/STING1) pathway, which induces the expression of pro-inflammatory genes involved in cardiac dysfunction, cell death, and fibrosis. The findings provide a mechanism for the unusual phenotype of inflammation, fibrosis, and excess cell death in DSP cardiomyopathy. Moreover, the findings establish the salubrious effects of the blockade of the CDSP pathway, upon genetic deletion of the *Mb21d1* gene, in a mouse model of DSP cardiomyopathy. In conjunction with the previous data, these findings set the stage for pharmacological interventions targeting the CDSP pathway to prevent, attenuate, or reverse the phenotype in DSP cardiomyopathy. Given the ubiquitous nature of the metabolic derangement and, hence, increased metabolic stress on the nuclear and mitochondrial genomes in pathological states, the findings would be expected to impart broader implications.

## Methods

### Sex as a biological variable.

An equal number of age-matched male and female mice were included in all studies, and the data were analyzed with sex as a biological variable to discern the putative effect of sex on the phenotypic data.

### Anesthesia and euthanasia.

Anesthesia was induced in mice with inhalation of 3% and was maintained with 0.5% isoflurane. Mice were euthanized upon inhalation of 100% CO_2_ and cervical dislocation.

### Strain and source of mice.

Mice were of C57BL/6J background. All male and female mice have been bred in our facility for at least 2 years and have been backcrossed over a dozen times.

### WT mice.

Age- and sex-matched WT mice were included as controls. The genetic background of the WT and the experimental groups (see below) was the C57BL/6J.

### Myh6-Mcm^Tam^ mice.

The mice denote myocyte-specific Cre, which is activated upon treatment with tamoxifen. The mice were also included as controls.

### Myh6-Mcm^Tam^ Dsp^fl/fl^ mice.

The *Myh6-Mcm*^Tam^ *Dsp*^fl/fl^ mice carry homozygous, myocyte-specific, tamoxifen-inducible, Cre recombinase–mediated deletion of the *Dsp* gene. The *Dsp* gene was deleted in the postnatal cardiomyocytes upon intraperitoneal injection of tamoxifen (30 mg/kg/d) for 5 days starting on postnatal day 14, as published (8, 13).

### Mb21d1^–/–^ mice.

The *Mb21d1*-knockout mice [B6(C)-Cgas^tm1d(EUCOMM)Hmgu^/J, RRID:IMSR_JAX:026554] carry the deletion of exon 2 of the *Mb21d1* gene, which encodes the catalytic subunit of the CGAS protein (28, 47). The mice are susceptible to murine gamma-herpesvirus 68, vaccinia, and West Nile viruses but do not show a discernible cardiac phenotype (24, 28).

### Myh6-Mcm^Tam^ Dsp^fl/fl^ Mb21d1^–/–^ mice.

Mice with the compound genotypes of *Myh6-Mcm*^Tam^ *Dsp*^fl/fl^ *Mb21d1*^–/–^ represent systemic deletion of the *Mb21d1* gene in the background of tamoxifen-inducible Cre recombinase–mediated homozygous deletion of the *Dsp* gene. The mice were generated by crossing the *Myh6-Mcm*^Tam^ *Dsp*^fl/fl^ and *Mb21d1*^–/–^ mice and treating the offspring with tamoxifen at the postnatal day 14 for 5 consecutive days, as in the *Myh6-Mcm*^Tam^ *Dsp*^fl/fl^ mice.

### Genotyping.

Genotyping was performed by PCR of genomic DNA isolated from the mouse tails, as published (9, 24, 48). The list of oligonucleotide primers used for the genotyping is provided in Supplemental Table 2.

### Survival analysis.

Kaplan-Meier survival plots were constructed for mice with each genotype, and survival rates were compared using GraphPad Prism 10 software (https://www.graphpad.com/).

### Echocardiography.

Cardiac size and function in mice were assessed by 2D and M-mode echocardiography upon induction of light anesthesia with 1% isoflurane inhalation, as published (8, 13, 17). The LV anterior and posterior wall thicknesses, LVEDD, and LVESD were measured from the 2D-guided M-mode images at the level of papillary muscles by the leading-edge method. The LVFS was calculated from the above measurements. The LVEDD and left ventricular mass were corrected to the body weight (49). Echocardiography was performed on 12 to 15 mice, an equal number of male and female mice, per genotype.

### Cardiac rhythm monitoring.

A 2-channel surface electrocardiogram was recorded for about an hour in each mouse using a Power Lab 4/30 data acquisition system while the mouse was kept under light anesthesia, as published (35, 36). The rhythm was analyzed for the presence of cardiac conduction defects and arrhythmias using Lab Chart 7 software (ADInstruments).

### Isolation of cardiomyocyte and nonmyocyte cells in the heart.

Cardiomyocytes were isolated from 4-week-old mice by the collagenase perfusion method, as published (13, 50). In brief, the heart was excised, the aorta was cannulated, and the heart was mounted onto a Langendorff perfusion system. The heart was perfused retrogradely with a buffer that contained 2.4 mg/mL of type 2 collagenase (Worthington catalog LS004176) at a flow rate of 4 mL/min. Upon complete softening of the heart muscle, the ventricles were excised from the atria and minced into small pieces in a buffer that contained 10% calf serum, 12.5 μM CaCl_2_, and 2 mM ATP. The cells then passed through a 100 μm cell strainer, and the myocytes were precipitated by centrifugation at 20*g* for a few minutes. The isolated cardiomyocytes were reintroduced to CaCl_2_ in a stepwise manner by gradually increasing the calcium concentration from 100 μM to 900 μM. The isolated cells were suspended in a RIPA buffer for protein extraction or QIAzol reagent (QIAGEN catalog 79306) for RNA extraction.

The nonmyocyte cells in the heart were isolated using a Langendorff isolated heart perfusion system as above (8, 13, 16, 50). In brief, upon complete dissociation of the tissue and stopping collagenase activity, the cell suspension was passed through a 100 μm mesh cell strainer to remove the debris. The cells were centrifuged at 300*g* for 4 minutes at room temperature to precipitate the myocytes. The supernatant, which contains the nonmyocyte cells, were transferred to a clean tube and spun down at 1,300*g* for 5 minutes at room temperature to pellet the cells. The pellet was washed with PBS before use.

### RNA-Seq.

The strand-specific 75 bp paired-end reads RNA-Seq data in the WT, *Myh6-Mcm*^Tam^, and *Myh6-Mcm*^Tam^ *Dsp*^fl/fl^ myocytes were used to analyze specific biological pathways and transcriptional regulators of gene expression (8, 13).

### Detection of cytosolic DNA by confocal microscopy.

Cytosolic nDNA and mtDNA in the isolated cardiomyocytes were detected by IF staining for DNA and selected protein markers. In brief, to detect nDNA, cardiomyocytes were isolated from 4-week-old WT, *Myh6-Mcm*^Tam^, and *Myh6-Mcm*^Tam^ *Dsp*^fl/fl^ mice and plated on laminin-coated coverslips in myocyte-plating medium in a 2% CO_2_ incubator at 37°C for 4 hours. Then, the cells were washed with 1× PBS 3 times, fixed with ice-cold 100% methanol for 15 minutes at –20°C, and incubated in a blocking buffer containing 10% goat serum, 1% bovine serum albumin (BSA), and 0.1% Triton X-100 in 1× PBS for an hour. To detect cytoplasmic nDNA, the isolated myocytes were stained with SYBR Green (Invitrogen, S7563, 1:10,000) to mark the DNA and antibodies against p-H2AFX, TP53BP1, or H3K9ac. The first 2 proteins are the markers of DSBs, and the third is an epigenetic marker associated with the active promoters, where DSBs are enriched (46, 51–53). Cells were incubated with primary antibodies overnight at 4°C and then incubated with the corresponding secondary antibodies conjugated to fluorophores for 1 hour at room temperature. The coverslips were mounted with the cells facing toward the microscope slide with an antifade mounting medium (Invitrogen, catalog P36970). The slides were examined using a confocal microscope (Leica TCS SP5) at 63× original magnification. An average of approximately 3,000 cells per heart (*N* = 6 mice per genotype, both males and females) were analyzed for the presence of cytosolic DNA. The antibodies used and their concentrations are listed in Supplemental Table 2.

To detect cytosolic mtDNA (outside of the mitochondria), cardiomyocytes were fixed with 4% paraformaldehyde for 20 minutes, permeabilized with 0.1% Triton X-100 for 30 minutes, and blocked with 10% goat serum, 1% BSA in 1× PBS, for an hour at room temperature. The cells were stained with SYBR Green to detect DNA and an antibody against ATP5F1A to identify the mitochondria. The corresponding secondary antibody was added, and the cells were examined under a confocal microscope (Nikon AX-R Confocal Laser Microscope System) at 63× original magnification. At least 5 animals per genotype (both males and females) were examined.

### Detection of cytosolic DNA by PCR.

To further corroborate the IF findings, cardiomyocyte cytosolic DNA was isolated from 4-week-old WT and *Myh6-Mcm*^Tam^ *Dsp*^fl/fl^ mice, as published (13). The isolated myocytes from each mouse heart were divided into 2 even fractions. One fraction was used to extract the cytosolic component of the myocytes, and the other half to extract the whole myocyte DNA, the latter serving as a control for the total DNA. Cardiomyocyte subcellular fractions were separated using a Mitochondria/Cytosol Fractionation Kit (Abcam, catalog ab65320). For each sample, both cytosolic components and whole myocytes were incubated with 0.2 mg/mL proteinase K at 55°C for 3 hours, followed by RNase A (100 μg/mL) incubation at 37°C for 30 minutes. The cytosolic DNA was extracted with phenol/chloroform/isoamyl alcohol (25:24:1) and precipitated in 0.1 volume of sodium acetate (3M) and 1 volume of isopropanol by centrifugation at 15,000*g* for 30 minutes at 4°C. The supernatant was removed, and the DNA pellet was washed with 70% ethanol, dried at room temperature, and resuspended in 50 μL Tris-EDTA buffer. The DNA concentration was measured using a NanoDrop instrument (Thermo Fisher Scientific), and the samples were diluted in double-distilled water (ddH_2_O) at a final concentration of 5 ng/μL.

The relative copy number of the cytosolic and total nDNA and mtDNA was determined by PCR, which was performed in triplicates for each sample. Each reaction contained 5 ng of DNA, 1.5 μL of ddH_2_O, 2.5 μL of SYBR Green Mix, and 0.5 μL of forward and reverse primer mix. The samples were denatured at 95°C for 5 minutes and then subjected to 45 cycles of amplification, which comprised denaturation at 95°C for 10 seconds, annealing at 60°C for 10 seconds, and extension at 72°C for 20 seconds. The ratio of cytosolic DNA to total myocyte DNA for each gene was calculated as follows: cytosolic/total myocyte DNA (%) = 2^[Ct(total myocyte DNA) – Ct(cytosolic DNA)] × 100%. PCR was performed in 5 to 9 independent samples per genotype. The primer sequences used are listed in Supplemental Table 2.

### RT-PCR.

Transcript levels of the candidate genes of interest were analyzed by RT-PCR upon extraction of the total cardiomyocyte RNA using a miRNeasy Mini Kit (QIAGEN, catalog 217006) and following digestion of the DNA with DNase I (QIAGEN, catalog 79254). Approximately, ~1 μg of total RNA was used in the reverse transcription reaction along with random primers using a high-capacity cDNA synthesis kit (Applied Biosystems catalog 4368814). The SYBR Green probes were used, and each reaction was performed in triplicates. Transcript levels of *Vcl* were used for normalization of the test transcript levels. The ΔΔCT method was used to calculate the differences in the transcript levels. The data were presented as fold changes relative to the corresponding levels in the WT myocyte extracts. RT-PCR was performed in 5 to 10 independent cardiomyocyte RNA extracts per genotype. The primers used in the study are listed in Supplemental Table 2.

### Immunoblotting.

Immunoblotting was performed on ventricular tissue or cardiomyocyte protein extracts, as published (15, 54). In brief, the tissues or cells were homogenized and dissolved in a RIPA buffer, which contained 0.5% SDS and inhibitors of proteases and phosphatases (Roche, catalog 04693159001 and catalog 04906837001, respectively). The lysates were sonicated using a Bioruptor Pico (Diagenode), and the proteins were precipitated by centrifugation at 15,493*g*. Approximately, 30 to 100 μg aliquots of protein extracts were subjected to electrophoresis in an SDS-PAGE and transferred to a nitrocellulose membrane. The membranes were incubated with the primary antibodies against the proteins of interest overnight in a cold room and then probed with the corresponding secondary antibodies. The ECL Western blotting detection kit (Amersham catalog RPN2106) was used to detect the signal. The images were collected using the LI-COR Odyssey imaging system. Immunoblotting was performed in 5 to 9 independent cardiomyocyte protein extracts per genotype.

### TUNEL assay.

The TUNEL assay was used to detect and calculate the percentage of cells undergoing apoptosis, as published (8, 13, 50). The in-situ cell death detection fluorescein kit (Roche catalog 11684795910) was used to detect the number of nuclei, identified by DAPI staining, that were stained positively for the TUNEL assay. Approximately, 20,000 cells per heart and 5 mice per genotype were examined, and the percentage of the TUNEL-positive cells was calculated in each group.

### CVF.

Myocardial fibrosis was assessed by staining thin myocardial sections with Picrosirius red and calculating the percentage area stained for collagen, as published (8, 13, 15). CVF was calculated in 6 mice per genotype.

### IF.

To complement the CVF findings, thin myocardial sections were stained with a COL1A1 antibody, and the percentage areas stained positive for collagen 1 were calculated as published (*N* = 5 per genotype) (55).

### Statistics.

Each set of data was analyzed for a Gaussian distribution using the Shapiro-Wilk normality test. Data that followed a normal distribution pattern were compared among the groups by 1-way ANOVA followed by pairwise comparisons by the Bonferroni or Tukey method. Data that did not follow a Gaussian distribution were compared by the Kruskal-Wallis test. The categorical data were compared by the Fisher exact or the χ^2^ test. The survival rates in the Kaplan-Meier survival plots were compared using the log-rank test. The RT-PCR data were compared between the groups using the Mann-Whitney *U* test. In figures, box plots show the interquartile range, median (line), and minimum and maximum (whiskers) or mean and 95% confidence interval as specified. No sample was excluded from the analysis. Statistical analyses were performed using GraphPad Prism 9 or STAT IC, 15.1. A *P* value less than 0.05 was considered significant.

The dysregulation of the specific biological pathways was analyzed by GSEA (version 2.2.3), as published (8, 13). The candidate Hallmark canonical pathways were analyzed using the Molecular Signature Database 3.0 and NES.

### Study approval.

The Institutional Care and Use Committee of the University of Texas Health Sciences Center at Houston approved the use of mice in these studies (Protocol AWC-24-0020). All experiments in mice were performed according to the NIH *Guide for the Care and Use of Laboratory Animals* (National Academies Press, 2011).

### Data availability.

Technical data and source files are available from the corresponding author upon request without restriction. The RNA-Seq data used in this study have been deposited at NCBI GEO (GSE180972) and are publicly available without restrictions.

## Author contributions

WW performed myocyte and nonmyocyte isolation and immune staining to detect cytosolic nuclear and mitochondrial DNA as well as immunoblotting and RT-PCR. WW is listed first as the co–first author, as he completed the additional studies requested during the review process. BC generated the mice and performed phenotypic characterization, including survival analysis, echocardiography, immunoblotting, and isolation of cardiomyocytes. QDN maintained the mouse colony and helped with genotyping, myocyte isolation, immunostaining, and immunoblotting. LQL assisted with maintaining the mouse colony, genotyping, and Picrosirius red staining of the myocardial sections and immunoblotting. AB assisted with maintaining the mouse colonies and performed genotyping and the TUNEL assay. SEC assisted with maintaining the mouse colonies, genotyping, and immunoblotting. LR performed IF staining and immunoblotting, assisted with the analysis of the echocardiography data, and edited the manuscript. PG analyzed and interpreted the RNA-Seq data and edited the manuscript. AJM conceived the idea, designed the research, interpreted the findings, and wrote the manuscript.

## Supplementary Material

Supplemental data

Unedited blot and gel images

Supporting data values

## Figures and Tables

**Table 1:**
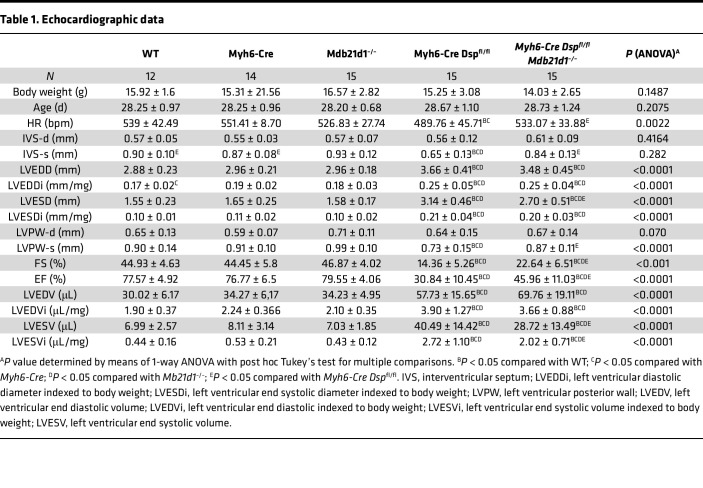
Echocardiographic data

## Version 1 07/03/2025

In-Press Preview

## Version 2 08/22/2025

Electronic publication
