# Supplementary material for: Cardiomyocyte cytosolic nuclear self-DNA contributes to the pathogenesis of desmoplakin cardiomyopathy
Source: JCI Insight. 2025 Jul 3;10(16):e192283. doi: 10.1172/jci.insight.192283 (PMC12406735; doi:10.1172/jci.insight.192283)
Supplement: Supplemental data [file jciinsight-10-192283-s075.pdf]

Online Table 1

Electrocardiographic Data

Genotype	Wild type	*Myh6-Mcm*^{Tam}	*Mb21d1*$^{-/-}$	*Myh6-Mcm*^{Tam}:*Dsp*$^{F/F}$	*Myh6-Mcm*^{Tam}:*Dsp*$^{F/F}$: *Mb21d1*$^{-/-}$	*p* (ANOVA)
Number of mice	7	8	11	19	19	NA
Heart rate (bpm)	467.3±36.6	470.8±36.1	477.6± 24.8	480.9±33.7	498.6±33.9	0.1363
P duration (msec)	14.3±2.1	14.5±1.9	14.1±1.5	13.9±2.2	14.9±1.9	0.6080
PR Interval (msec)	40.8±8.8	38.4±4.0	39.3±5.4	35.6±5.1	39.5±4.5	0.1330
QRS duration (msec)	16.6±3.8	17.3±2.9	16.1±1.0	15.6±2.5	17.4±3.6	0.4763*
2nd degree AVB (N, %)	0 (0, 0%)	0.1±0.4 (1, 13%)	0 (0, 0%)	0.6±1.5 (13, 16%)	0.3± 0.7 (5, 16%)	0.3893*
3rd degree AVB (N, %)	0 (0, 0%)	0 (0, 0%)	0 (0, 0%)	0.4±1.2 (3, 16%)	0.1±0.2 (1, 5%)	0.3201*
PACs (N, %)	1.6±3.7 (11, 29%)	0.3±0.5 (2, 25%)	5.0±3.9 (55, 73%)	1.5±3.2 (28, 42%)	1.8±5.5 (35, 26.3%)	0.0223*
PVCs (N, %)	0 (0, 0%)	0 (0, 0%)	0 (0, 0%)	137.0±556.1 (2,466, 65%)	211.2±521.7 (4,103, 79%)	0.5679
SVT (N, %)	0 (0, 0%)	0 (0, 0%)	0 (0, 0%)	0 (0, 0%)	0.2±0.7 (3, 5%)	0.6810
VT (N, %)	0 (0, 0%)	0 (0, 0%)	0 (0, 0%)	0.2±0.3 (2, 11%)	0.2±0.3 (2, 11%)	1.0000
Other rhythms	None	None	None	Isorhythmic atrial rhythm, lasting up to 360 sec. Isorhythmic ventricular rhythm lasting 30 min.	Isorhythmic atrial rhythm lasting up to 250 sec. Frequent runs of ventricular bigeminy.	NA

Abbreviations: *Myh6*: Myosin heavy chain; *Mcm*^{Tam}: MerCreMer mice injected with tamoxifen; *Mb21d1*: Gene encoding CGAS; *Dsp*$^{F/F}$: Gene encoding Dsp with floxed alleles; NA: Non-applicable; AVB: atrioventricular block; N: number per events; %: Precent of mice with the event; PACs: Premature atrial contractions; PVCs: Premature ventricular contractions; SVT: supraventricular tachycardia; VT: Ventricular tachycardia.

* By the Kruskal-Wallis because of unequal variations

Online Table 2

Oligonucleotide Primers, Antibodies

Genotyping Primers

**Gene**	**Forward sequence**	**Reverse sequence**
Cre recombinase	TCTATTGCACACAGCAATCCA	WT: GTAGGAAGTTCAAAGGATGC<br>Mutant: CCAGCATTGTGAGAACAAGG
Mb21d1(Ex2)	ATATTTCCCCCTGTGTTGGA	WT: GTGCCAGGTGACACAACATC<br>Mutant: CGGATGGATGAACAAACAGA
Dsp	GTTGGGCCTCTCGAATCAT	TCTTTGTCTGTTGCCATGTGA

List of antibodies

**Antibodies**	**Concentration**	**Supplier**	**Catalog number**
Anti-mouse IgG HRP linked antibody	1:3000 (IB)	Cell Signaling Technology	7076
Anti-Rabbit IgG HRP linked antibody	1:3000 (IB)	Cell Signaling Technology	7074
ASC	1:1000(IB)	Cell Signaling Technology	67824
ATM	1:1000(IB)	Cell Signaling Technology	2873S
ATP5F1A	1:100 (IF)	Proteintech	66037-1-Ig
BAD	1:1000(IB)	Cell Signaling Technology	9292
BAX	1:2000(IB)	Proteintech	50599-2-Ig
CASP3	1:1000(IB)	Cell Signaling Technology	14220
CASP8	1:1000(IB)	Cell Signaling Technology	4927
CDKN1A	1:250 (IB)	BD Pharmingen	556431
CGAS	1:1000(IB)	Cell Signaling Technology	31659
COL1A1	1:100 (IF)	Abcam	Ab21286
GAPDH	1:1000(IB)	Abcam	Ab8245
Goat anti Mouse Alexa Fluor 647	1:500 (IF)	Invitrogen	A32728
Goat anti Rabbit Alexa Fluor 594	1:1000 (IF)	Invitrogen	A11012
GSDMD	1:1000(IB)	Cell Signaling Technology	39754
H3K9ac	1:500 (IF)	Active Motif	39917
IRF3	1:1000(IB)	Cell Signaling Technology	4302

MLKL	1:1000(IB)	Cell Signaling Technology	37705
MMP9	1:1000(IB)	Proteintech	10375-2-AP
P50	1:1000(IB)	Cell Signaling Technology	13586
P52 (RELB)	1:1000(IB)	Cell Signaling Technology	4882S
P60	1:1000(IB)	Cell Signaling Technology	8242
Phospho-Histone H2A.X (Ser139)	1:100 (IF) 1:1000(IB)	Cell Signaling Technology	2577S
Phospho-IRF3-386	1:1000(IB)	Cell Signaling Technology	37829
Phospho-IRF3-396	1:1000(IB)	Cell Signaling Technology	4947
RIPK1	1:1000(IB)	Cell Signaling Technology	3493
RIPK3	1:1000(IB)	Cell Signaling Technology	15828
SFRP3	1:500(IB)	Santa Cruz	sc-514350
SPP1	1:1000(IB)	Proteintech	30200-1-AP
STING1	1:1000(IB)	Abcam	ab92605
TBK	1:1000(IB)	Cell Signaling Technology	3504
TGFβ	1:1000(IB)	Bio-Techne	mab1835
TP53	1:1000(IB)	Cell Signaling Technology	32532S
TP53BP1	1:100 (IF)	Novus	NB100-304
TUBA1A	1:1000(IB)	Cell Signaling Technology	2125S
VCL	1:10000 (IB)	Abcam	Ab129002
VIM	1:5000(IB)	Proteintech	10366-1-AP

Oligonucleotide primers used in qPCR reactions

**Name**	**Sequence**
12s	Forward: AAGGTTTGGTCCTGGCCTTA<br>Reverse: GTGGCTAGGCAAGGTGTCTT
16s	Forward: CACTGCCTGCCCAGTGA<br>Reverse: ATACCGCGGCCGTTAAA
18s	Forward: GGCTCTTCCGTGTCTACGAG<br>Reverse: CCAGCCAACGTAGAAAAGCC
Actb	Forward: CTTGTTTGCGCTTCCTGCTG<br>Reverse: ACCGGCCGCATTATTACCAT
Apob	Forward: CGTGGGCTCCAGCATTCTA<br>Reverse: TCACCAGTCATTTCTGCCTTTG
Ccl7	Forward: CCACATGCTGCTATGTCAAGA

	Reverse: ACACCGACTACTGGTGATCCT
Cd44	Forward: GGCTCTGATTCTTGCCGTCT<br>Reverse: TCCTGTCTTCCACTGTCCCA
Cd83	Forward: CGCAGCTCTCCTATGCAGTG<br>Reverse: GTGTTTTGGATCGTCAGGGAAT
Cox1	Forward: TCGGAGCCCCAGATATAGCA<br>Reverse: TTTCCGGCTAGAGGTGGGTA
Cytb	Forward: GCTTTCCACTTCATCTTACCATTTA<br>Reverse: TGTTGGGTTGTTTGATCCTG
Gapdh	Forward: GCCTCCTCCAATTCAACCCT<br>Reverse: CATAGGTCAGGATGCCCTGC
Hk2	Forward: GCCAGCCTCTCCTGATTTTAGTGT<br>Reverse: GGGAACACAAAAGACCTCTTCTGG
Irf7	Forward: GAGACTGGCTATTGGGGGAG<br>Reverse: GACCGAAATGCTTCCAGGG
Mmp9	Forward: GGACCCGAAGCGGACATTG<br>Reverse: CGTCGTCGAAATGGGCATCT
Nd1	Forward: CTAGCAGAAACAAACCGGGC<br>Reverse: CCGGCTGCGTATTCTACGTT
Nd4	Forward: AACGGATCCACAGCCGTA<br>Reverse: AGTCCTCGGGCCATGATT
Nr4a2	Forward: GTGTTCAGGCGCAGTATGG<br>Reverse: TGTATTCTCCCGAAGAGTGGTAA
Rps18	Forward: TGTGTTAGGGGACTGGTGGACA<br>Reverse: CATCACCCACTTACCCCCAAAA
Sfrp1	Forward: TACTGGCCCGAGATGCTCAA<br>Reverse: GAGGCTTCCGTGGTATTGGG
Sfrp2	Forward: CCCTTTGTAAAAATGACTTCGCAC<br>Reverse: CAGGATGATCTTGGTGTCTCTGT
Spp1	Forward: AGCAAGAAACTCTTCCAAGCAA<br>Reverse: GTGAGATTCGTCAGATTCATCCG
Tnc	Forward: TTTGCCCTCACTCCCGAAG<br>Reverse: AGGGTCATGTTTAGCCCACTC
Tnfrsf1a	Forward: GGCAGTGCATACCTGTTTTTG<br>Reverse: AACCGCAACTGGACGATGAG
Vcl	Forward: CAGCCCAGATGCTTCAGTCA<br>Reverse: CGAACCTCAGCCTCATCGAA
Vim	Forward: TGCACGATGAAGAGATCCAGG<br>Reverse: CTTTCATACTGCTGGCGCAC
