# Supplementary material for: Cardiomyocyte cytosolic nuclear self-DNA contributes to the pathogenesis of desmoplakin cardiomyopathy
Source: JCI Insight. 2025 Jul 3;10(16):e192283. doi: 10.1172/jci.insight.192283 (PMC12406735; doi:10.1172/jci.insight.192283)

Figure 4

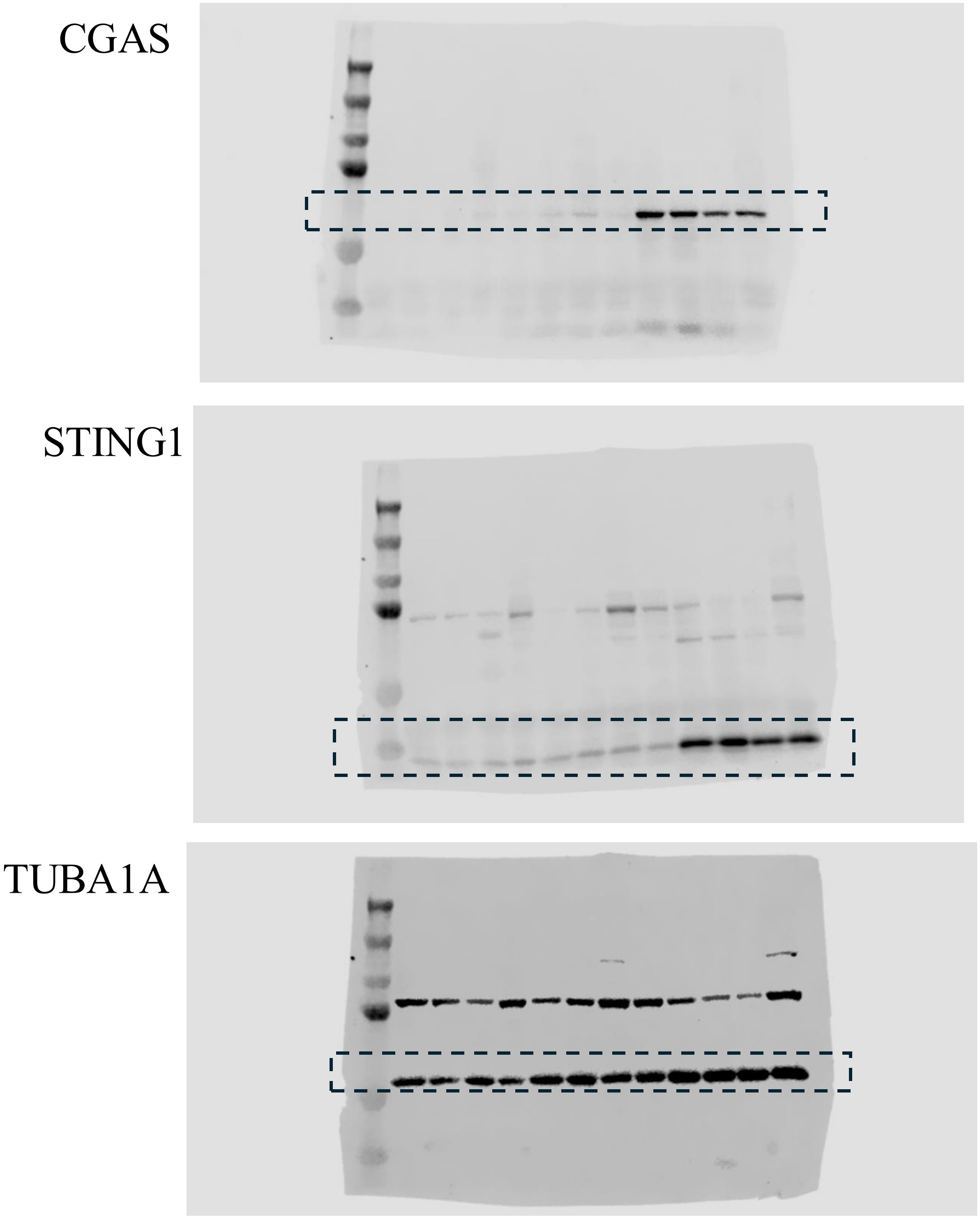

Figure 4

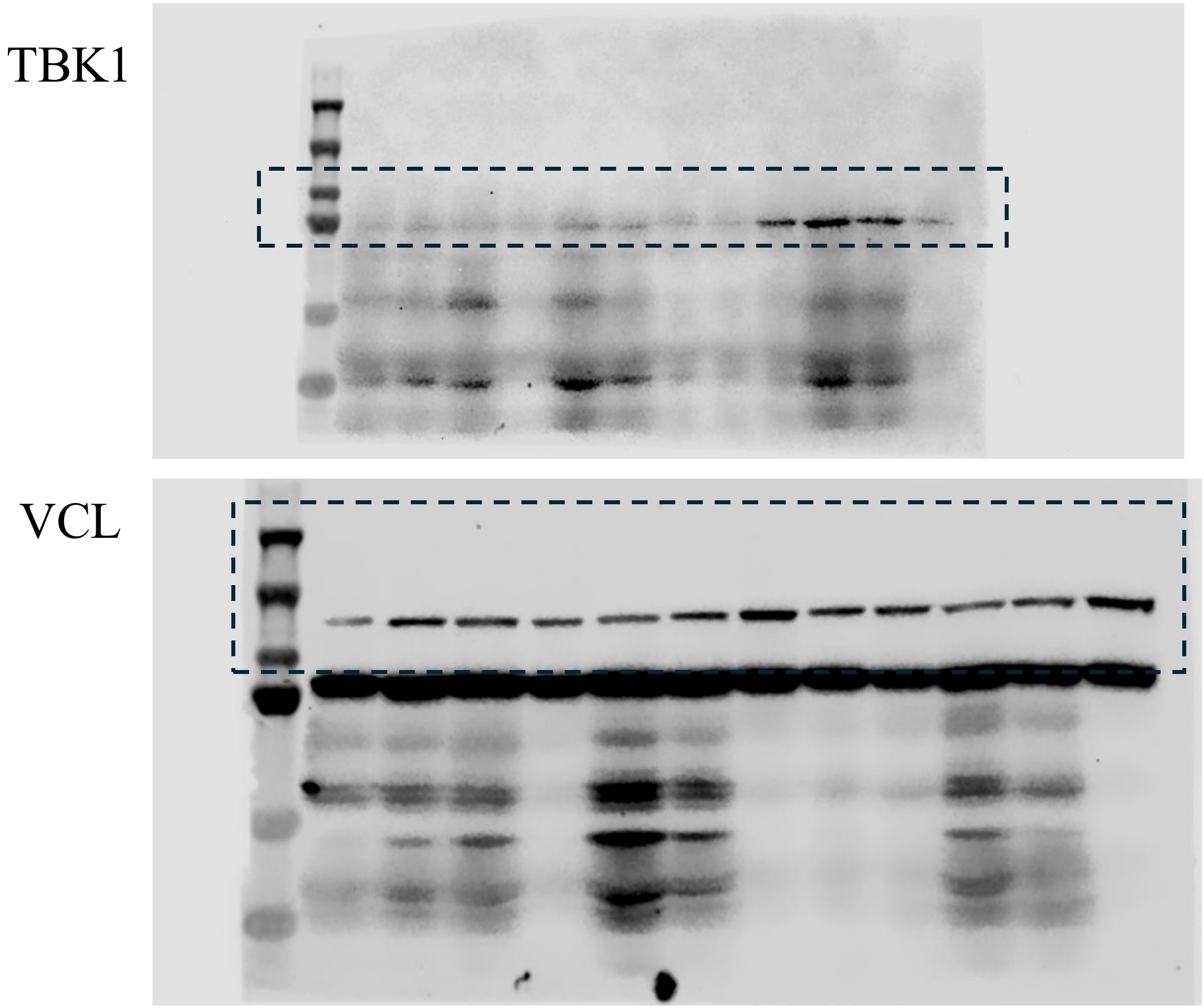

Figure 4

IRF3

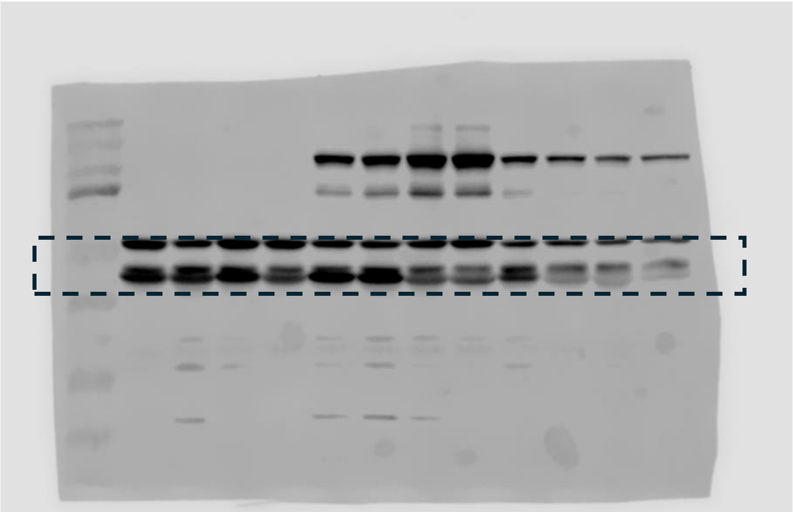

pIRF3-S396

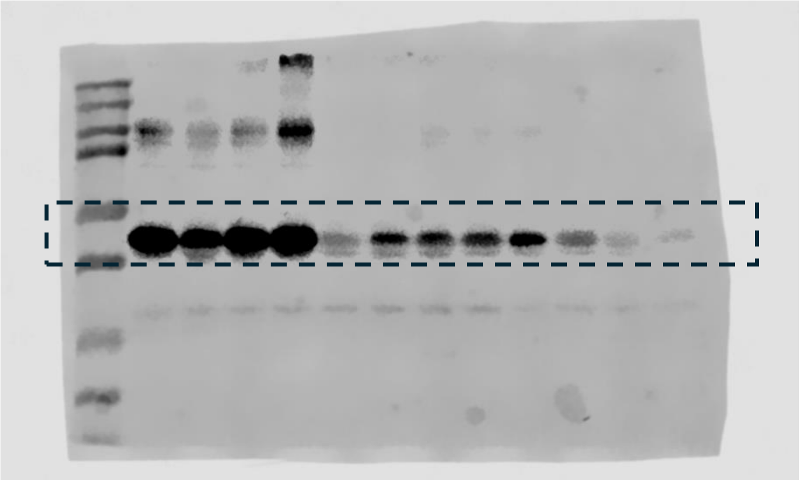

VCL

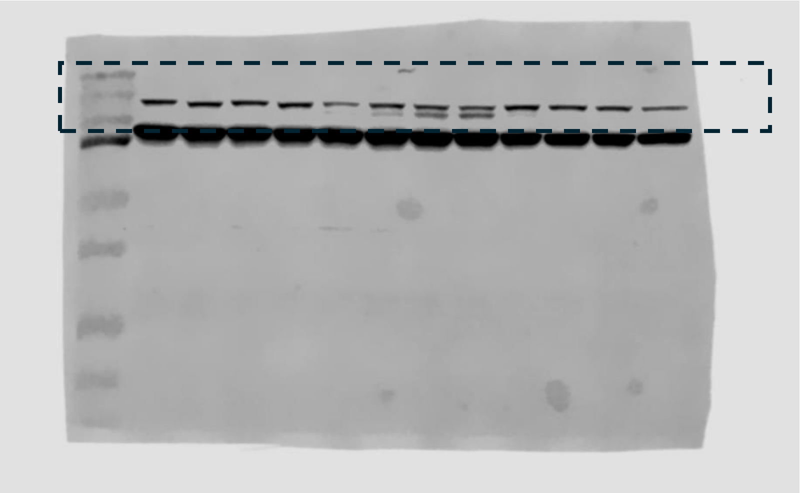

Figure 4

pIRF3-386

VCL

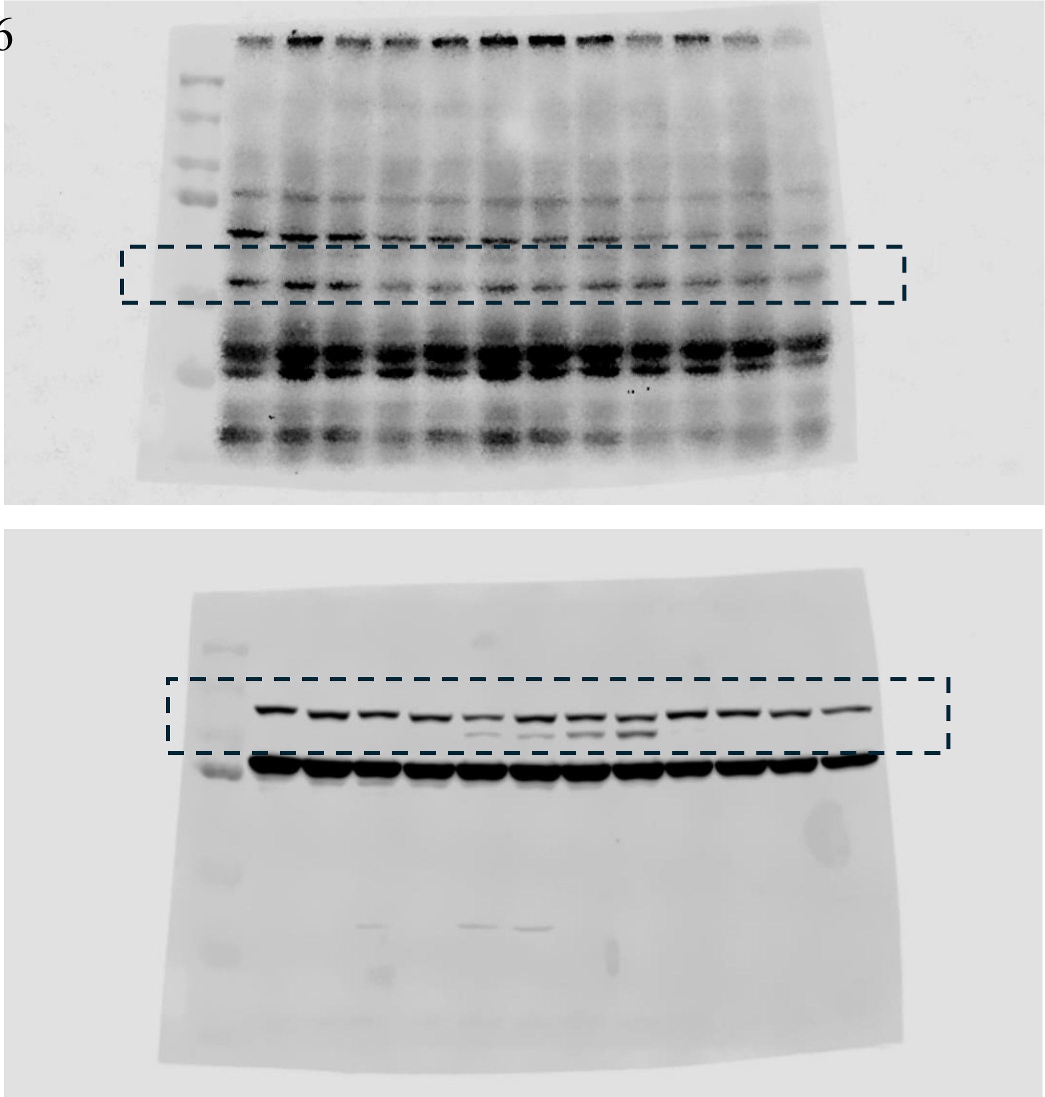

Figure 5

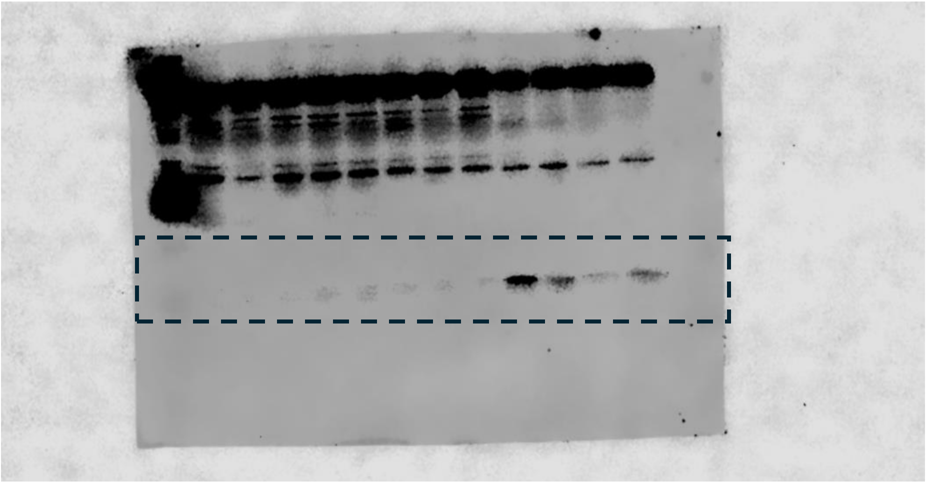

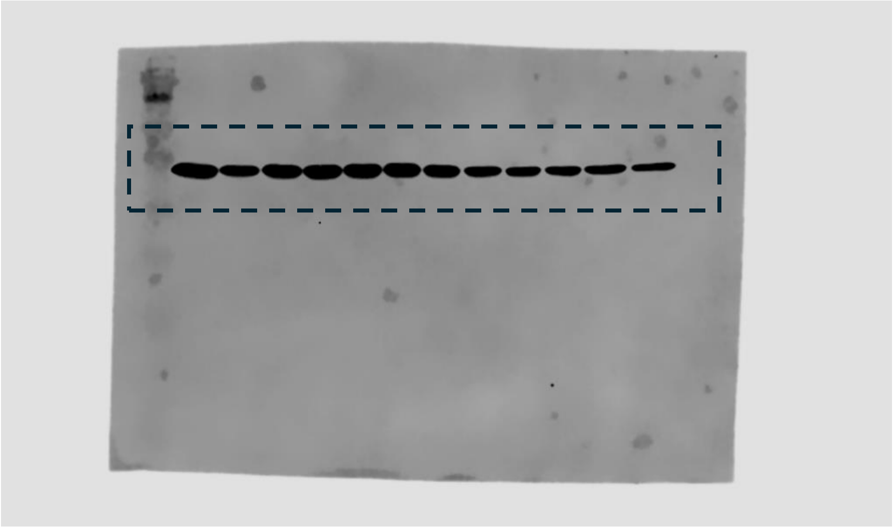

Figure 5

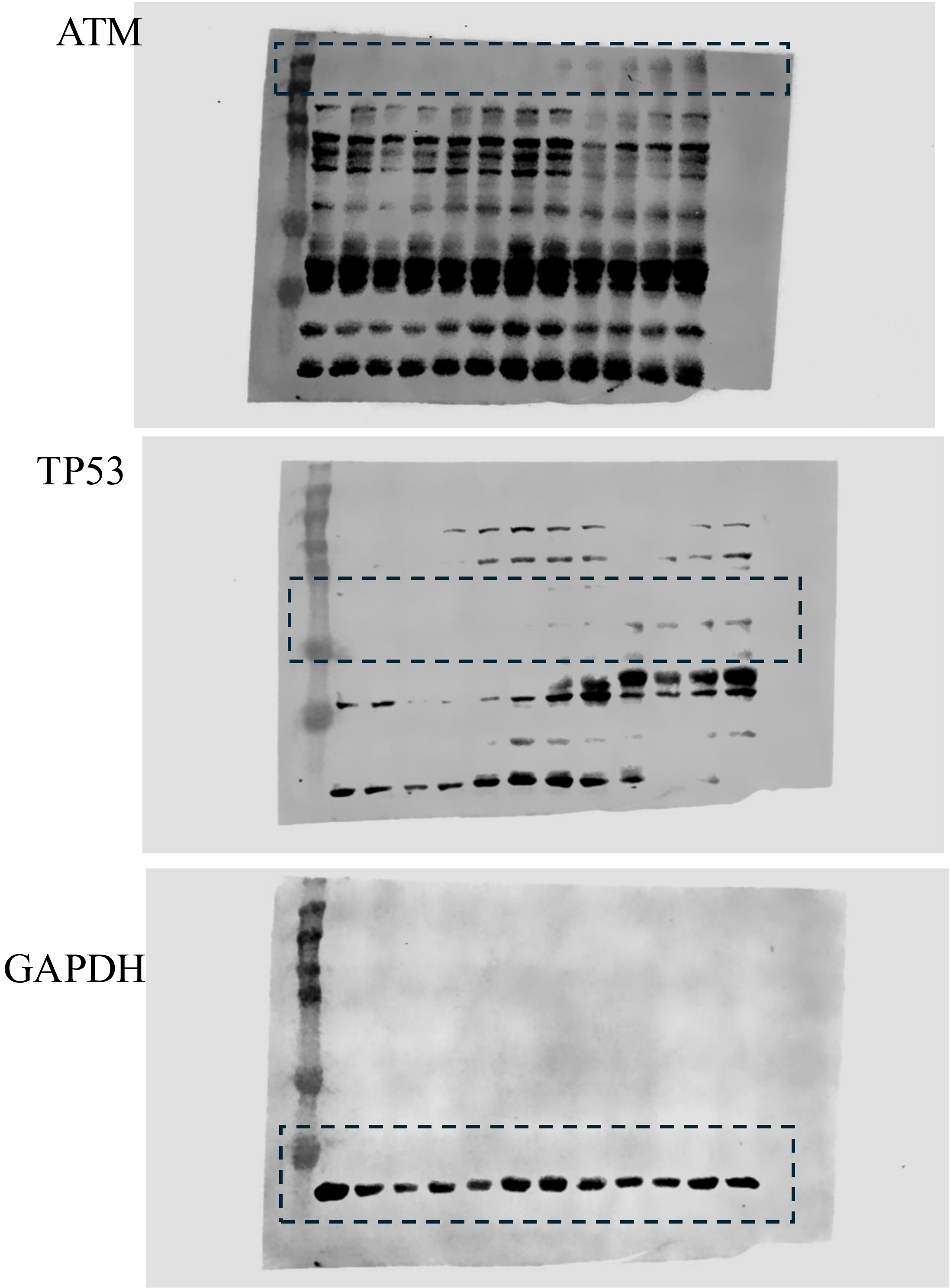

Figure 5

CDKN1A

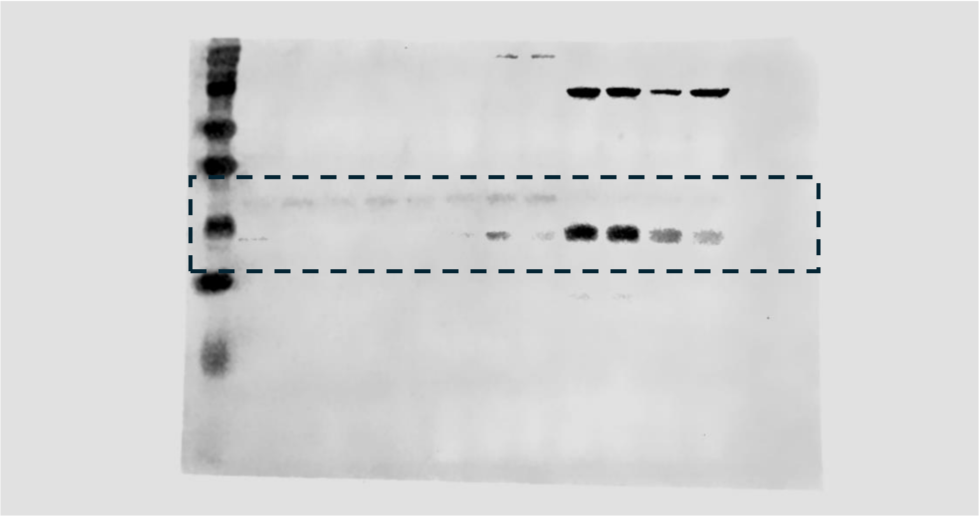

GAPDH

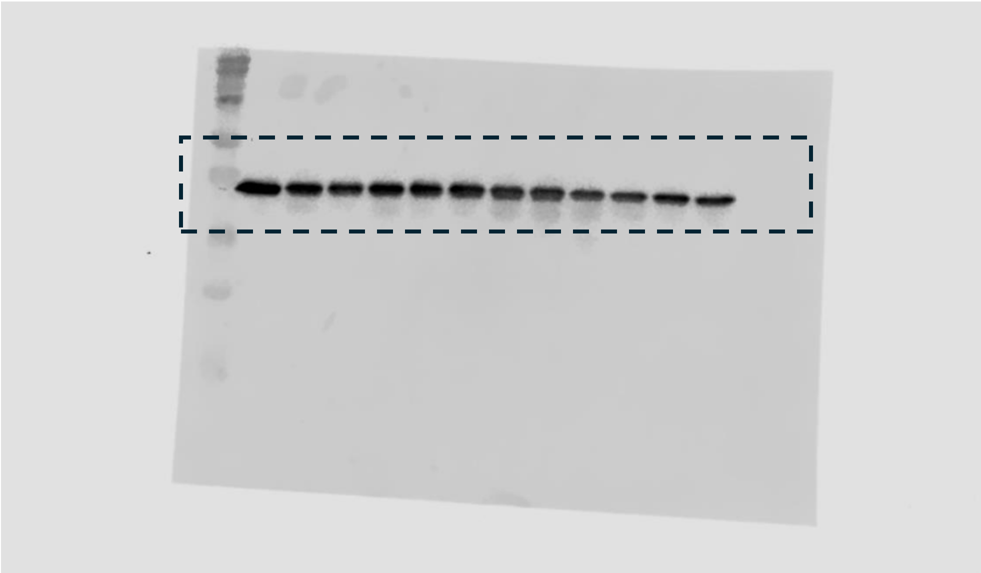

Figure 6

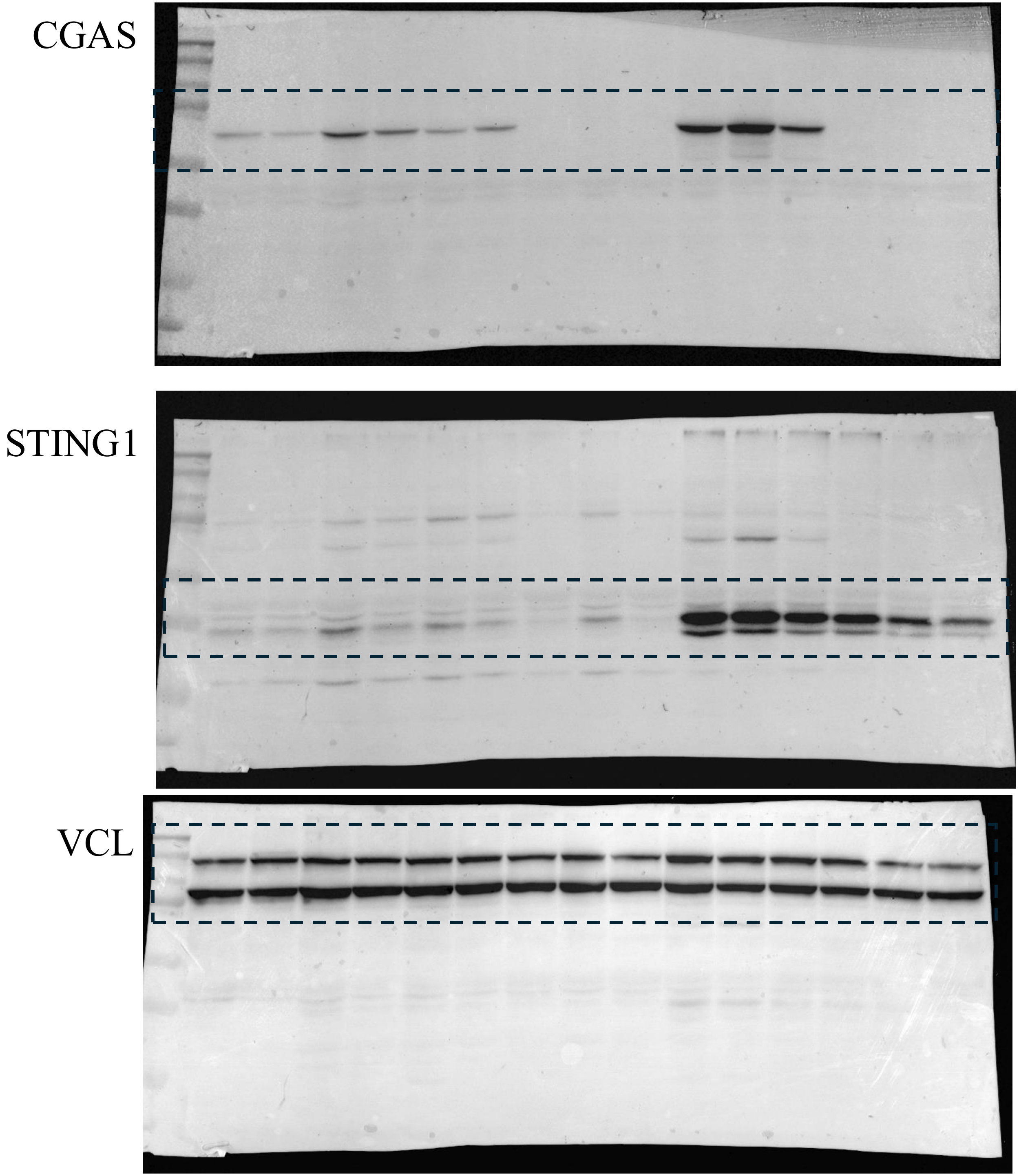

Figure 6

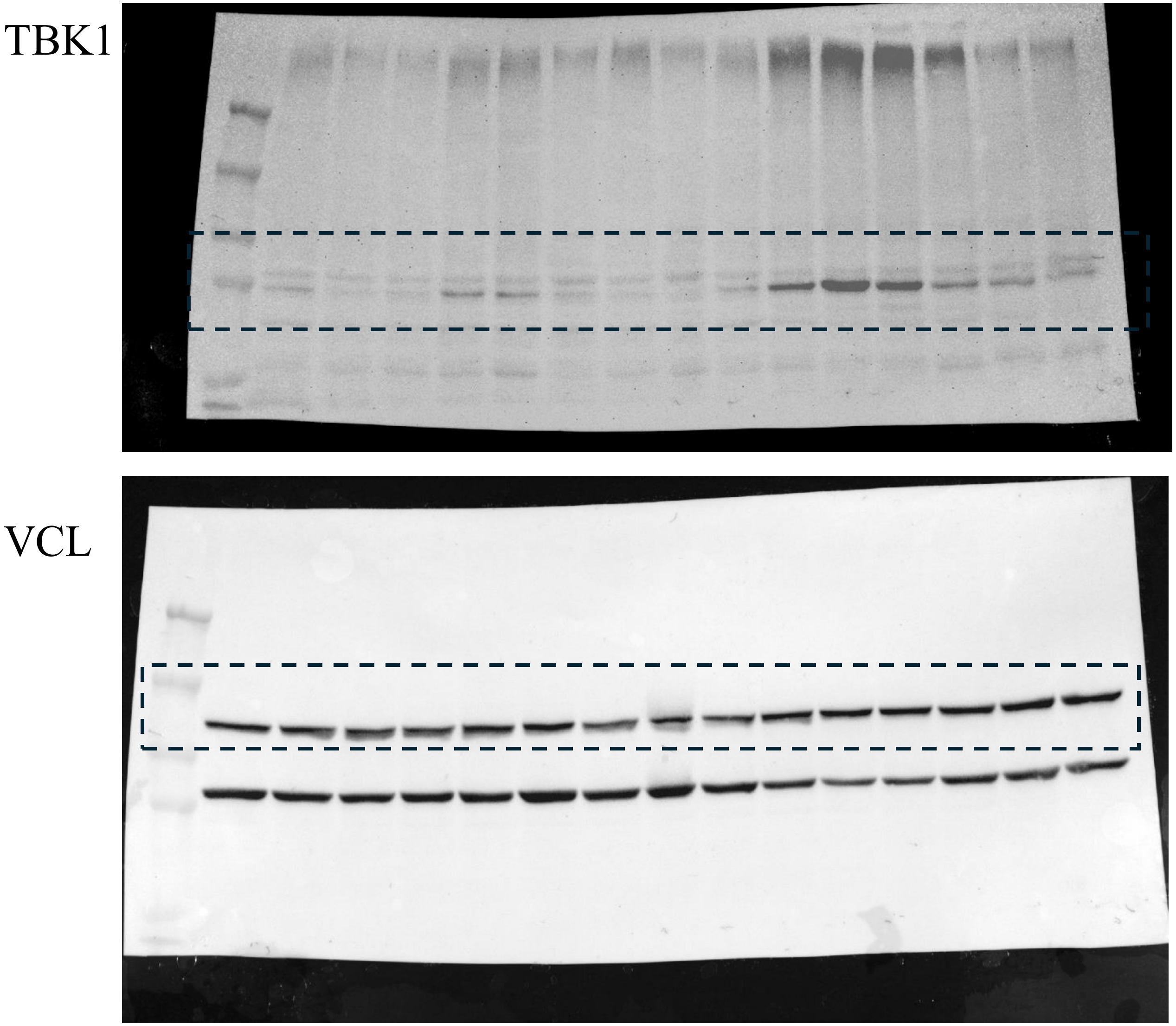

Figure 6

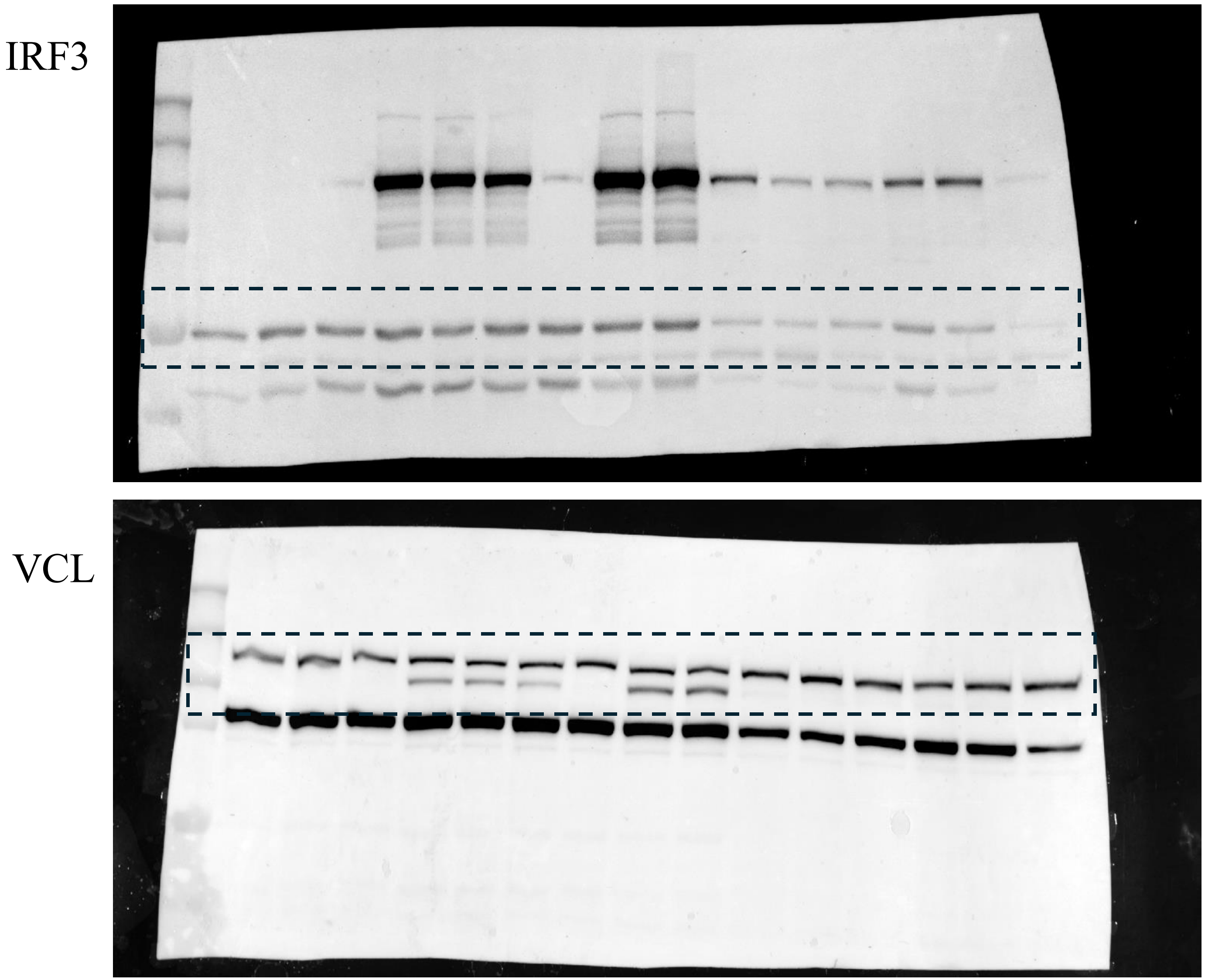

Figure 6

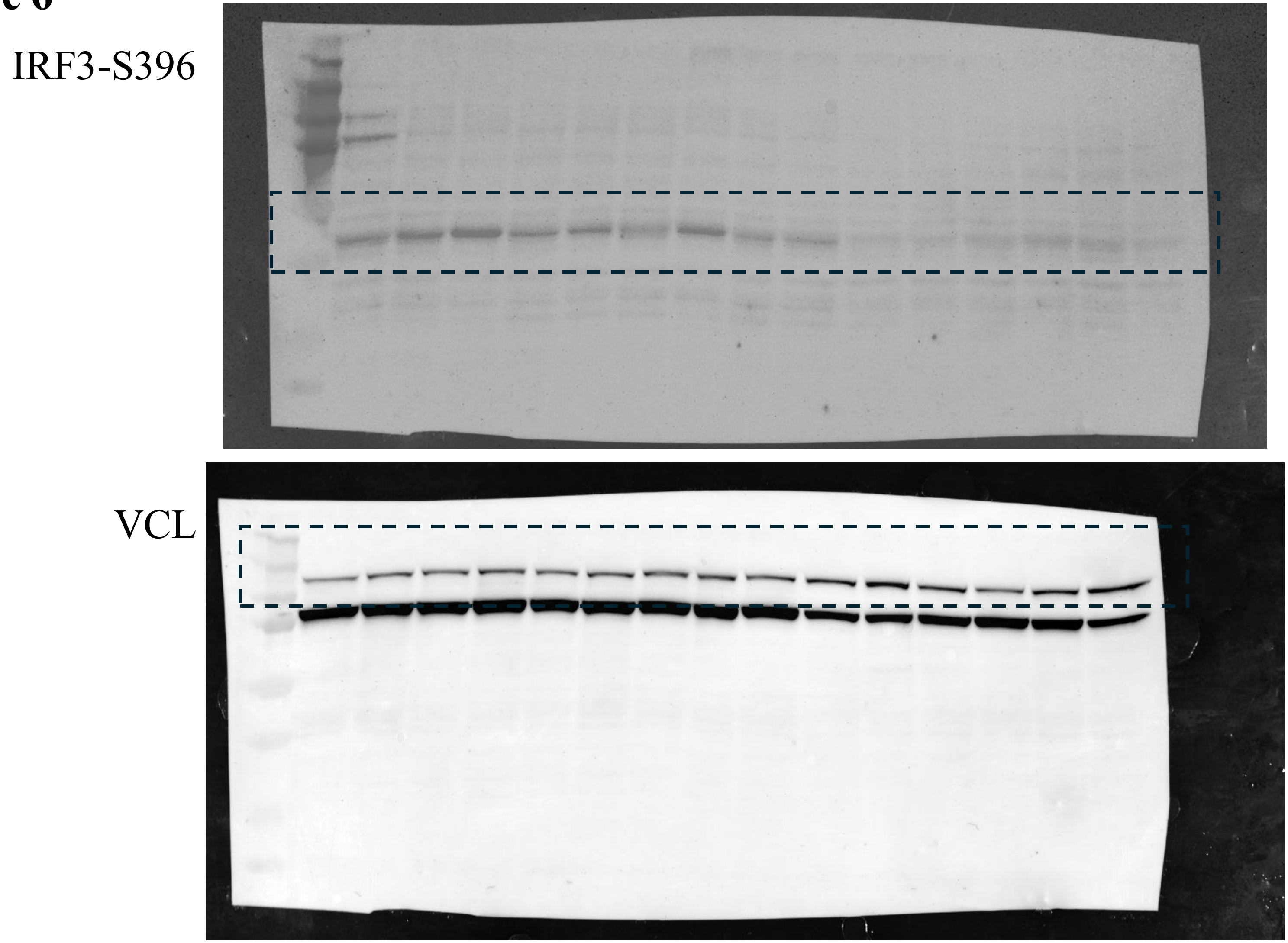

Figure 6

pIRF3-S386

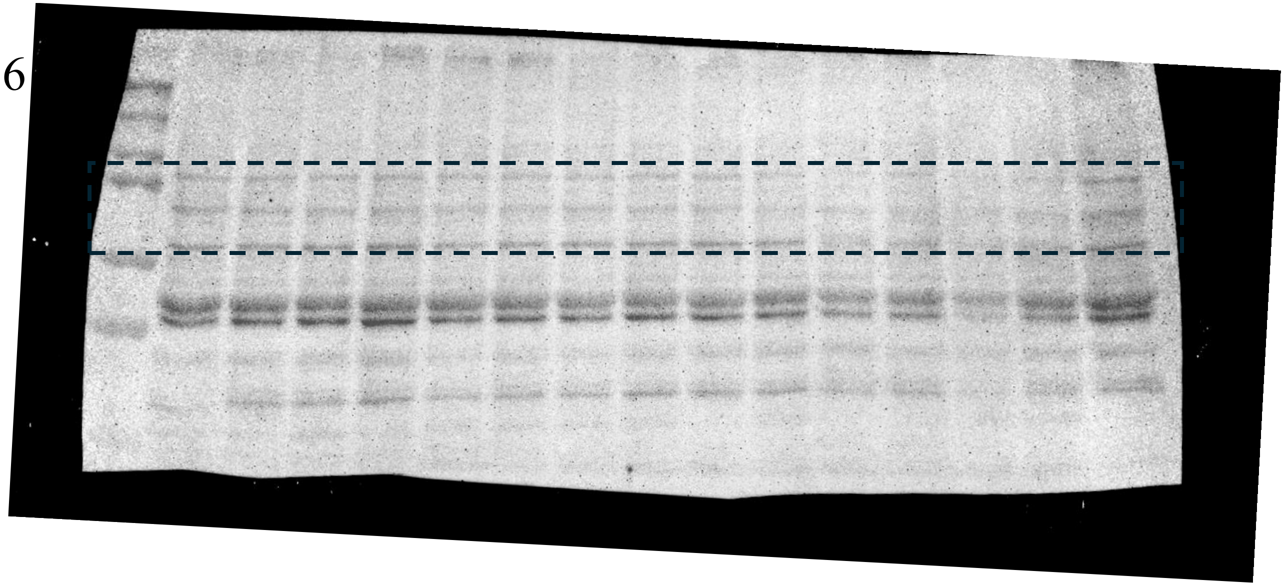

VCL

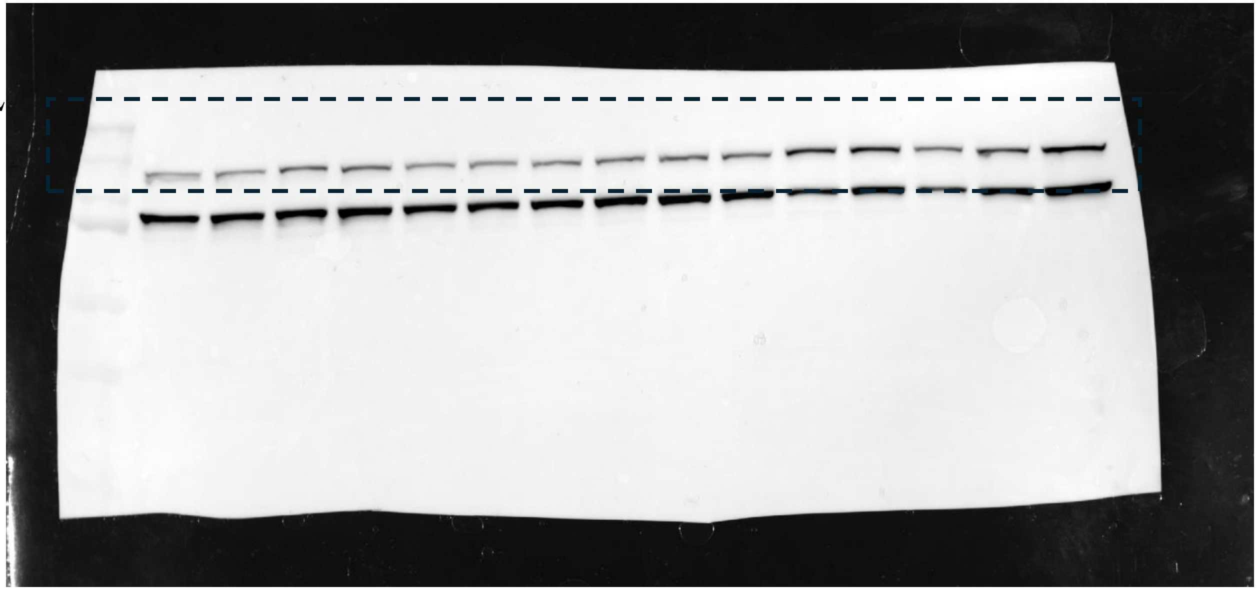

Figure 7

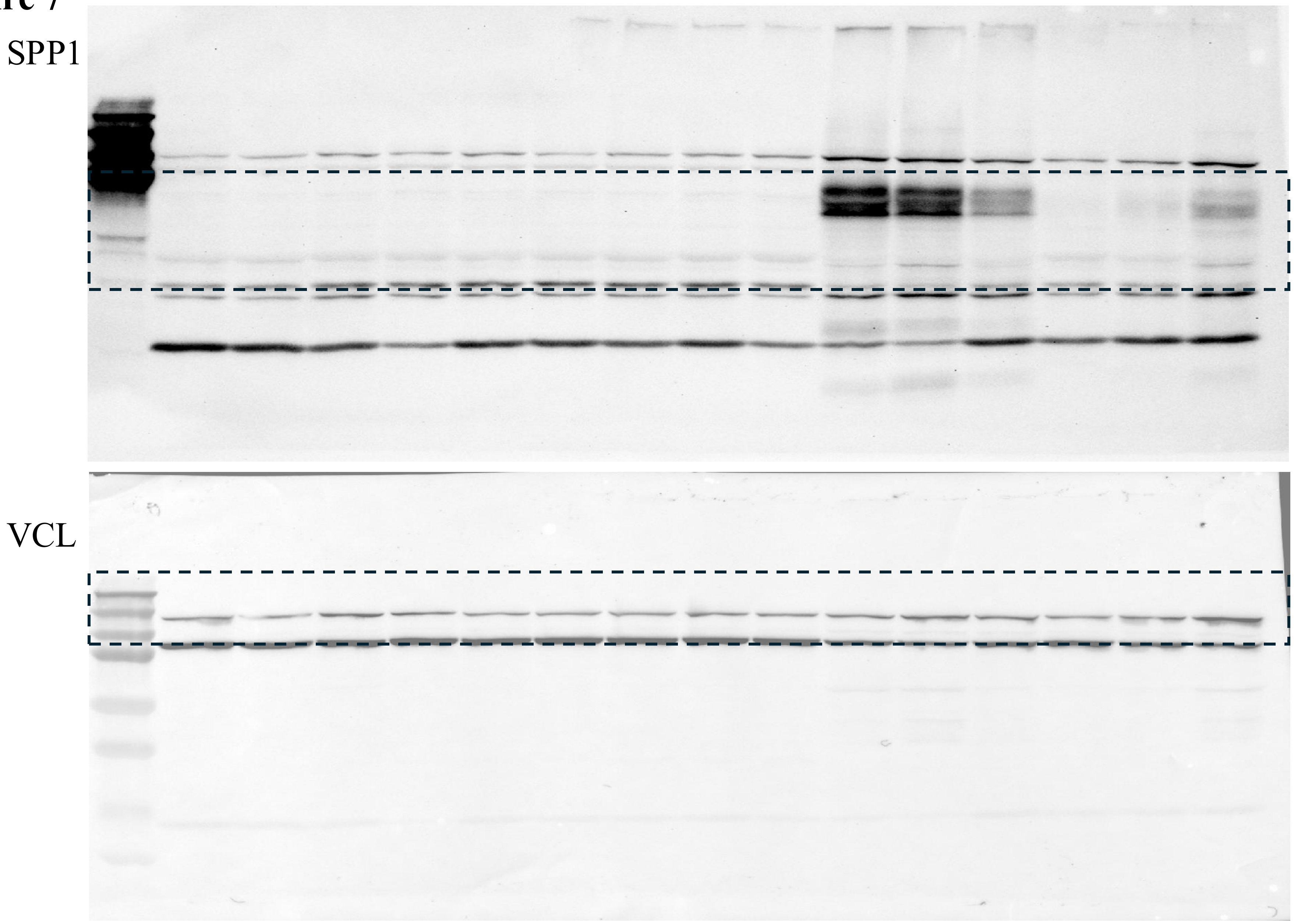

Figure 7

SFRP3

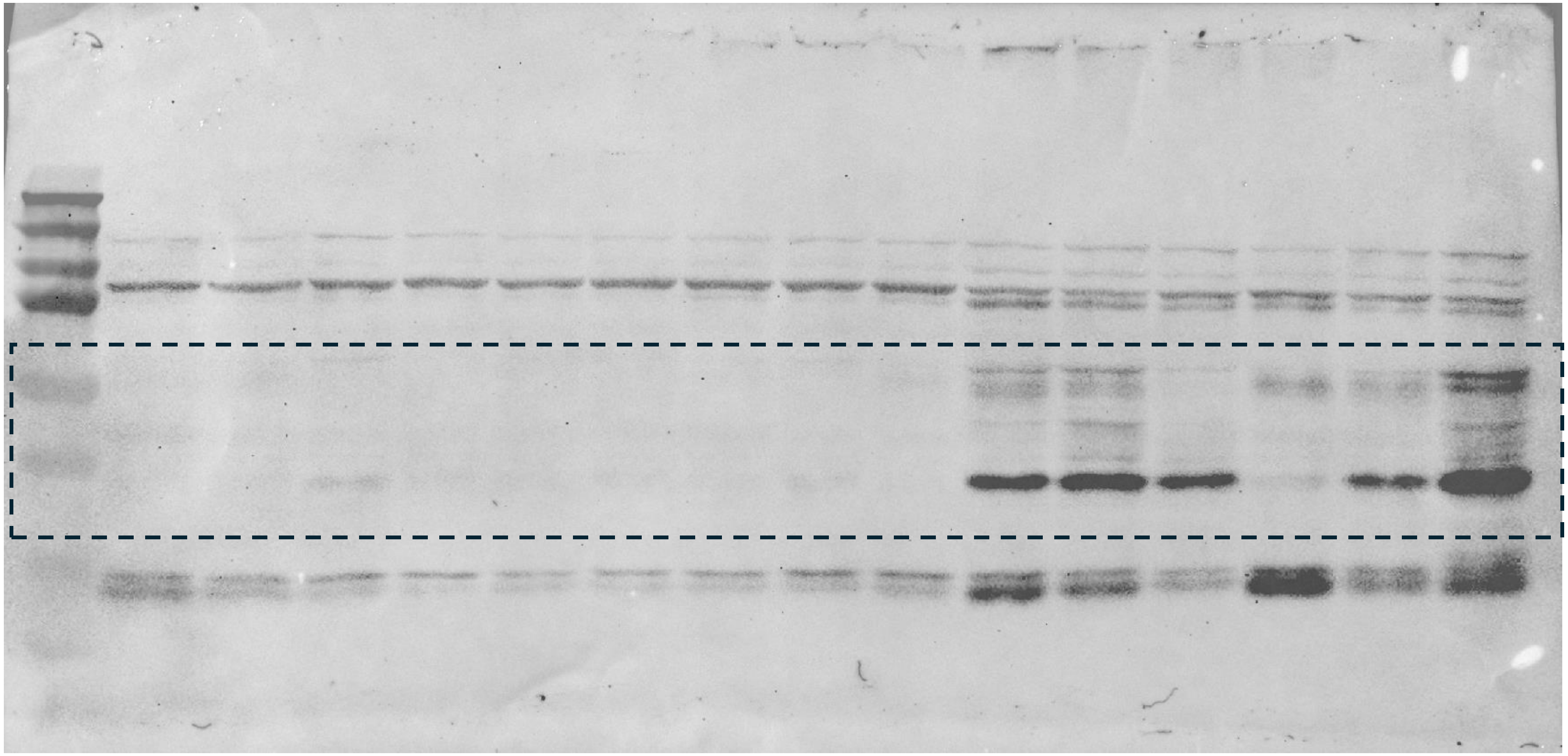

VCL

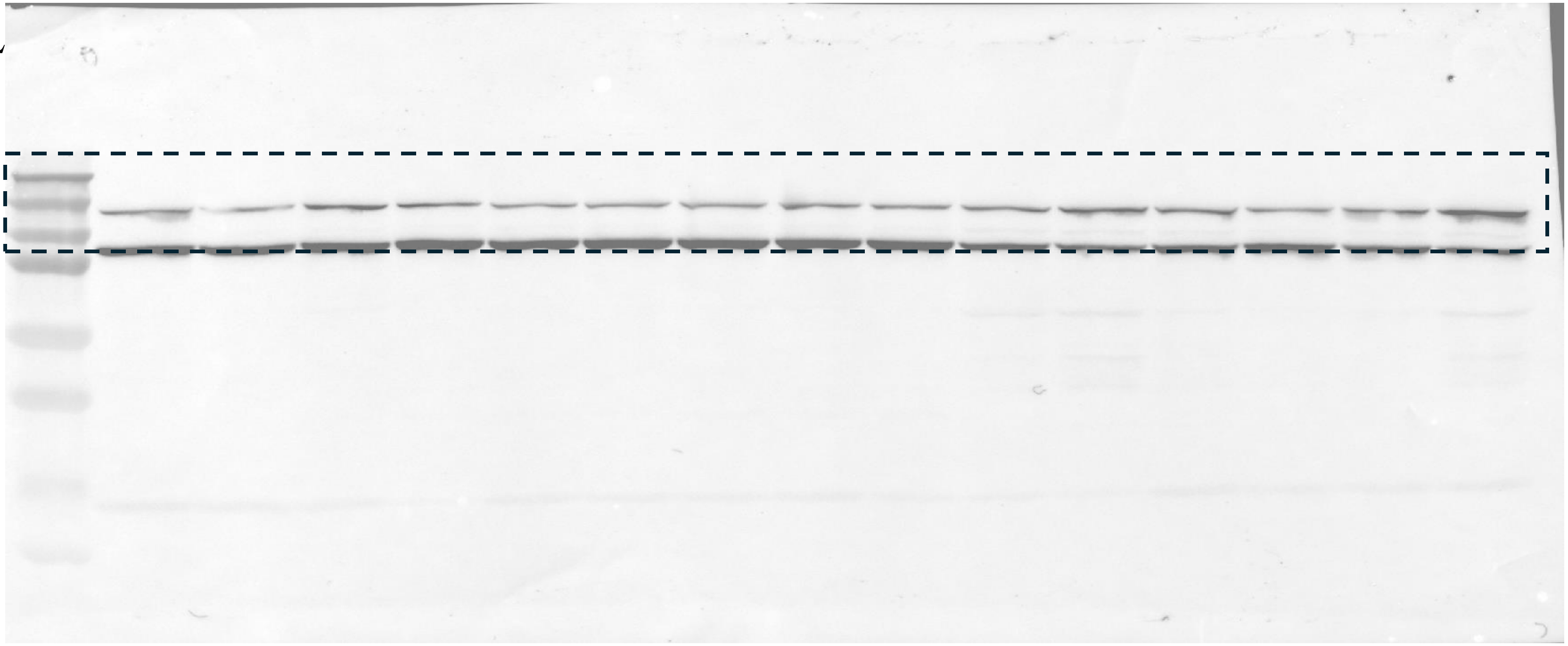

Figure 7

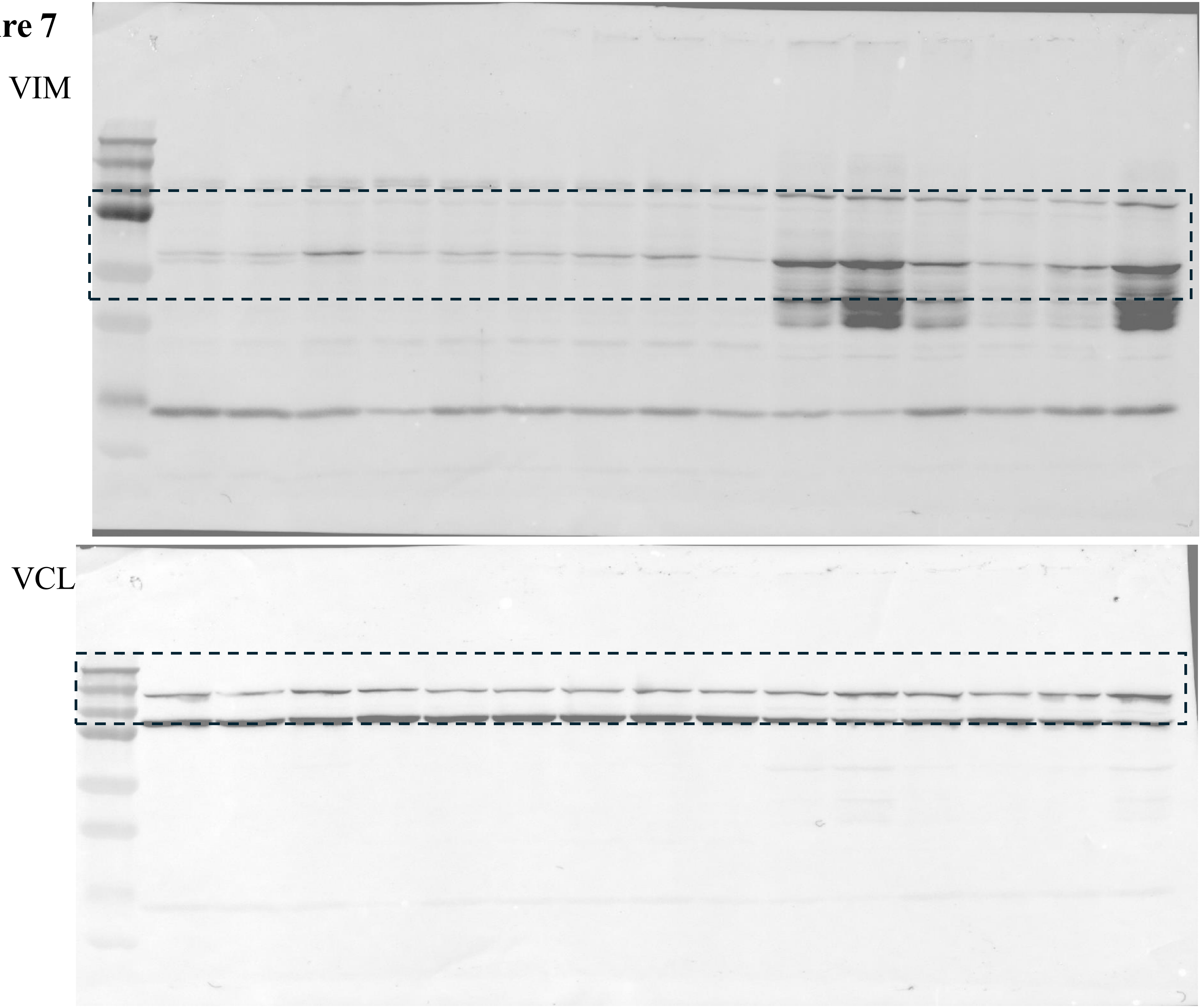

Figure 7

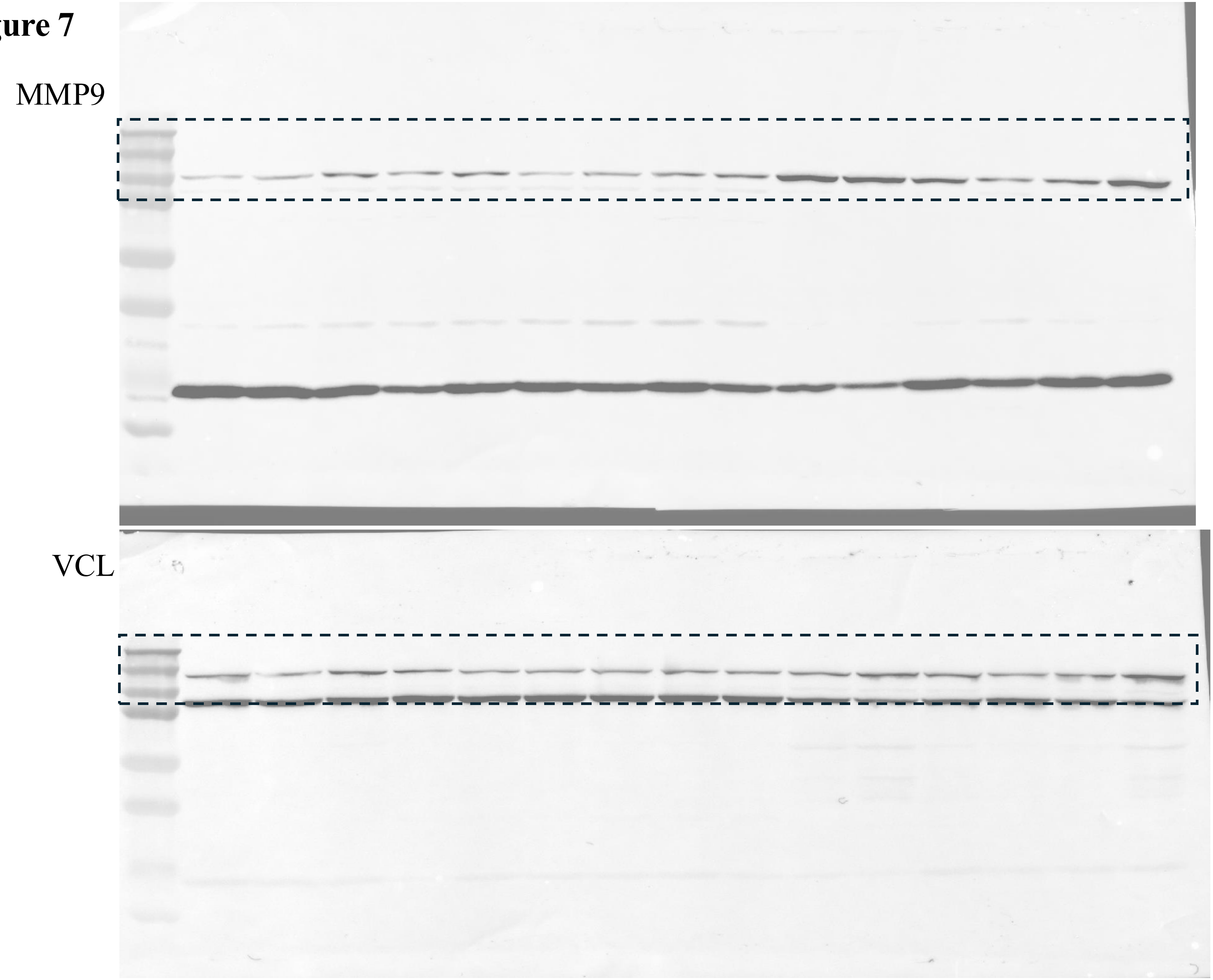

Figure 8 MMP9

cardiomyocytes

SPP1

VCL

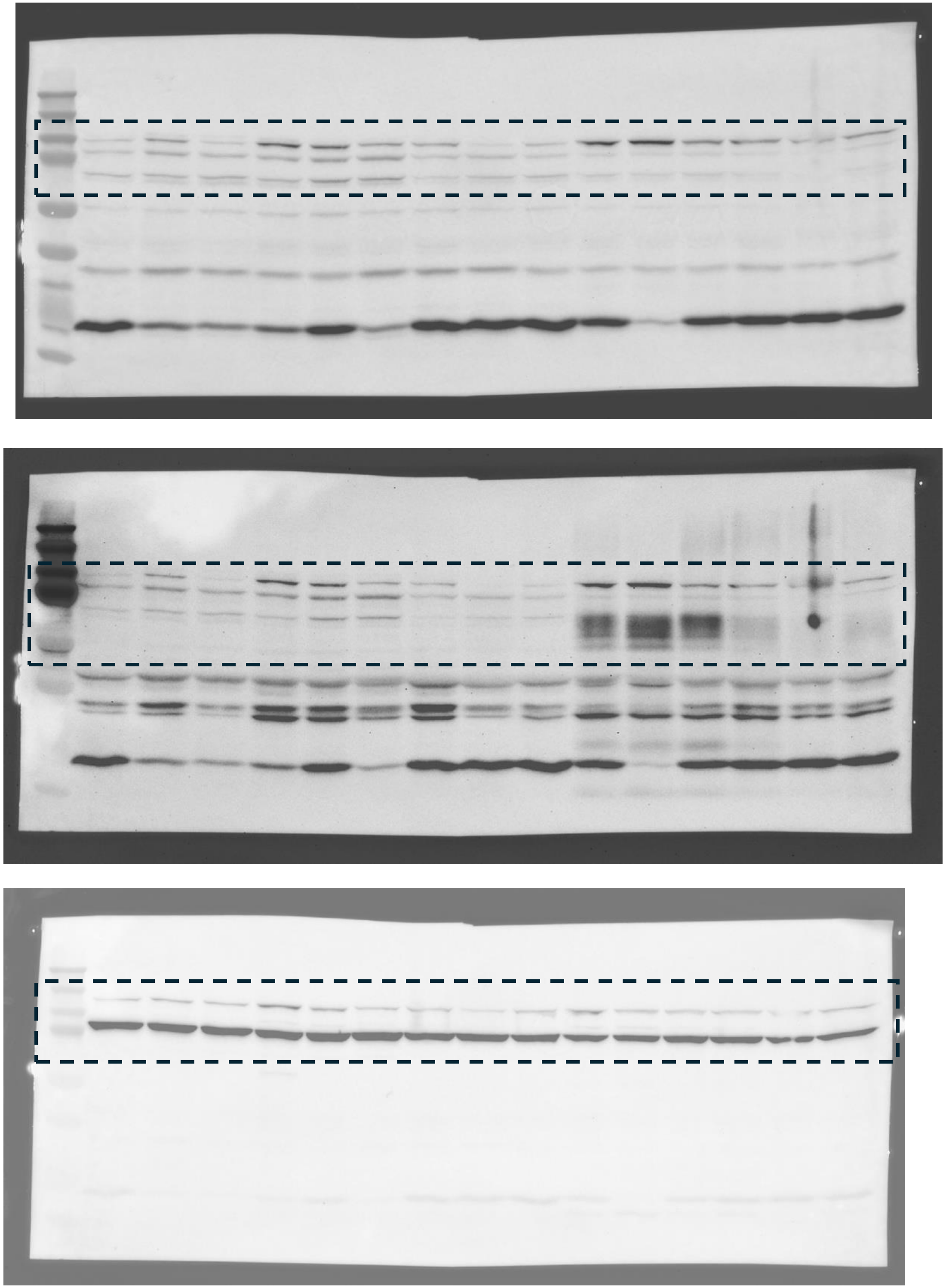

Figure 8

cardiomyocytes

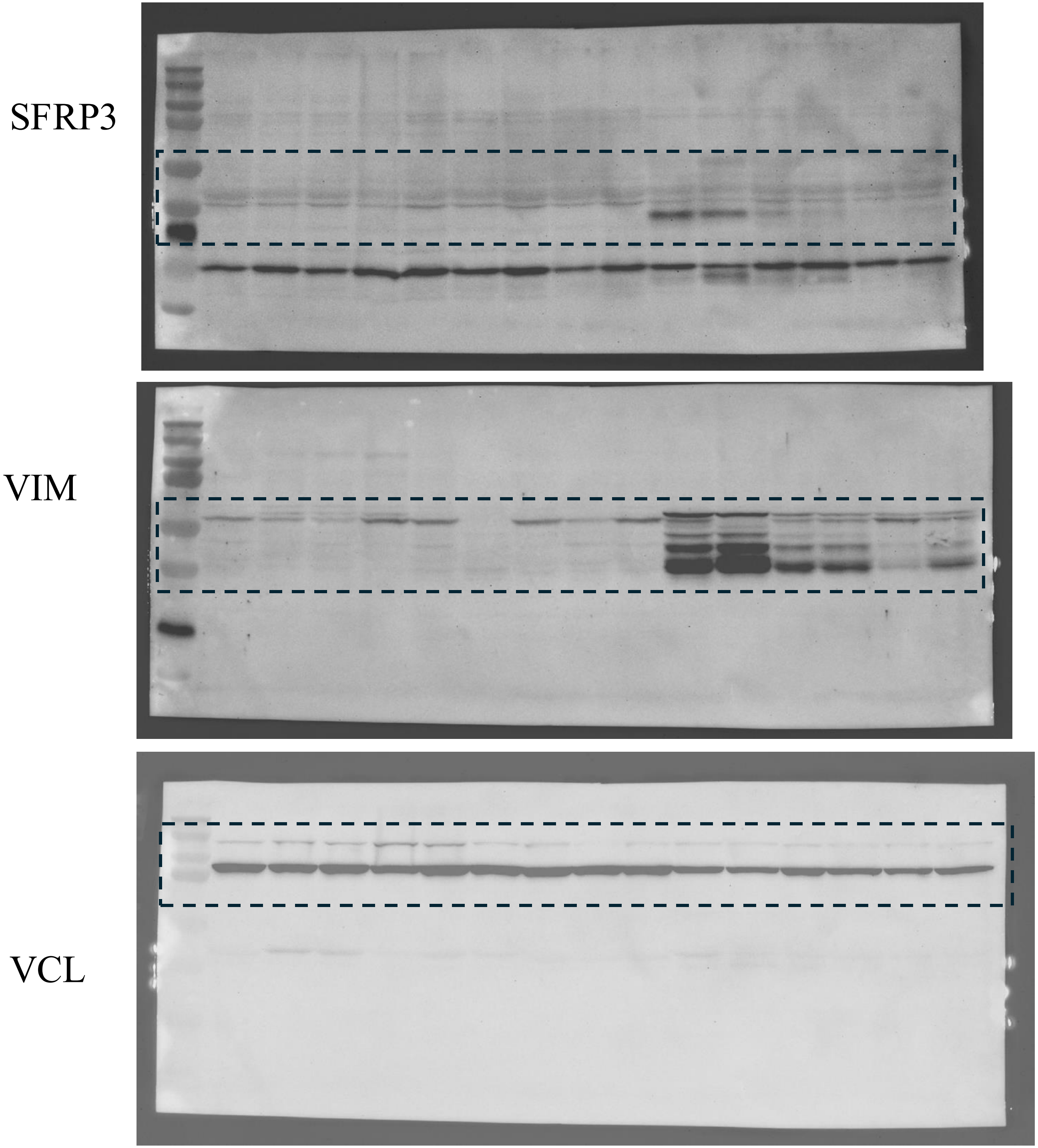

Figure 8 MMP9

Non-myocytes

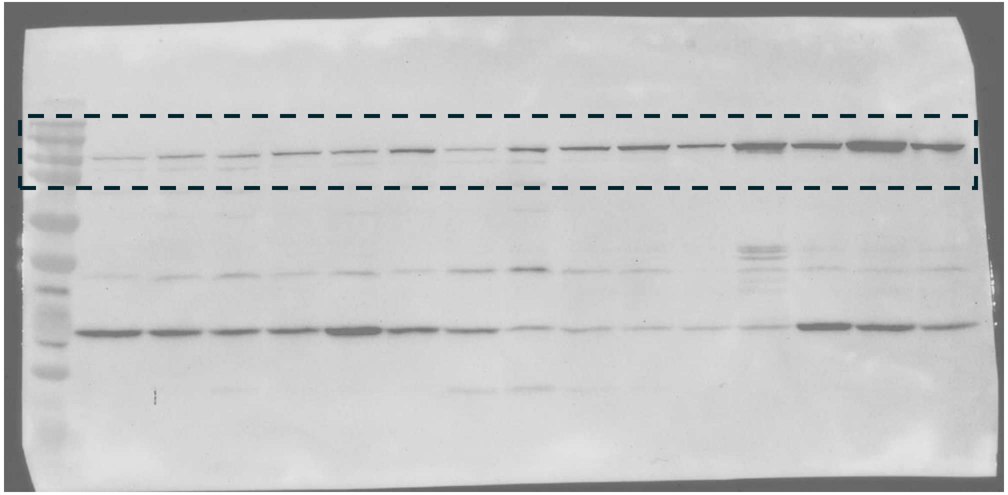

SPP1

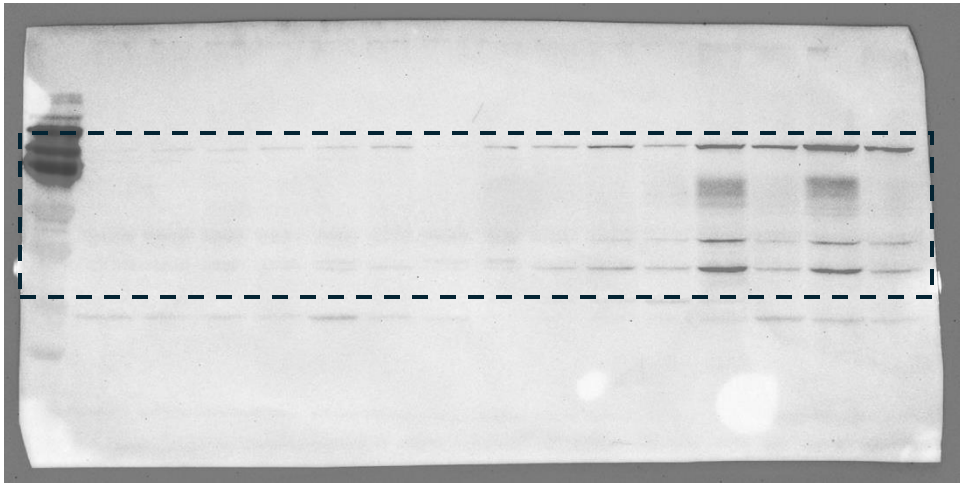

VCL

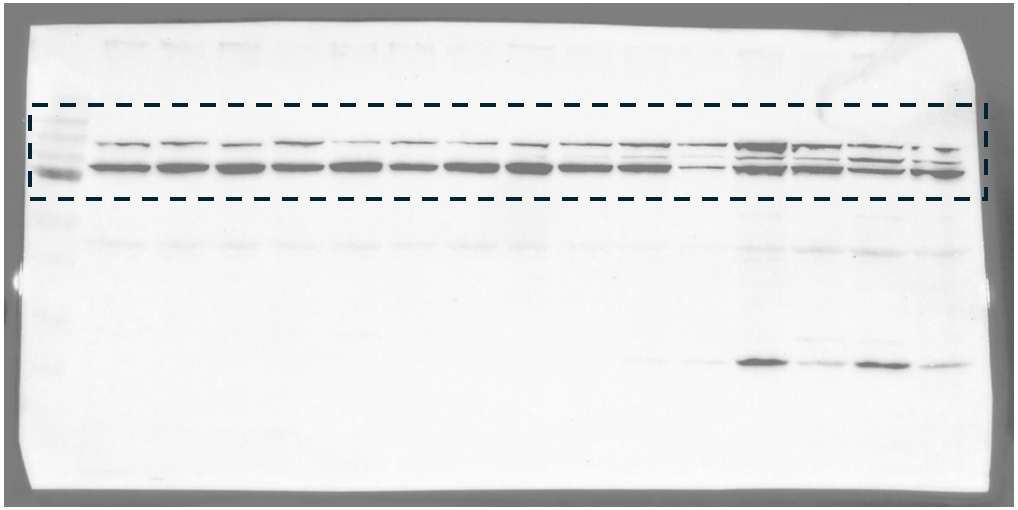

Figure 8

Non-myocytes

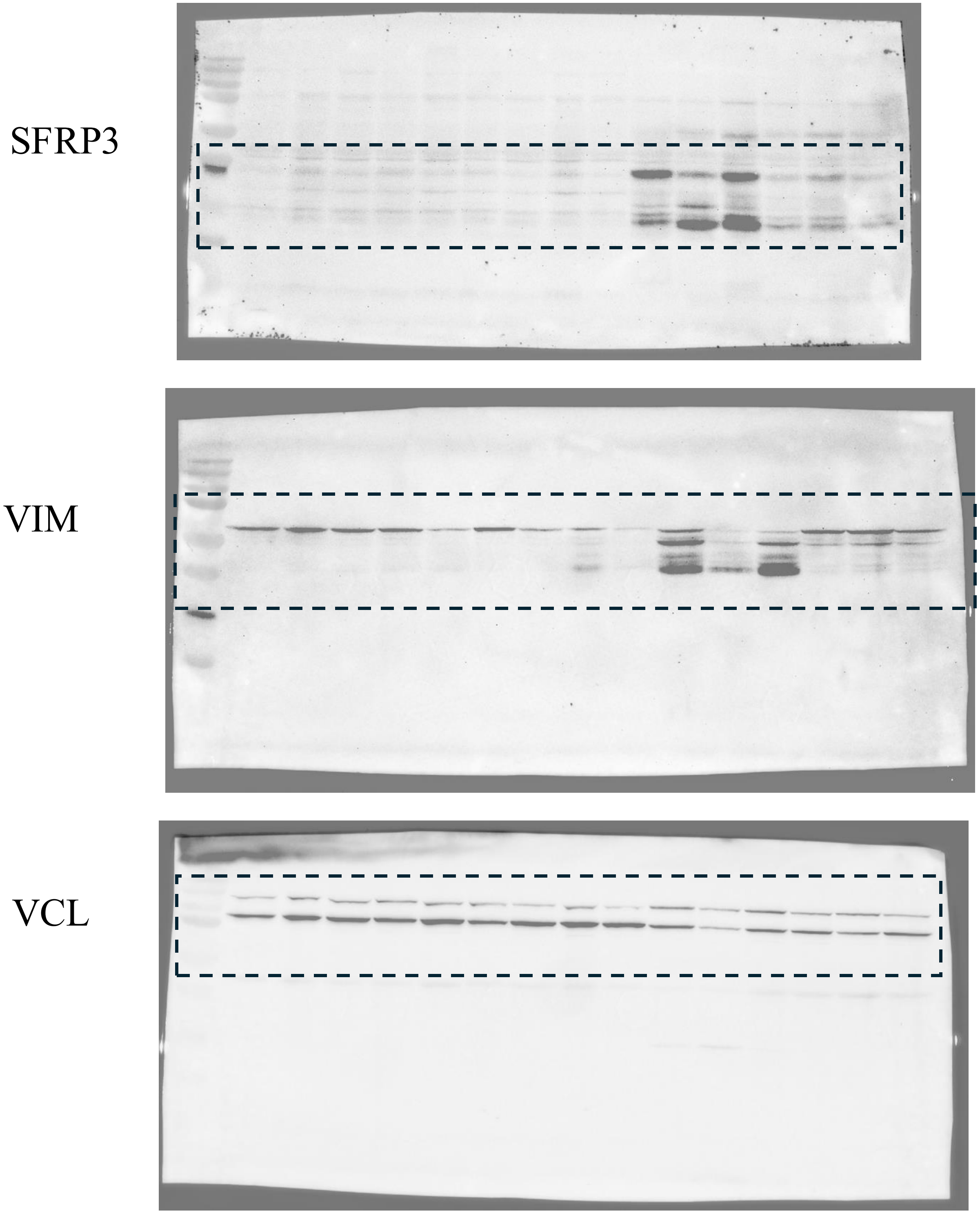

Figure 11

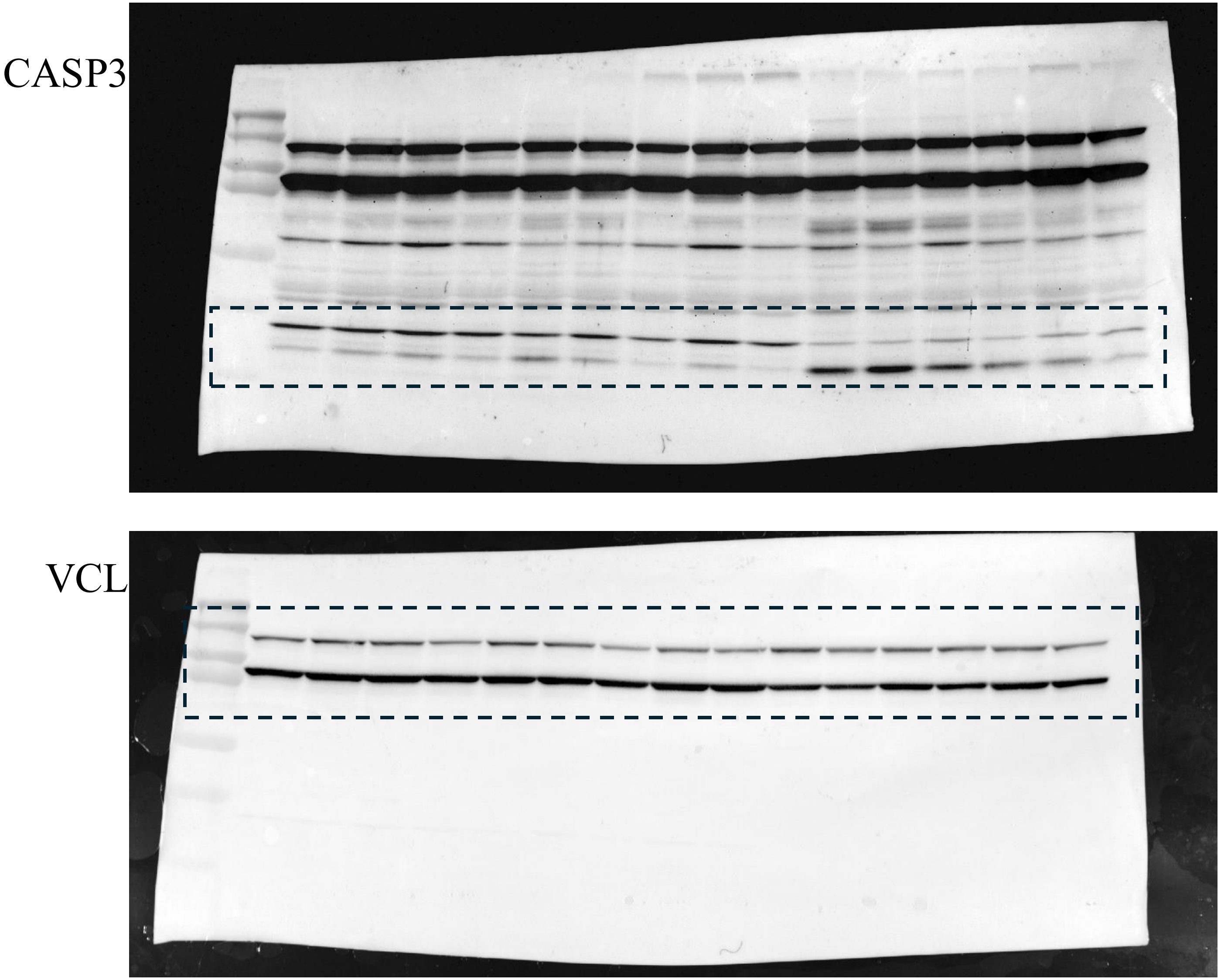

Figure 11

CASP8

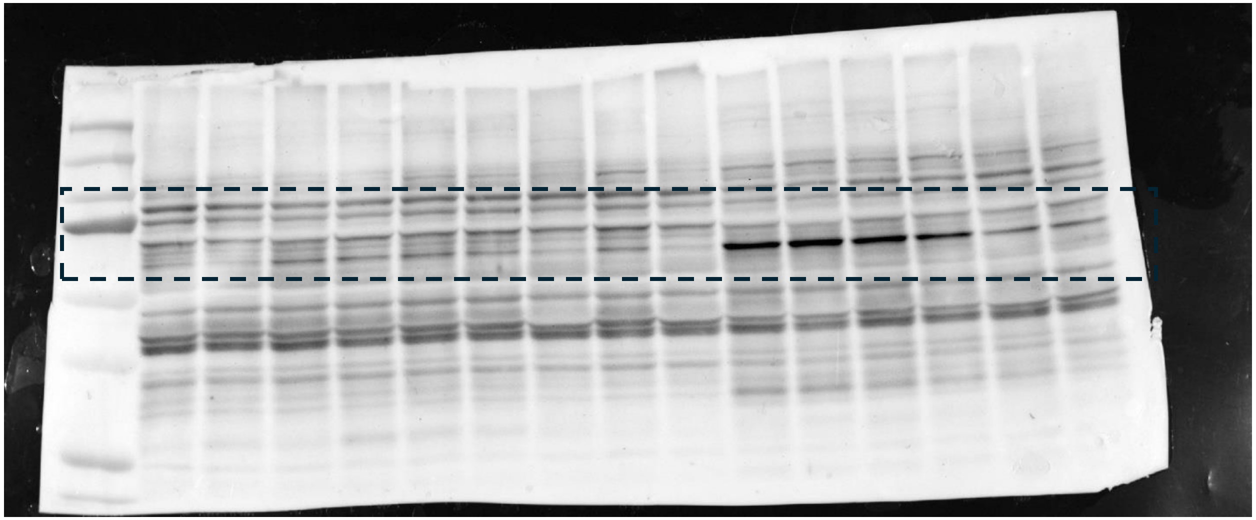

VCL

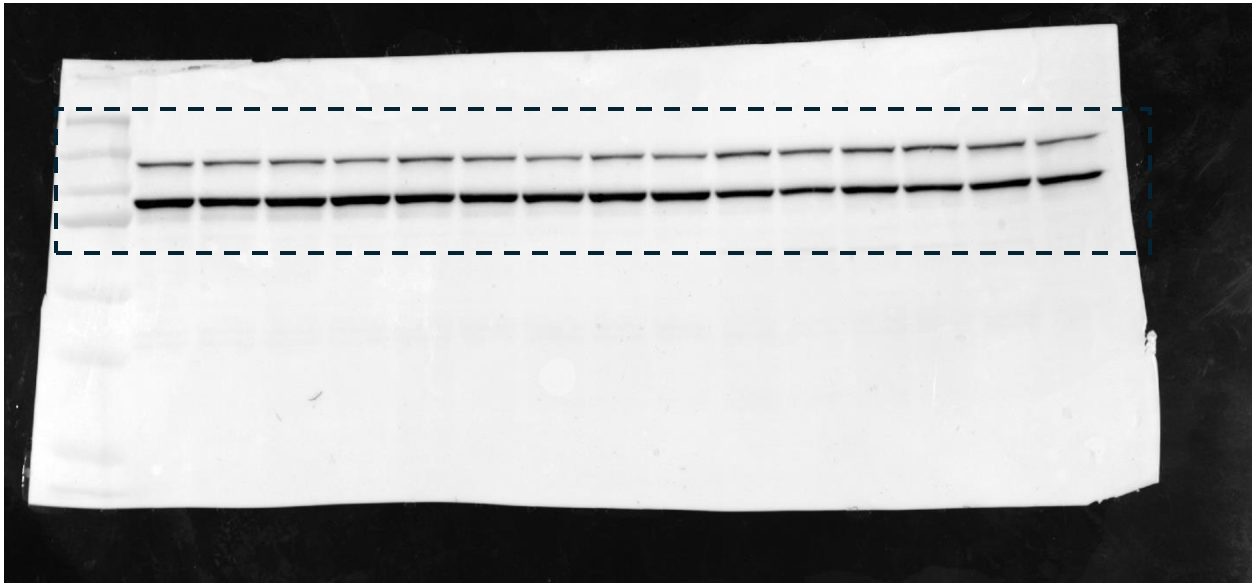

Figure 11

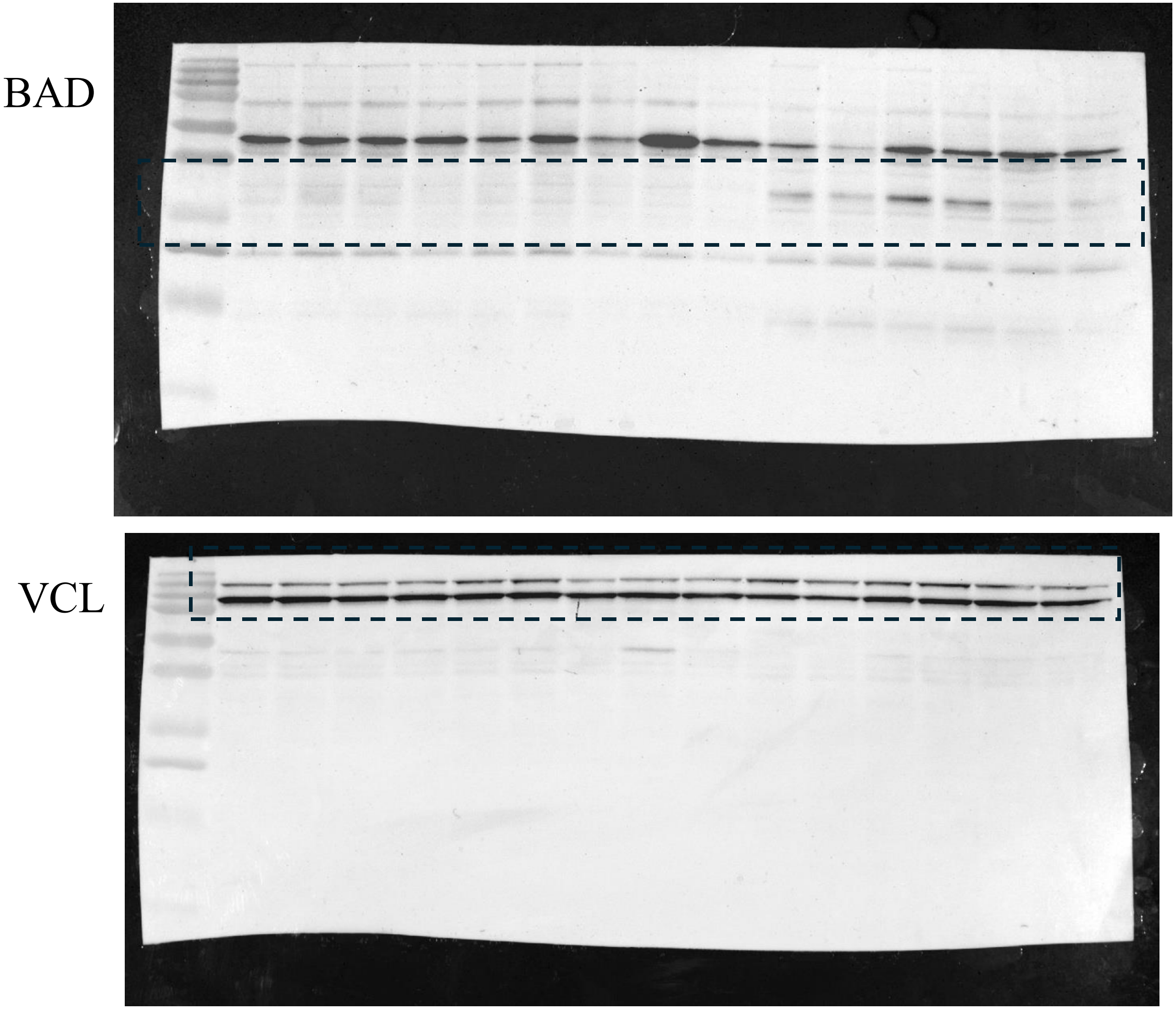

Figure 11

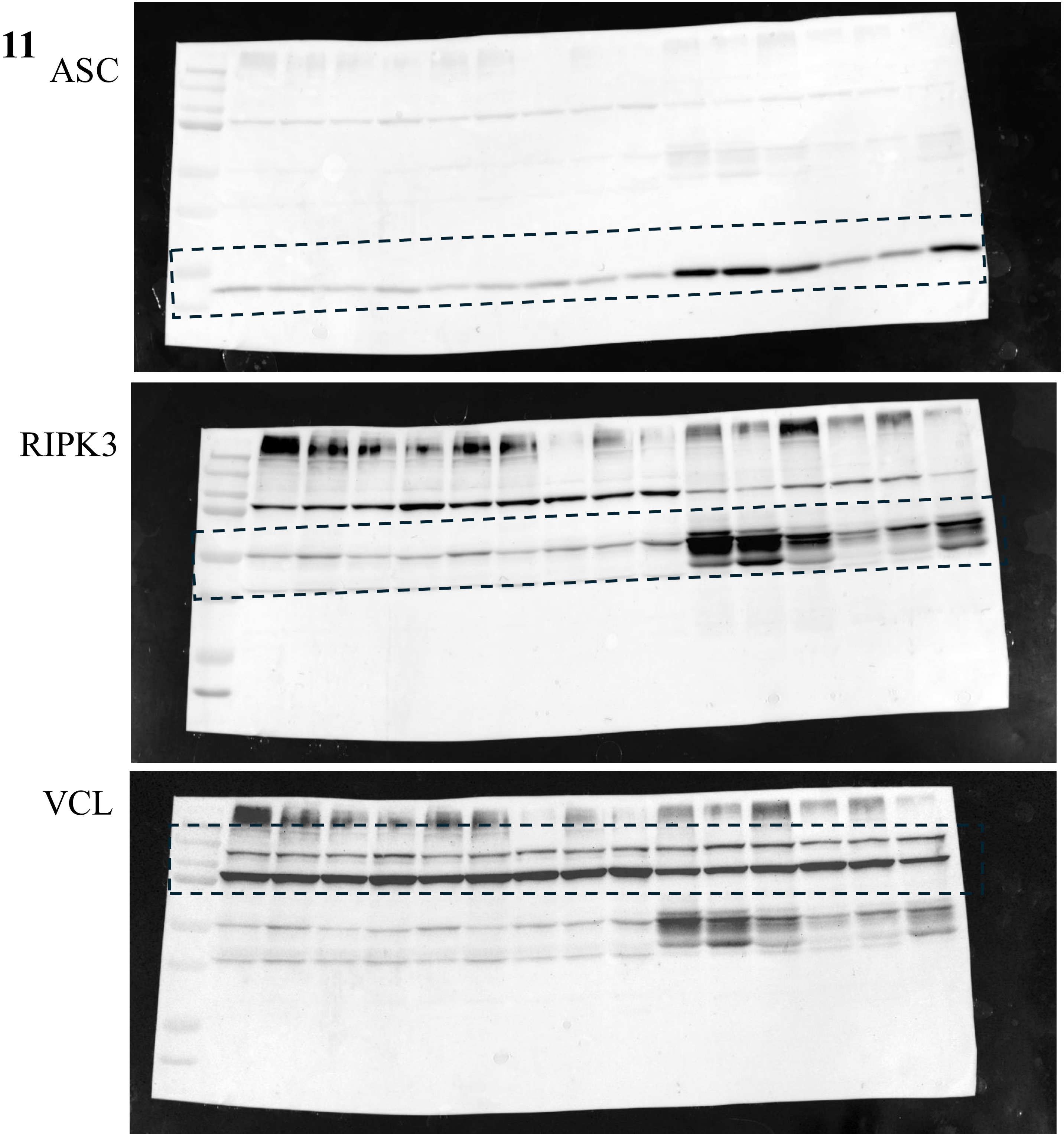

Figure 11

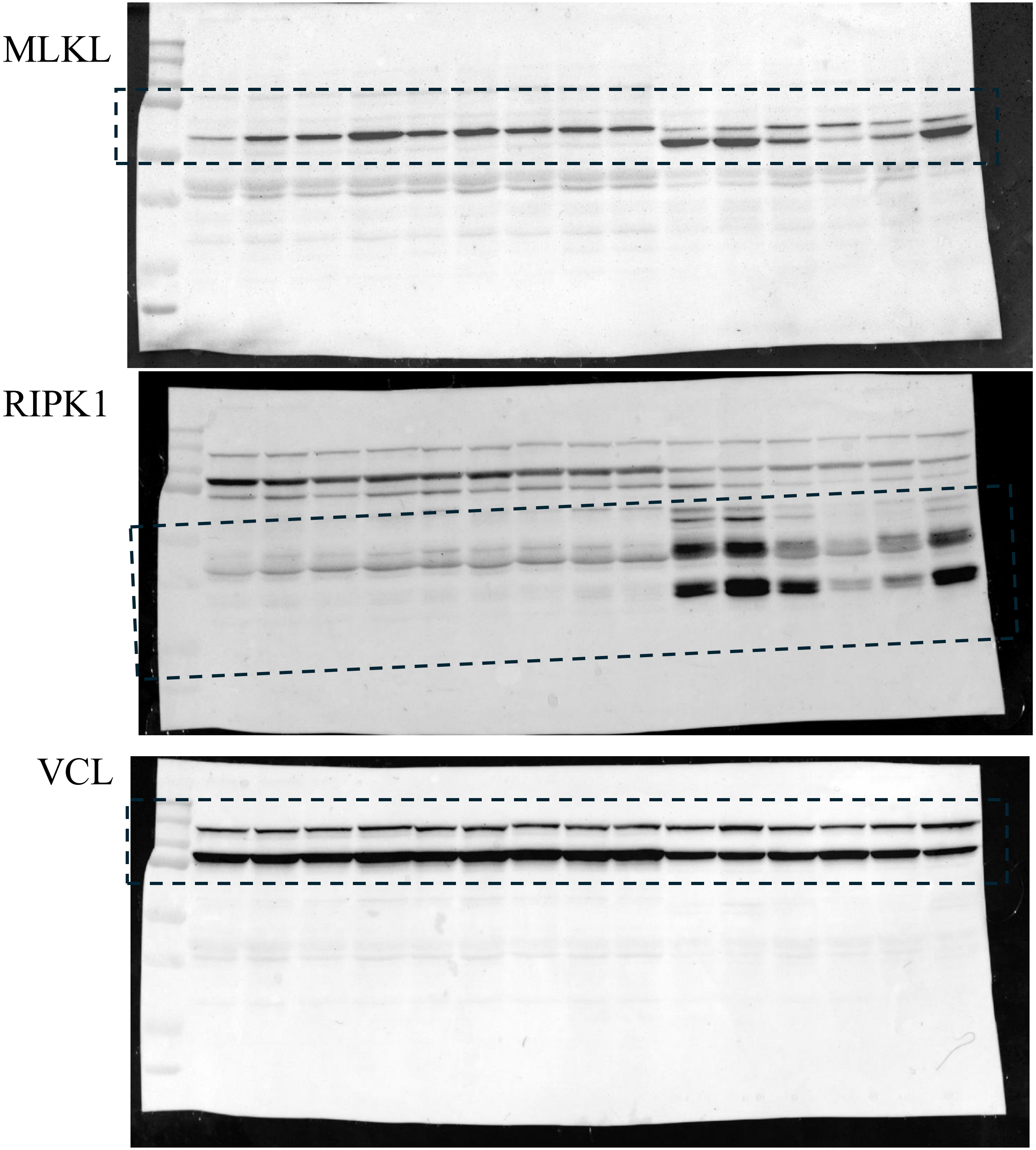

Figure 11

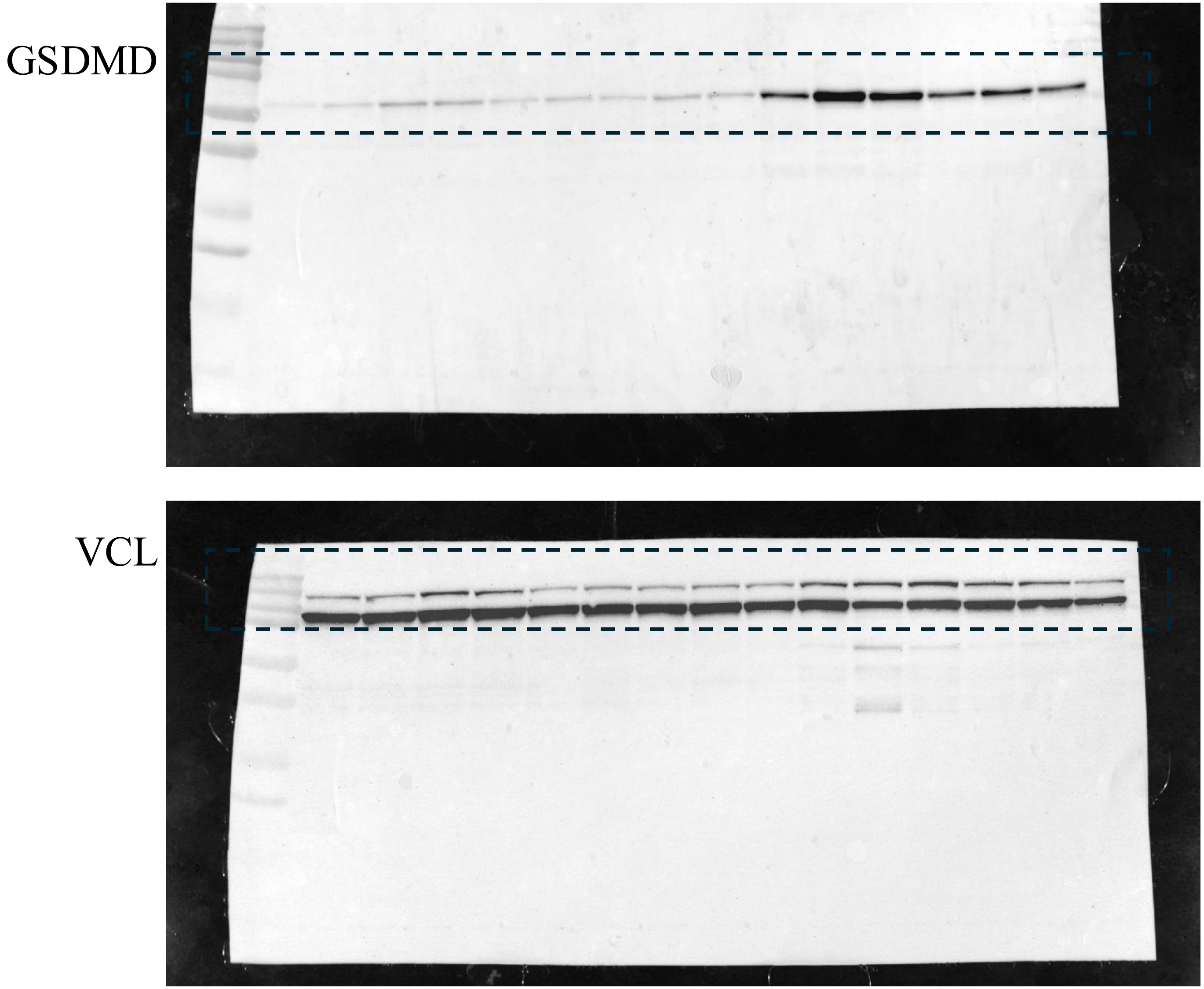

Figure 9

cardiomyocytes

CASP3

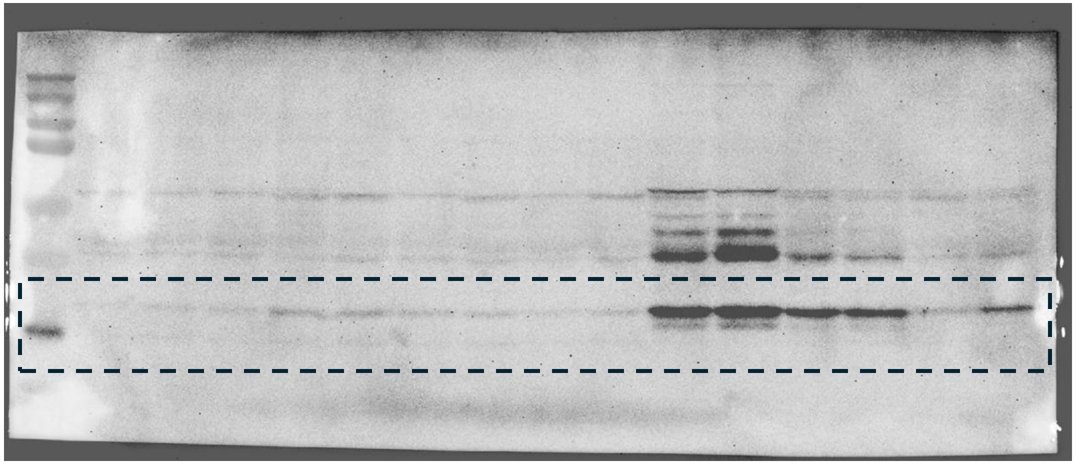

BAX

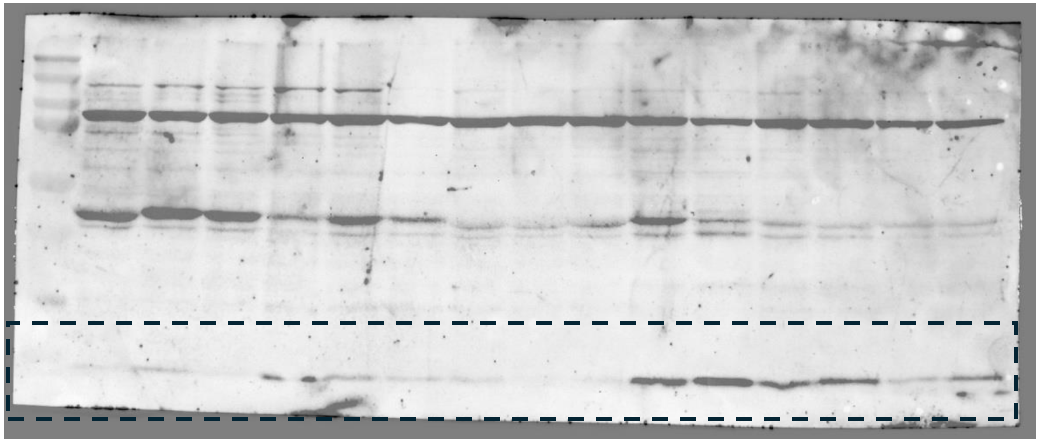

VCL

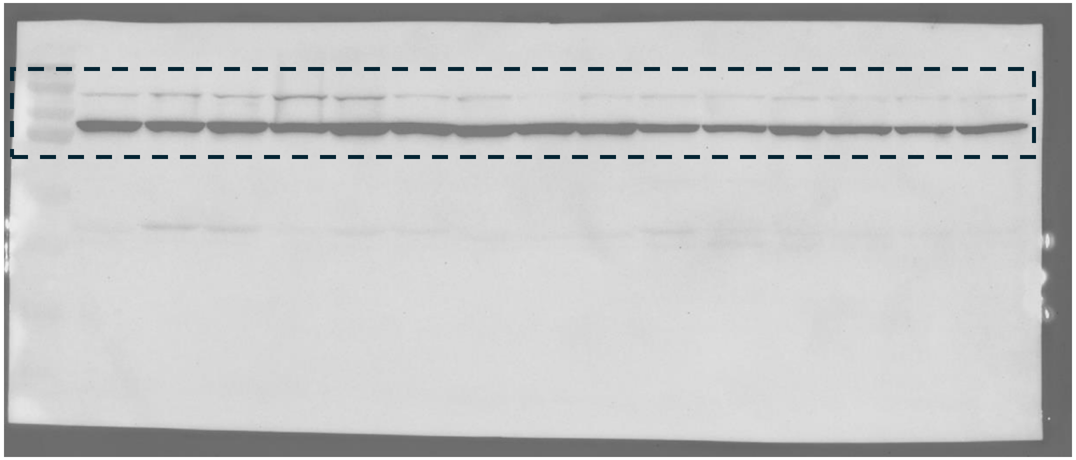

Figure 12

cardiomyocytes

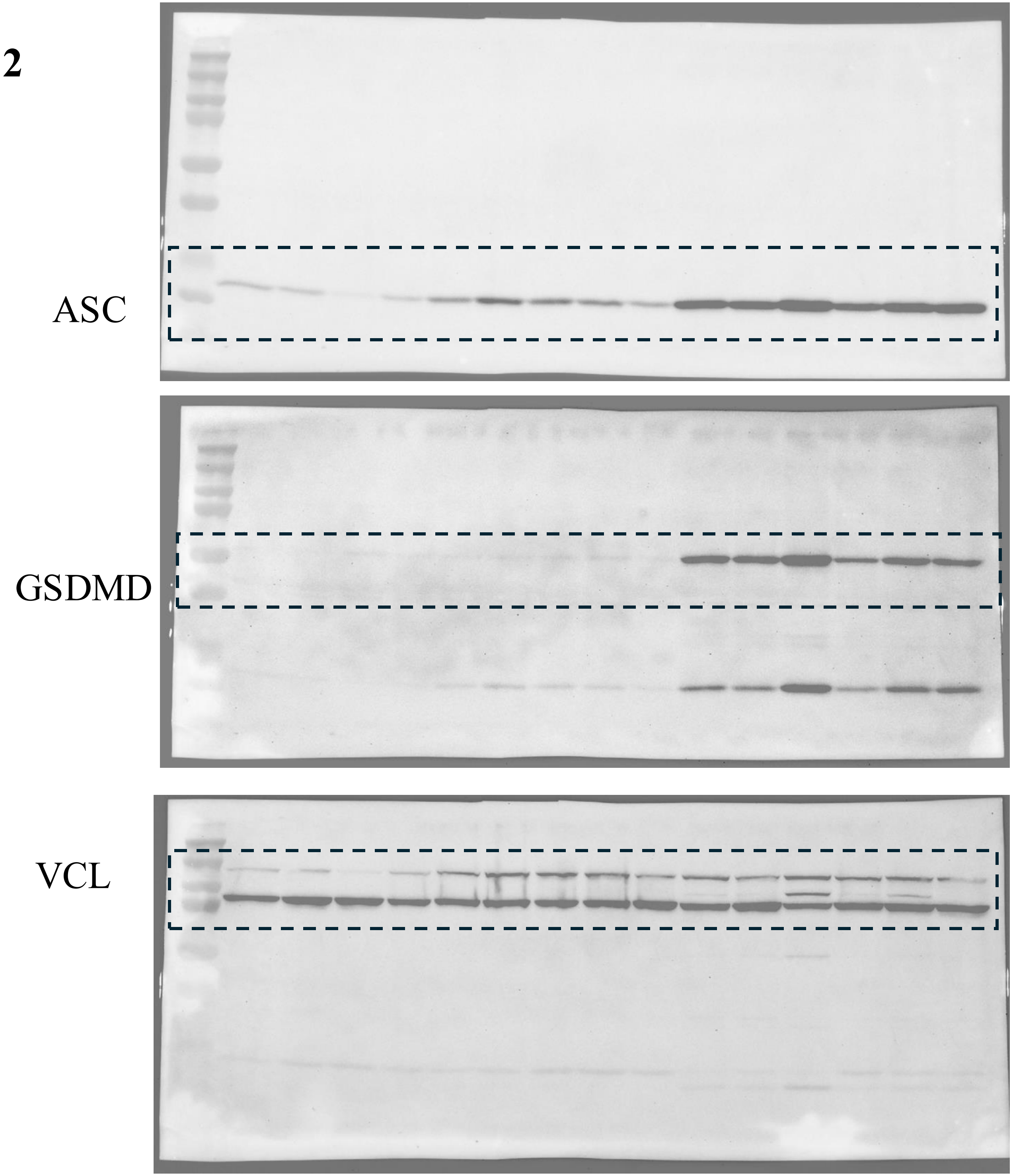

Figure 12

Non-myocytes

CASP3

BAD

VCL

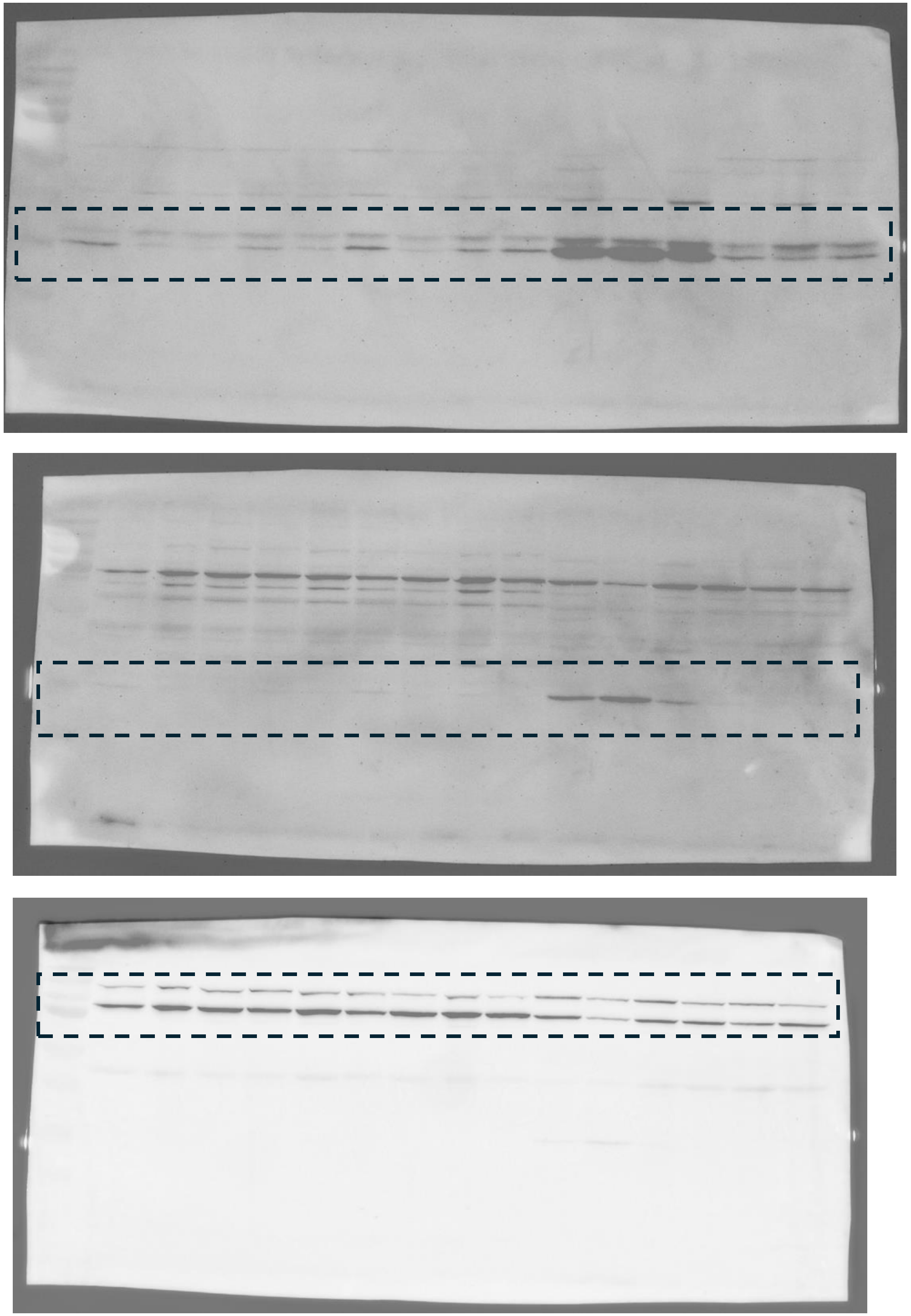

Figure 12

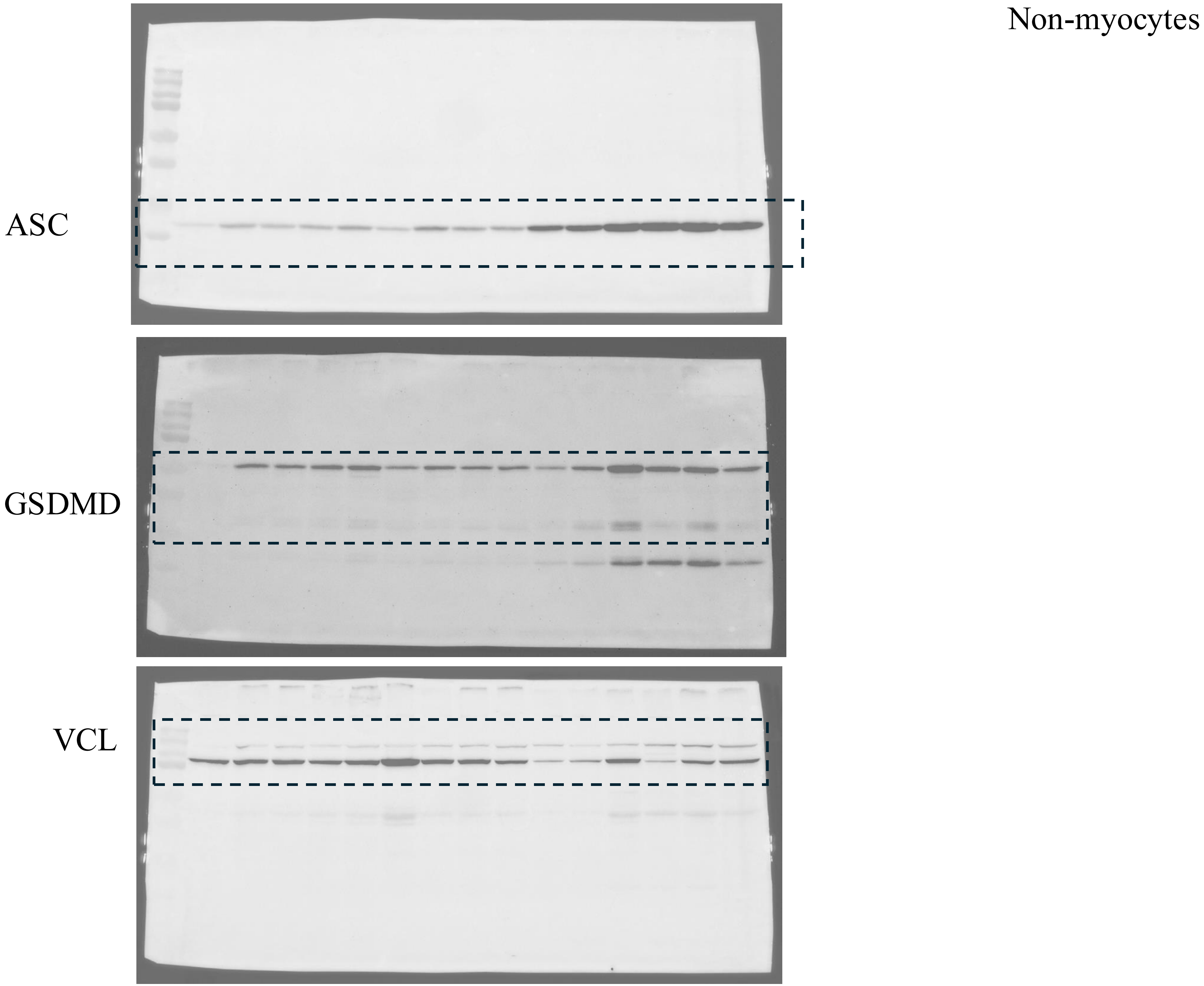

Figure 13

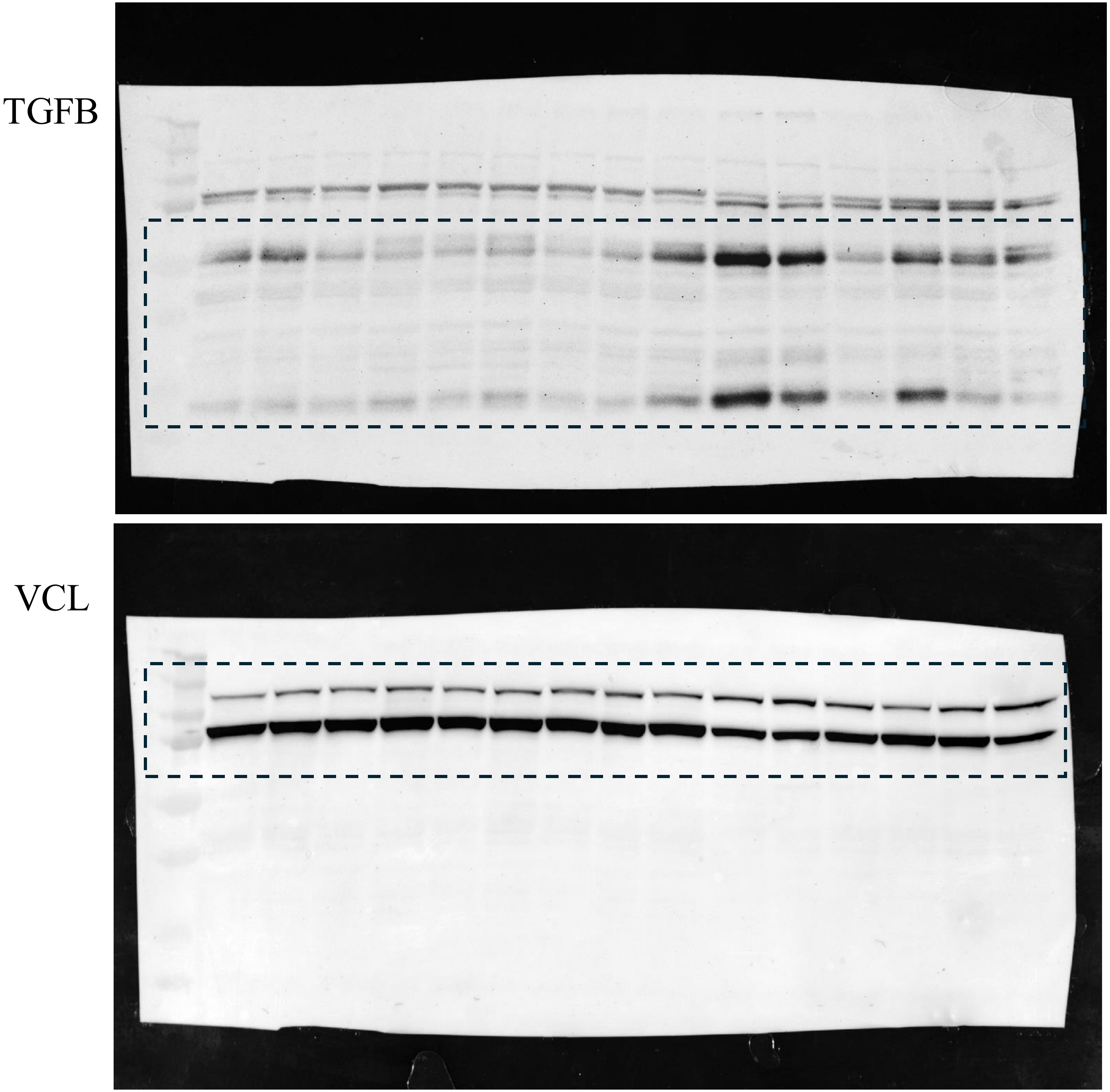

Supplement: Unedited blot and gel images [file jciinsight-10-192283-s076.pdf]
